# Transcription factor and microRNA interactions in lung cells: an inhibitory link between *NK2 homeobox 1*, *miR-200c* and the developmental and oncogenic factors *Nfib* and *Myb*

**DOI:** 10.1186/s12931-015-0186-6

**Published:** 2015-02-13

**Authors:** Jean-Bosco Tagne, Omar R Mohtar, Joshua D Campbell, Meenakshi Lakshminarayanan, Jingshu Huang, Anne C Hinds, Jining Lu, Maria I Ramirez

**Affiliations:** The Pulmonary Center, Boston University School of Medicine, Boston, USA; Section of Computational Biomedicine, Department of Medicine, Boston University School of Medicine, Boston, USA; Department of Pathology and Laboratory Medicine, Boston University School of Medicine, 72 E. Concord St, Boston, MA 02118 USA

**Keywords:** microRNA, Transcription factors, Gene expression, Lung epithelial cells, Targets

## Abstract

**Background:**

The transcription factor NK2 homeobox 1 (Nkx2-1) plays essential roles in epithelial cell proliferation and differentiation in mouse and human lung development and tumorigenesis. A better understanding of genes and pathways downstream of Nkx2-1 will clarify the multiple roles of this critical lung factor. Nkx2-1 regulates directly or indirectly numerous protein-coding genes; however, there is a paucity of information about Nkx2-1-regulated microRNAs (miRNAs).

**Methods and results:**

By miRNA array analyses of mouse epithelial cell lines in which endogenous Nkx2-1 was knocked-down, we revealed that 29 miRNAs were negatively regulated including miR-200c, and 39 miRNAs were positively regulated by Nkx2-1 including miR-1195. Mouse lungs lacking functional phosphorylated Nkx2-1 showed increased expression of miR-200c and alterations in the expression of other top regulated miRNAs. Moreover, chromatin immunoprecipitation assays showed binding of NKX2-1 protein to regulatory regions of these miRNAs. Promoter reporter assays indicated that 1kb of the miR-200c 5′ flanking region was transcriptionally active but did not mediate Nkx2-1- repression of miR-200c expression. 3′UTR reporter assays support a direct regulation of the predicted targets Nfib and Myb by miR-200c.

**Conclusions:**

These studies suggest that Nkx2-1 controls the expression of specific miRNAs in lung epithelial cells. In particular, we identified a regulatory link between Nkx2-1, the known tumor suppressor miR-200c, and the developmental and oncogenic transcription factors Nfib and Myb, adding new players to the regulatory mechanisms driven by Nkx2-1 in lung epithelial cells that may have implications in lung development and tumorigenesis.

**Electronic supplementary material:**

The online version of this article (doi:10.1186/s12931-015-0186-6) contains supplementary material, which is available to authorized users.

## Background

The NK2 homeobox 1 (Nkx2-1, Ttf1, T/ebp) gene controls lung, thyroid and brain gene expression in development and tumors [1-3]. In lung development, Nkx2-1 is essential for epithelial branching morphogenesis and bronchiolar and alveolar epithelial cell differentiation [2,3]. Mutations of *NKX2-1* lead to lung epithelial hyperplasia, interstitial disease, and postnatal respiratory distress [4]. In tumors, NKX2-1 has oncogenic and tumor suppressor functions, depending on the cell context, suggesting a dual role as a lineage specific factor contributing to lung cancer progression [5-8]. The downstream genes controlled by Nkx2-1 mediate its multiple functions in different cell contexts. In previous genome-wide studies we and others identified Nkx2-1 regulated protein-coding genes (mRNA) and Nkx2-1 direct binding targets [9-13] in mice and humans. However, non-coding microRNAs (miRNAs) regulated by Nkx2-1 have not been identified. Regulation of gene expression by miRNAs is a major mechanism of gene silencing [14], that controls translation and stability of target mRNAs in a cell and tissue specific manner. In the lung, the expression patterns and functions of specific miRNAs have been described during cell differentiation, development and in diseases such as lung fibrosis and cancer [14-16]. In development, specific miRNAs are differentially regulated over time and between sexes [17]; the miR-17-92 cluster plays important roles in cell differentiation and growth [18,19], whereas the Gata6-regulated cluster miR-302-367 [20] controls multiple aspects of lung endoderm progenitor cell behavior. Several microRNAs including miR-29, miR-365, and miR-17-92 [14,19] control tumor cell proliferation, invasion and survival. However, the link between the key lung transcription factor Nkx2-1, downstream miRNAs and their predicted targets has not been addressed. In this study we have characterized miRNAs regulated by Nkx2-1 in a mouse lung cell line system by genome-wide analysis of mRNA and miRNA profiles and confirm the expression patterns of highly regulated miRNAs in normal mouse lung and in lungs expressing phosphorylation mutant Nkx2-1. In particular, we found a regulatory link between Nkx2-1, miR-200c and the nuclear factor I/B (Nfib) and myeloblastosis oncogene (Myb). These findings add new components to the gene regulatory network controlled by Nkx2-1 in lung epithelial cells that may have implications in the various roles of Nkx2-1 in development and disease.

## Methods

### Cell lines and tissues

**Figure 1:**
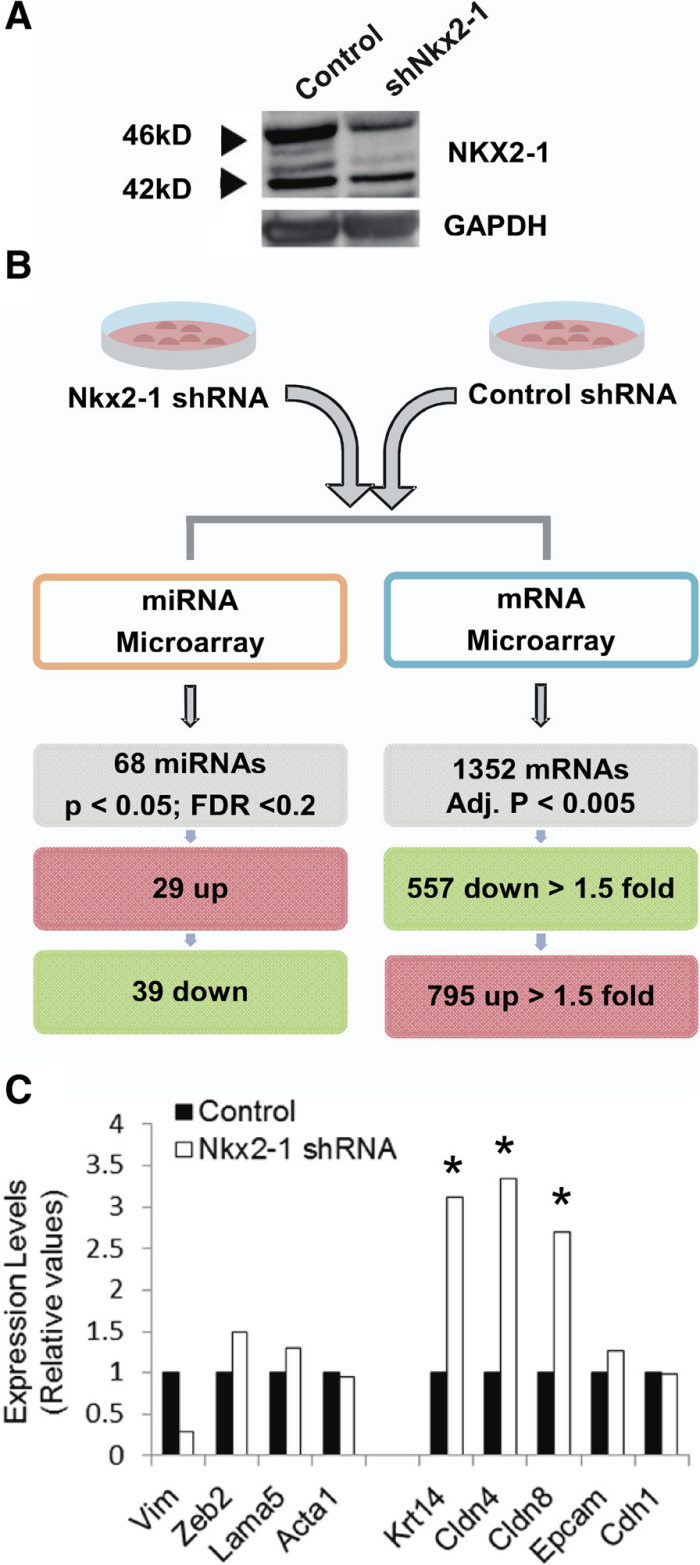
**Genome-wide analysis of miRNA and mRNA in Nkx2-1-knockdown cells. A**. Representative western blot analysis of NKX2-1 protein levels in Nkx2-1-shRNA treated MLE15 cells compared to non-silencing control. These stable cell lines were fully characterized in Cao et al [22], and Tagne et al, [12]. **B**. Diagram of the transcriptomic analyses indicating the number of genes identified up- or down-regulated (p < 0.05; FDR < 0.2). **C**. Expression levels of epithelial and mesenchymal markers in Nkx2-1shRNA treated MLE15 cells relative to non-silencing control, determined by microarray analysis. Adjusted p value < 0.0005.

The Murine Lung Epithelial cell line (MLE15), a gift of Dr.J.A. Whitsett (Cincinnati Children’s Hospital Medical Center), is derived from transgenic mice harboring the simian virus 40 large tumor antigen under the transcriptional control of the 3.7 kb human Surfactant Protein C promoter. These mice develop pulmonary adenocarcinomas within 4–6 months of age [21]. We have previously reported [12,22] the generation of three independent MLE15 stable cell lines transduced with lentivirus expressing Nkx2-1-shRNA or non-silencing control, followed by puromycin selection. These original cell lines were maintained in liquid nitrogen and expanded for the current studies. In these cells, Nkx2-1 mRNA levels are reduced to 60% of the control and NKX2-1 protein to ~40% (Figure 1A and [12]). Embryonic mouse tissues were dissected from phosphorylation-mutant Nkx2-1 mice (kindly provided by Dr. DeFelice (Università degli Studi di Napoli), harboring seven serine phosphorylation sites mutations in the Nkx2-1 locus [23]. These mutations prevent NKX2-1 protein to regulate genes controlling distal lung epithelial differentiation. Studies in mice were approved by the Boston University IACUC panel.

### microRNA and mRNA microarray experiments

Total RNA, isolated from three independently transduced Nkx2-1-shRNA and control MLE15 cell lines [12], was enriched in low molecular weight RNA using miRNeasy kit (Qiagen, Valencia, CA). RNA was labeled with FlashTag kit (Genisphere Inc., Hatfield, PA), and was hybridized to miRNA Galaxy arrays (Affymetrix, Santa Clara, CA). Arrays were scanned using Affymetrix GeneArray Scanner 3000 7G Plus. miRNA QC Tool software version 1.0.33.0 was used for background subtraction, detection p-value calculation and normalization. A two-sample *t*-test was performed to identify differentially regulated miRNA expression in Nkx2-1 knockdown cells; the Benjamini-Hochberg False Discovery Rate (FDR) was used to correct for multiple hypothesis comparisons [24]. Genes with a *p* < 0.05 and an FDR adjusted *p* value < 0.2 were considered to be differentially expressed.

For mRNA expression analysis we followed the GeneChip® Whole Transcript (WT) Sense Target Labeling Assay Manual (Affymetrix, Santa Clara, CA) as described previously [25]. The labeled fragmented DNA was hybridized to the Gene Arrays 1.0ST. After scanning, data were summarized using Affymetrix Expression Console (version 1.1). Robust Multi-Array Analysis algorithm [26] was used to generate gene-level data. A two-sample *t*-test was performed to identify differentially regulated mRNA expression in Nkx2-1 knockdown cells adjusted for multiple hypothesis comparisons [24]. Genes with an adjusted p < 0.0005 were considered significant. Both mRNA and microRNA expression data are deposited in the Gene Expression Omnibus GSE47055. Predicted targets of miR-200c were downloaded from TargetScanMouse 6.2 (aggregate PCT > 0.1) [27]. Gene Ontology database analysis was performed using GATHER (Gene Annotation Tool to Help Explain Relationships) [28]. Detailed microRNA and mRNA array methods are described in Additional file 1.

### Real time RT-PCR

mRNA expression was analyzed by RT-qPCR in total RNA samples using methods described previously [22]. miRNA expression was analyzed in 1μg of total RNA, reverse transcribed using MicroRNA Reverse Transcription Kit (Applied Biosystems, Grand Island, NY). miRNA RT-qPCR analyses were performed with MicroRNA Assays (Applied Biosystems) in a StepOne Real-Time PCR System (Applied Biosystems). We used rnuRNA-6B in cell lines and snoRNA-202 in mouse tissues since the expression of these endogenous genes was more stable in the conditions tested in each system. Quantitative analysis was performed by the 2^-ΔΔCt^ method.

### Chromatin immunoprecipitation assays

Chromatin immunoprecipitation assays (ChIPs) were performed as described previously [12], using 1 × 10^6^ cells and 10 μl of NKX2-1 antibody (07-601-Upstate, Millipore, Billarica, MA) or IgG control (Santa Cruz Biotechnology, Dallas, TX). Equal volumes of immunoprecipitated DNA solution and 10% of the input DNA fragments were amplified by qPCR using a custom designed TaqMan assay (Applied Biosystems) within -1kb relative to the first nucleotide of the pre-miRNA sequence indicated in the UCSC Genome Browser mm10 [29] (miR-1195, chr17:70860551–70861600; miR-200c, chr6:124718366–124719390) and quantified using TaqMan Master Mix (Applied Biosystems). Data were normalized to IgG and expressed as percentage of the input. Detailed ChIP methods are described in Additional file 1.

### miRNA promoter cloning, transfections and luciferase assays

Genomic regions (1.1 kb) 5′ to the first nucleotide of miR-200c and of miR-221 pre-miRNA sequences were retrieved from the UCSC Mouse Genome Browser [29]. We used oligonucleotides with restriction enzyme adaptors to amplify ~ 0.9-1kb of each region by PCR (miR-200c F 5′-SacI CAGGCAGACACTGCCATCT-3′, R 5′-HindIII CTACCCAACCAGTCCACCTCC-3′; miR-221 F 5′-SacI AGGAGAGGCCCTTGGTATAG-3′, R 5′-HindIII GTTCAGCCTGCAAATTATCC). Amplicons were ligated into the pGL3-basic luciferase vector (Promega, Madison, WI) and confirmed by sequencing. After several attempts we were unable to clone the 5′flanking region of miR-1195. Thus, miR-200c-Luc and miR-221-Luc plasmids were transiently transfected using Lipofectamine 2000 (Life Technologies, Grans Island, NY) in the cell lines described above. A renilla luciferase expressing vector was co-transfected for transfection efficiency control. Cells were harvested 48 h after transfection and luciferase activity measured using the Dual-Luciferase Reporter Assay System (Promega). Firefly luciferase signal was normalized to Renilla luciferase and data expressed relative to the pGL3-basic vector.

### 3′-UTR luciferase assays

All plasmids and reagents were obtained from Genecopeia (Rockville, MD). Plasmids harboring a Firefly luciferase reporter and the 3′UTR flanking region of the mir-200c predicted targets Myb (MmiT029722-MT01), Nfib (MmiT027729a2-MT01), and Six1 (MmiT028207-MT01), or a control vector (CmiT000001-MT01) were co-transfected in MLE15 cells either with a pre-miR-200c plasmid (MmiR3304-MR01) or with an scrambled control clone (CmiR0001-MR01) using EndoFectin Plus transfection kit. Different ratios of 3′UTR plasmids and miR-200c plasmid were evaluated (data not shown). A 1:5 ratio rendered the most consistent effect with all plasmids. Renilla luciferase expressing vector was co-transfected for transfection efficiency control. Cells were harvested 48 h after transfection and luciferase activity was measured using the Luc-Pair miR Luciferase Assay. Firefly luciferase signal was normalized to Renilla luciferase and data expressed relative to the Control Luciferase vector.

### miRNA-1195 antagomir assay

MLE15 cells were transduced with miRVana miR-1195 inhibitor (4464084 ID MH13628 (Life Technologies) using Lipofectamine RNAiMAX (Life Technologies) protocol.

### Statistical analysis

Data were obtained from at least three independent experiments (N = 3) and presented as mean ± SEM. The significance of differences was calculated using *t*-test for two-group unpaired comparisons. *P* < 0.05 was considered statistically significant.

## Results

### miRNAs downstream of Nkx2-1 in lung epithelial cells

**Figure 2:**
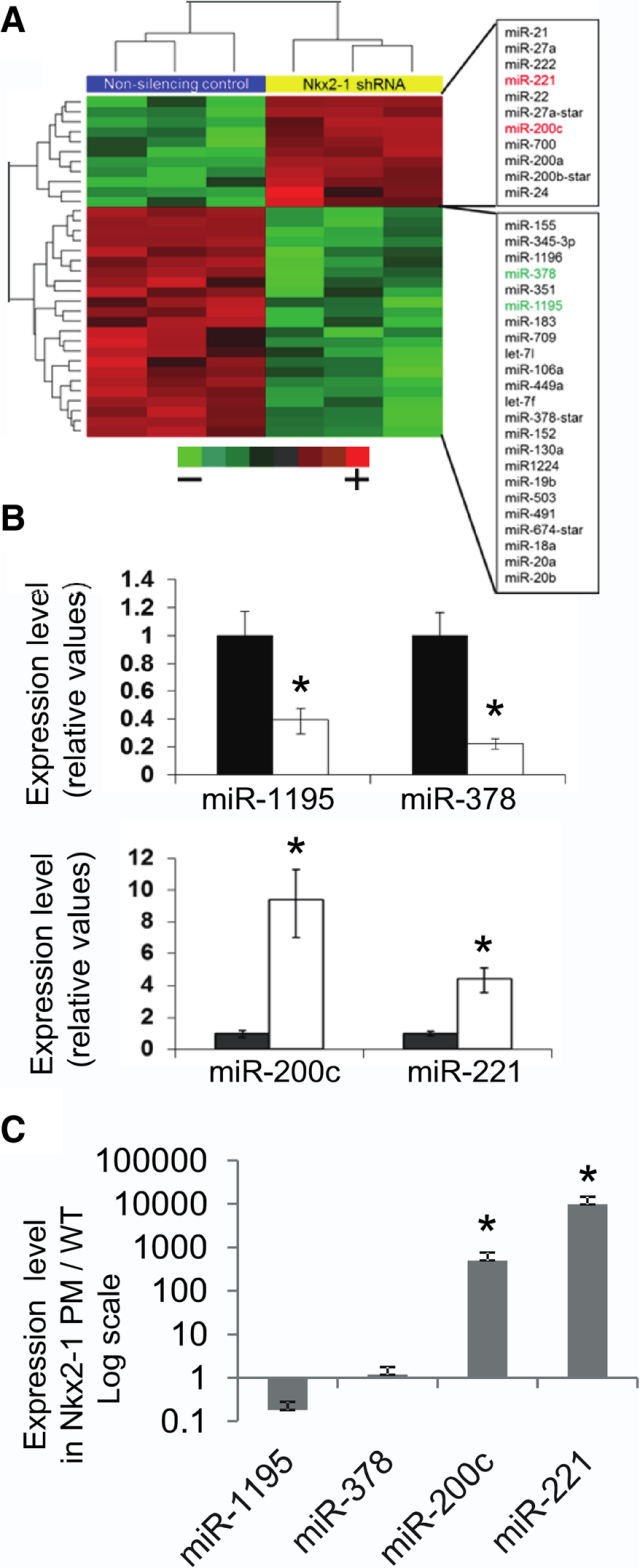
**Nkx2-1 controls the expression of specific miRNAs. A**. Hierarchical two-dimensional clustering analysis of Nkx2-1-regulated miRNA expression in MLE15 cells. Blue, Non-silenced Nkx2-1 samples; yellow, Nkx2-1-shRNA knocked-down samples. Each row corresponds to a miRNA and each column corresponds to an independent sample. Green represents lower and red higher relative expression. **B**. RT-qPCR validation analysis of miRNAs down- regulated (upper panels) or up-regulated (lower panels) by Nkx2-1 knockdown in MLE15 cells. Data was normalized to rnuRNA-6B. (*) p < 0.05. **C**. RT-qPCR analysis of miRNA expression levels in Nkx2-1PM/PM relative to Nkx2-1+/+. Data was normalized to snoRNA-202 and represented in a log scale. (*) p < 0.05.

To identify candidate miRNAs regulated by Nkx2-1 we analyzed by microRNA arrays differences in expression levels in the small RNA fraction isolated from control and Nkx2-1-shRNA transduced MLE15 cells (Figure 1A, B). We have previously characterized these stable cell lines in two publications [12,22]. Nkx2-1 mRNA was knocked-down to 60% of control and protein to 40% (Figure 1A and [12]). Reduction of Nkx2-1 delayed cell cycle progression by halting cells in G2/M phase of the cell cycle [12]. We now show that these moderate changes in Nkx2-1 expression also produced significant changes in miRNA and gene expression. Importantly, Nkx2-1 knock-down MLE15 cells retain their epithelial features including unchanged expression of Cdh1, and increased Krt14, Cldn4, Cldn8 and Epcam (Figure 1C). Being of epithelial origin they have low expression of vimentin, unchanged Zeb2 (Figure 1C) and undetectable mRNA levels of the Epithelial-Mesenchymal-Transition mediators Zeb1, Snail, Snug or Twist determined by microarray analysis. We identified 68 well-annotated miRNAs significantly altered by down-regulation of Nkx2-1 (p < 0.05; FDR < 0.2) (Table 1). From these, 29 miRNAs increased their expression levels by Nkx2-1 knock-down including miR-200c (16.7 fold), miR-200b (1.7 fold), miR-221 (4.2 fold), and miR-222 (3.7 fold) (Figure 2A and Table 1). A group of 39 miRNAs was significantly down-regulated by Nkx2-1 knock-down including miR-1195 (−4.9 fold), miR-378 (−4.6 fold), miR-449a (−2.1 fold), and miR-130a (−1.9 fold) (Figure 2A and Table 1). Other miRNAs regulated by Nkx2-1 include miRNAs belonging to the miR-106-363 cluster (miR-106a/b, miR-18a, miR-19b-1/2 and miR-20a/b) (Figure 1 and Table 1). The expression of miR-141, clustered and usually co-expressed with miR-200c [30], is higher in Nkx2-1-shRNA than in control (p = 0.0196; FDR = 0.087) (Table 1) following the same trend than miR-200c. Expression patterns of the most altered miRNAs (miR-200c, miR-221, miR-1195, and miR-378) were analyzed in Nkx2-1 knock-down cells by RT-qPCR (Figure 2B) confirming the microarray data.

**Table 1:** **miRNAs differentially expressed in Nkx2-1shRNA vs non-silencing control in MLE15 cells**

**MicroRNA**	**Fold change**	**p (Ttest)**	**FDR**	**MicroRNA**	**Fold change**	**p (Ttest)**	**FDR**
mmu-miR-200c_st	16.73	0.000005	0.001	mmu-miR-1195_st	−4.90	0.0033	0.034
mmu-miR-221_st	4.16	0.0022	0.027	mmu-miR-378_st	−4.58	0.00004	0.005
mmu-miR-222_st	3.71	0.0004	0.013	mmu-miR-351_st	−3.12	0.0056	0.044
mmu-miR-27a-star_st	2.44	0.0017	0.024	mmu-miR-503_st	−2.65	0.0003	0.012
mmu-miR-125a-5p_st	2.05	0.0217	0.090	mmu-miR-449a_st	−2.13	0.0007	0.014
mmu-miR-24-2-star_st	2.00	0.0162	0.081	mmu-miR-1196_st	−2.07	0.0005	0.013
mmu-miR-27a_st	1.80	0.0006	0.013	mmu-miR-685_st	−2.05	0.0330	0.120
mmu-miR-486_st	1.75	0.0182	0.085	mmu-miR-709_st	−2.00	0.0073	0.049
mmu-miR-200b-star_st	1.72	0.0054	0.044	mmu-miR-1224_st	−1.94	0.0070	0.049
mmu-miR-574-3p_st	1.69	0.0085	0.052	mmu-miR-130a_st	−1.93	0.0057	0.044
mmu-miR-200b_st	1.65	0.0104	0.057	mmu-miR-20a_st	−1.86	0.0010	0.017
mmu-miR-22_st	1.49	0.0041	0.038	mmu-miR-20b_st	−1.75	0.0009	0.017
mmu-miR-484_st	1.48	0.0470	0.159	mmu-miR-19b_st	−1.71	0.0073	0.049
mmu-miR-700_st	1.47	0.0004	0.013	mmu-miR-18a_st	−1.69	0.0021	0.026
mmu-miR-344_st	1.45	0.0168	0.082	mmu-miR-152_st	−1.64	0.0033	0.034
mmu-miR-23a_st	1.45	0.0184	0.085	mmu-miR-805_st	−1.58	0.0197	0.087
mmu-miR-181a_st	1.44	0.0143	0.073	mmu-miR-185_st	−1.52	0.0003	0.012
mmu-miR-200a_st	1.39	0.0033	0.034	mmu-miR-106b_st	−1.51	0.0284	0.108
mmu-miR-181b_st	1.36	0.0088	0.052	mmu-miR-27b_st	−1.48	0.0085	0.052
mmu-miR-205_st	1.36	0.0097	0.055	mmu-miR-106a_st	−1.48	0.0061	0.045
mmu-miR-532-3p_st	1.32	0.0220	0.090	mmu-miR-346_st	−1.48	0.0260	0.101
mmu-miR-151-5p_st	1.31	0.0171	0.082	mmu-miR-877_st	−1.45	0.0118	0.061
mmu-miR-362-5p_st	1.31	0.0208	0.088	mmu-let-7i_st	−1.44	0.0047	0.040
mmu-miR-21_st	1.28	0.0014	0.020	mmu-miR-182_st	−1.43	0.0083	0.052
mmu-miR-24_st	1.27	0.0045	0.040	mmu-miR-345-3p_st	−1.42	0.0005	0.013
mmu-miR-140-star_st	1.26	0.0233	0.094	mmu-miR-674_st	−1.40	0.0095	0.055
mmu-miR-28-star_st	1.23	0.0203	0.088	mmu-let-7f_st	−1.40	0.0034	0.034
mmu-let-7e_st	1.17	0.0113	0.060	mmu-let-7d_st	−1.40	0.0495	0.164
mmu-miR-141_st	1.17	0.0196	0.087	mmu-miR-324-5p_st	−1.36	0.0300	0.112
				mmu-miR-183_st	−1.36	0.0021	0.026
				mmu-miR-378-star_st	−1.34	0.0067	0.049
				mmu-miR-25_st	−1.33	0.0326	0.120
				mmu-miR-674-star_st	−1.32	0.0012	0.019
				mmu-miR-100_st	−1.25	0.0463	0.159
				mmu-miR-17-star_st	−1.23	0.0473	0.159
				mmu-miR-99a_st	−1.22	0.0258	0.101
				mmu-miR-491_st	−1.20	0.0002	0.012
				mmu-miR-191_st	−1.13	0.0432	0.152
				mmu-miR-470_st	−1.12	0.0350	0.125

(p < 0.05; FDR < 0.2).

### miRNA expression is altered in Nkx2-1 phosphorylation-mutant lungs

Nkx2-1 null mice have an extreme lung phenotype due to failing in branching morphogenesis resulting in a large sac lined by a simple epithelium [2,3]. Therefore, we tested the effect of Nkx2-1 on specific miRNAs in vivo in wild type and in Nkx2-1 phosphorylation-mutant lungs at E19.5 by RT-qPCR. These mice have normal branching morphogenesis but show alterations in distal epithelial differentiation and expression of lung function genes. By RT-qPCR analysis we showed that absence of phosphorylated NKX2-1 results in a significant increment in the levels of miR-200c and miR-221; miR-1195 is down-regulated although no significantly, showing a similar trend than in Nkx2-1 knockdown experiments in MLE15 cells (Figure 2C).

### NKX2-1 protein binds to miRNA regulatory regions

**Figure 3:**
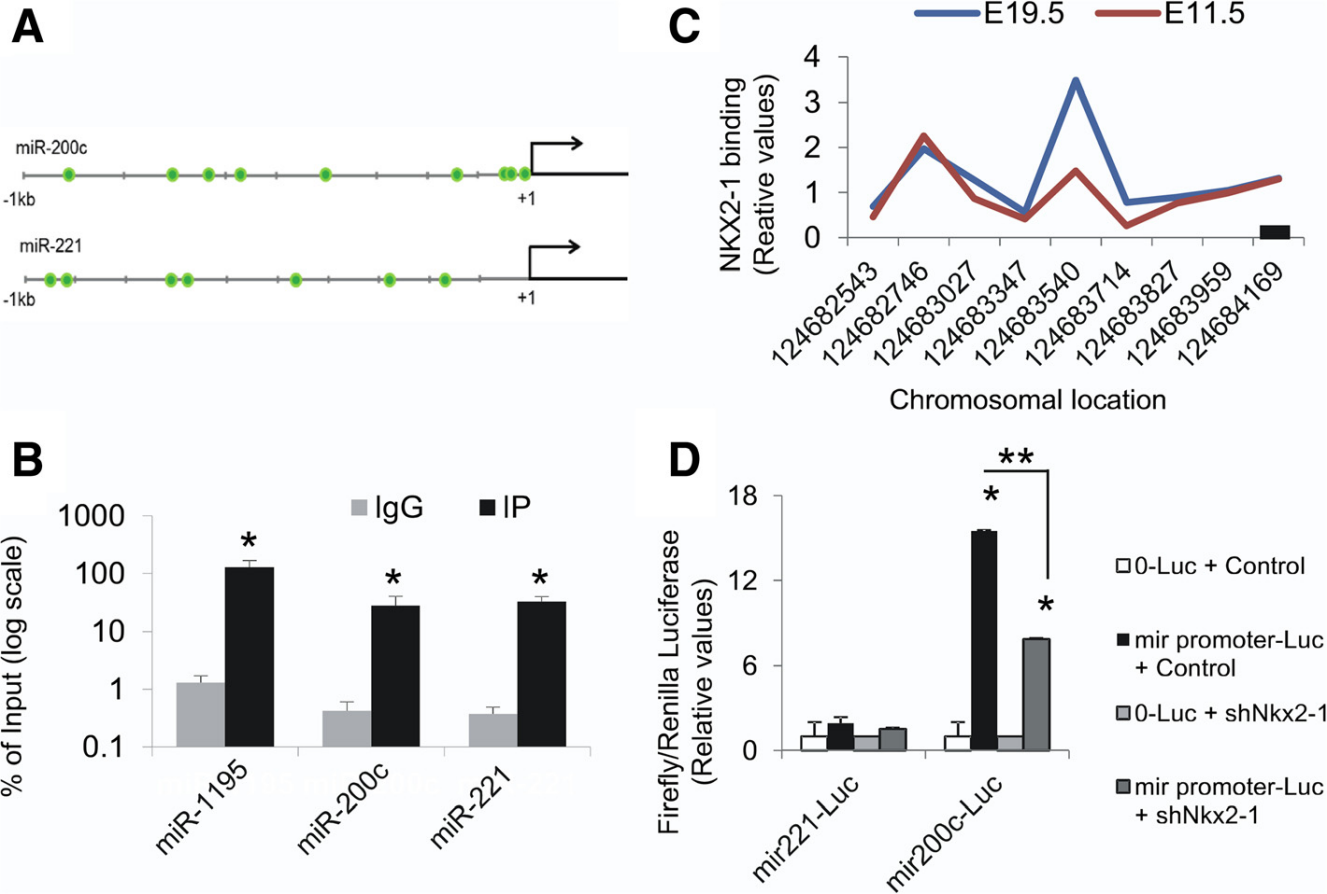
**NKX2-1 binds to miRNA’s regulatory regions and regulates their transcriptional activity. (A)** Scheme representing 1 kb of the 5′ flanking region of each miRNA. The core of the NKX2-1 consensus binding site (CAAG/CTTG) is represented as a green circle. **(B)** Chromatin immunoprecipitation assays using NKX2-1 antibody or IgG control followed by qPCR analysis of the 5′ flanking regions of miR-200c, miR-221 and miR-1195. Data represents percentage of the input in log scale. (*) p ≤ 0.05. **(C)** Binding of NKX2-1 to miR-200c 5′ flanking region in E11.5 and E19.5 mouse lung previously determined by ChIP-chip analysis [12]. Data represents binding signal (y axis) for each probe on the promoter tiling array (x axis) corresponding to the 5′ flanking region of the pre-miR-200c. miR-221 and miR-1195 5′ flanking regions were not represented in the array. Red, E11.5; blue, E19.5; black line, transcript. N = 3 **(D)** Promoter-luciferase analysis of the transcriptional activity of the 1 kb 5′ flanking regions of different miRNAs in MLE15 transduced with NKx2-1 shRNA or control. Data represents Firefly luciferase normalized to Renilla luciferase. Values are relative to the 0-Luciferase (pGL3) control vector in each cell line. (*) p < 0.05 in 0-Luc vs. miR-200c –Luc; (**) p < 0.05 in miR-200c-Luc + scrambled vs. miR-200c.

To evaluate NKX2-1 protein binding to regulatory regions of the top regulated miRNAs we performed ChIP assays using a NKX2-1 antibody or the corresponding IgG followed by quantitation by qPCR of fragments within the miRNA’s 5′ flanking regions (Figure 3A). The NKX2-1 antibody significantly immunoprecipitated 5′regions of the tested miRNAs compared to IgG (Figure 3B). For miR-200c, the enrichment of the IP fractions with the Nkx2-1 antibody and IgG were 28 and 0.4% of input while for miR-1195, were 129% and 1.3% of input respectively. NKX2-1 also binds to the 5′flanking region of miR-221. These data indicate a potential regulatory link between NKX2-1 and miR-200c, miR-221, or miR-1195. In support of these results, a search in a ChIP-chip dataset of NKX2-1 target genes in mouse lung development that we previously published [12] indicates a significant binding of NKX2-1 within the 1kb 5′ flanking region of the miR-200c gene (Figure 3C).

### Nkx2-1 controls transcriptional activity of the miR-200c 5′ flanking region

To evaluate the transcriptional activity of the 5′flanking regions of selected microRNAs we cloned ~1kb 5′ to the miR-200c and to the miR-221 transcripts in a luciferase reporter vector. Both fragments contain several NKX2-1 consensus binding core CAAG/CTTG sites (Figure 3A). The miR-200c 5′flanking fragment is highly conserved in humans where it is transcriptionally active [31]. We transiently transfected the mouse constructs in MLE15 cell lines previously transduced with lentivirus expressing Nkx2-1-shRNA or non-silencing control (Figure 3D). The 5′ flanking region of miR-200c exhibits intense transcriptional activity (15-fold ± 0.66; p = 0.0005) with normal levels of Nkx2-1. Unexpectedly, we found that knock-down of Nkx2-1 resulted in lower transcriptional activity of this 1kb fragment (8-fold ± 0.24; p = 1.6 × 10^−6^). The 1kb fragment 5′ to miR-221 was transcriptionally inactive in the same conditions (Figure 3D). So, Nkx2-1 has a strong effect in controlling the levels of miR-200c expression but this control might be indirect. Nkx2-1 is recognized to act mainly as a transcriptional activator [11], binding to the promoters/enhancers of 58% of activated downstream genes but only to 23% of genes repressed by Nkx2-1. Therefore, most genes repressed by Nkx2-1 do so by mechanisms other than direct promoter binding as may be the case for miR-200c. Alternatively, the elements mediating Nkx2-1 repression of miR-200c might be located in regulatory regions beyond the -1kb 5′ flanking region. Analysis of histone marks in distinct microRNA loci to identify putative transcription start sites (TSS) indicates that a putative TSS of miR-200c in mouse cells might be within 5 kb of the pre-miRNA sequence [32].

### Expression patterns of predicted targets of Nkx2-1-regulated miRNAs

**Figure 4:**
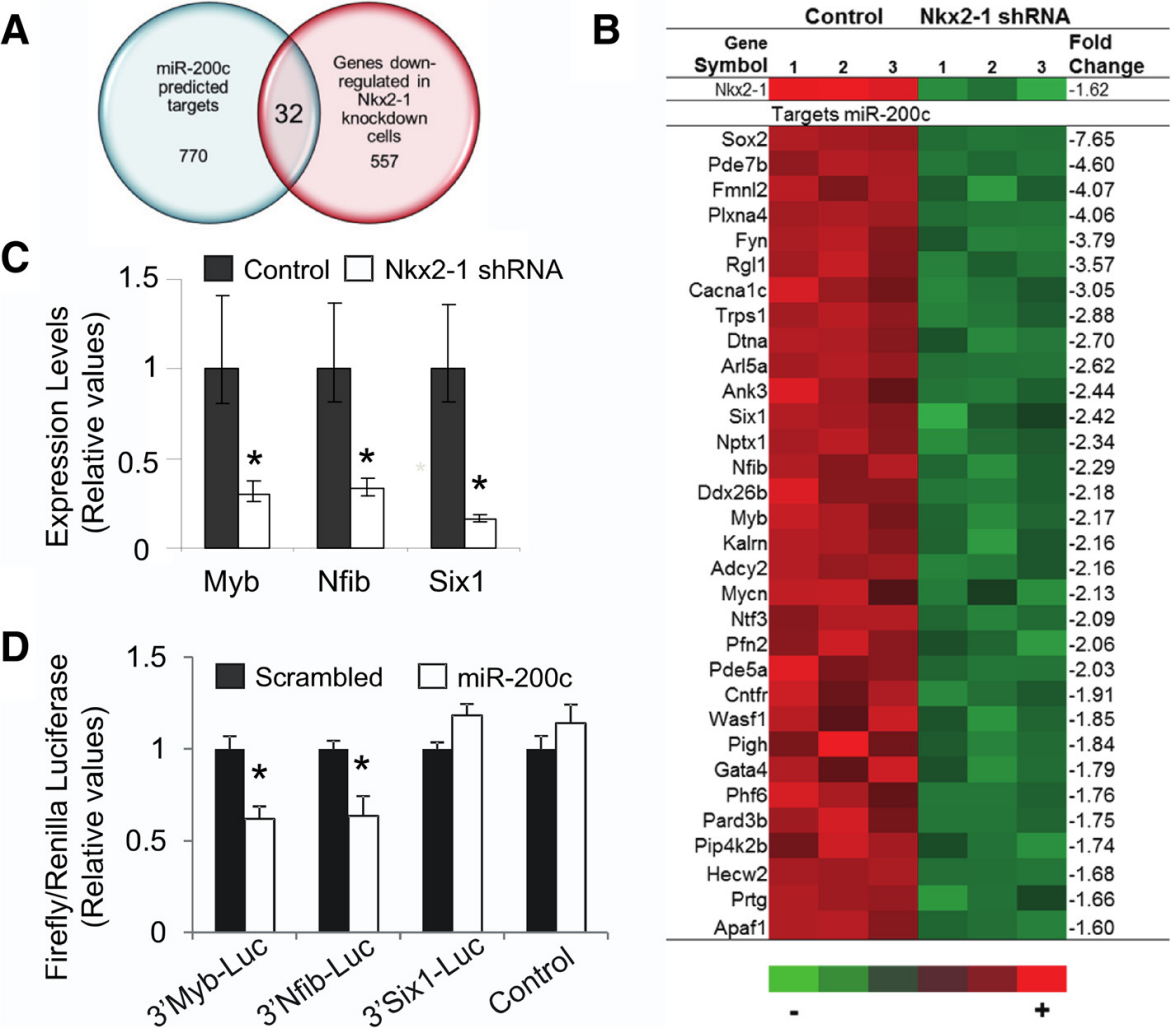
**Analysis of predicted targets of miR-200c. (A)** Venn diagram showing the intersection analysis of in silico miR-200c predicted target genes in TargetScanMouse 6.2 and genes downregulated in Nkx2-1 knockdown cells identified by microarray analysis. **(B)** Expression level of Nkx2-1 and of the 32 predicted targets of miR-200c identified in the intersection in A, determined by microarray analysis. FC, fold change; (1, 2, 3) biological replicates; red represents higher expression levels; green represents lower expression levels. Adjusted p value < 0.0005 **(C)** Selected transcription factors target of miR-200c were validated by RT-qPCR in Nkx2-1 shRNA treated cells vs. non-silencing control MLE15 cells. N = 3-6. (*) p < 0.01. **(D)** 3′UTR-Luciferase analysis of miR-200c predicted targets Myb, Nfib and Six1 compared to control-Luciferase in MLE15 transfected with pre-miR-200c or scrambled control. (*) p < 0.05.

Predicted targets of miR-200c were retrieved from TargetScanMouse (Release 6.2) [27]. A total of 770 predicted targets of miR-200c (total context score ≤ −0.01) were identified (Additional file 2: Table S1). We also determined genome-wide changes in mRNA levels in the Nkx2-1 knock-down cells using expression microarrays. 1352 genes with an adjusted p value < 0.0005, logFC < −0.6 or logFC > 0.6 were selected for further analysis (Additional file 3: Table S2). We intersected TargetScanMouse predicted targets with 557 genes showing anti-correlated expression to that of miR-200c (Figure 4A) in Nkx2-1 knockdown cells. The intersection showed 32 genes whose changes in expression were anti-correlated with miR-200c (including the transcription factors Gata4, Myb, Nfib Ntf3, Phf6, Six1, Sox2, and Trps1) (Figure 4B). We validated Myb, Nfib and Six1 expression patterns by RT-qPCR analysis (Figure 4C).

### miR-200c controls expression of its predicted targets Nfib and Myb

A significant number of miR-200c targets whose expression was negatively correlated to miR-200c in the microarray analysis were overrepresented in the transcriptional regulation GO category (Additional file 4: Table S3). The transcription factors Gata4 [33], Ntf3 [34] and Sox2 [35] are experimentally validated targets of miR-200c in human cells. We selected Nfib, Six1, and Myb to perform 3′UTR-luciferase reporter assays in MLE 15 cells expressing pre-miR-200c or scrambled control. Significant reduction of luciferase activity was observed in Nfib 3′UTR-Luciferase (0.63 ± 0.06; p = 0.016) and in Myb 3′UTR-Luciferase (0.62 ± 0.04; p = 0.002) transfected cells in the presence of pre-miR-200c compared to the scrambled control (Figure 4D). No difference in luciferase activity was observed in cells co-transfected with Six-1-Luciferase and pre-miR-200c or scrambled plasmids.

### miR-1195 controls expression of its predicted targets

**Figure 5:**
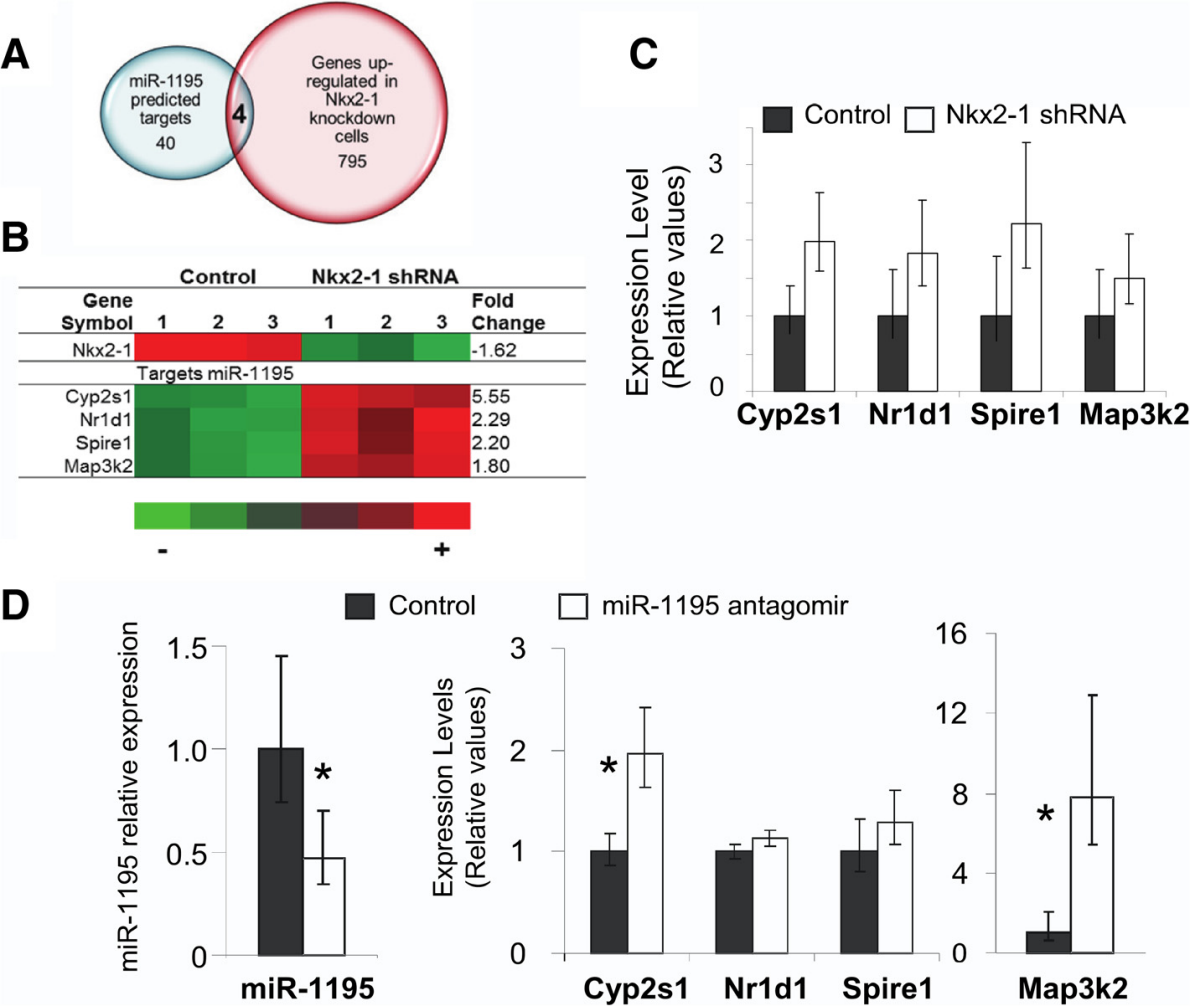
**Analysis of predicted targets of miR-1195. (A)** Venn diagram of the intersection of in silico miR-1195 predicted target genes in TargetScanMouse 6.2 and genes upregulated in Nkx2-1 knockdown cells identified by microarray analysis. **(B)** Expression level of Nkx2-1 and of the 4 predicted targets of miR-1195 in Nkx2-1shRNA treated MLE15cells vs. non-silencing control, determined by microarray analysis. FC, fold change; (1, 2, 3) biological replicates; red represents higher expression levels; green represents lower expression levels. **(C)** The 4 predicted targets of miR-1195 were validated by RT-qPCR in Nkx2-1 shRNA treated cells vs. non-silencing control MLE15 cells. (*) p < 0.05. **(D)** A miR-1195 inhibitor transduced in MLE15 cells reduces the expression of miR-1195 by 50% determined by RT-qPCR; expression of miR-1195 predicted target genes in the same cells inversely correlated to the levels of miR-1195 determined by RT-qPCR. (*) p < 0.05.

We intersected 40 predicted targets of miR-1195 retrieved from TargetScanMouse with 795 genes showing anti-correlated expression to that of miR-1195 (Figure 5A) in Nkx2-1 knockdown cells. We identified only 4 genes whose changes in expression induced by Nkx2-1 were anti-correlated with changes in miR-1195 expression (Nr1d1, Cyp2s1, Spire1 and Map3k2) (Figure 5B). We observed the same trend in the expression of these putative targets analyzed by RT-qPCR (Figure 5C).

To further evaluate the effect of miR-1195 on predicted downstream genes, we transfected MLE15 cells with a miR-1195 inhibitor to reduce its expression levels. After 48 h of transduction, expression levels of miR-1195 were measured by RT-qPCR confirming that miR-1195 was reduced by 50% (Figure 5D). Expression of Map3k2 and Cyp2s1 was significantly up-regulated by the miR-1195 inhibitor (Figure 5D). Because miR-1195 is a mouse specific miRNA no further analyses were performed to determine direct regulation of the identified targets. hsa-miR-584 shares partial homology to mmu-miR-1195 [36] and will be analyzed in future studies.

## Discussion

In this study we have identified microRNAs whose expression is influenced by knock-down of Nkx2-1. Using genome- wide miRNA expression profiling in a lung adenocarcinoma-derived mouse epithelial cell line (MLE15) [21], we observed that reduction of Nkx2-1 levels to approximately half of that in control cells promoted significant and reproducible changes in miRNA expression patterns, including a high up-regulation of miR-200c. The top up- and down- regulated miRNAs are expressed in normal mouse fetal lung and their level of expression is also altered in mice lacking functional phosphorylated-Nkx2-1. Furthermore, we present evidence of a regulatory link between Nkx2-1, mirR-200c and the downstream transcription factors Nfib and Myb. The studies indicate that down-regulation of Nkx2-1 de-represses miR-200c, either by a direct or an indirect mechanism. Downstream, miR-200c reduces the expression of its predicted targets Nfib and Myb.

miR-200c was initially identified as a lung-specific miRNA in rats [37]. Expression of miR-200c is higher in adult rat lung alveolar cells than fibroblasts, and its expression is lower during development than in adult lung. Most studies about miR-200c have been performed in the mesenchymal-epithelial transition (MET) context. One well studied function of the miR-200 family is the induction of an epithelial phenotype by inhibiting the transcriptional repressor Zeb2 and thereby enhancing E-cadherin expression [31,35]. Supporting its role in maintaining an epithelial phenotype in the lung, miR-200c expression is significantly reduced in the lung of mice with experimental fibrosis and in lungs of IPF patients where the epithelium undergoes profound alterations by acquiring some mesenchymal characteristics. In lung adenocarcinoma a high-level of NKX2-1 expression is significantly associated with longer overall survival [7], whereas a high-level of miR-200c expression is associated with shorter overall survival [30] supporting the inverse correlation. But in epithelial cell contexts, as the ones described in this work, increased miR-200c may regulate cellular processes other than MET, such as proliferation, or survival of lung cells.

Our data shows that miR-200c represses Nfib and Myb genes. These transcription factors [38,39] and other known miR-200c targets such as E2F3 [40] and Kras [41] have been linked to lung epithelial proliferation in development and in tumorigenesis acting as oncogenes. Nfib is highly expressed in the embryonic lung epithelium and in the mesenchyme, but its expression gets restricted to the epithelium at late gestation. Absence of Nfib in null mutant mice results in early postnatal lethality with severe lung hypoplasia [38]. In small cell lung carcinomas NFIB regulates cell viability and proliferation [42] and it is considered an oncogene. The other predicted target, Myb has been recently linked to the differentiation of airway epithelial cells [43], and was shown to be regulated by miR-200c in glioblastomas [44] and breast cancer cells [43]. In the latter, the human MYB 3′UTR was shown to contain miR-200c binding sites. Therefore, a reduction of Nkx2-1may modulate the proliferative activity of lung epithelial cells not only by direct inhibition of cyclin B, as it was previously described [12,13], but also through direct or indirect activation of miR-200c to inhibit downstream oncogenes.

We identified other miRNAs regulated by Nkx2-1 expression. miR-1195, for instance, is positively regulated by Nkx2-1 in the lung epithelial cell system and in normal vs. phosphorylation mutant lungs. miR-1195 is a mouse specific miRNA [45], enriched in epithelial structures in the embryo and moderately homologous to the human miRNA hsa-miR-584-3p (miRBase [36]). Predicted targets of miR-1195, including the mitogen-activated protein kinase Map3k2 [46] and the extra-hepatic cytochrome P450 enzyme Cyp2s1 [47,48], responded to changes in miR-1195 levels. Because miR-1195 is not found in humans, its close homolog hsa-miR-584-3p will be the focus of further studies.

## Conclusions

Overall, our findings suggest that modulation of the level of expression of Nkx2-1 has a high impact on downstream regulatory events mediated by miRNAs in mouse lung epithelial cell lines and in lung tissue. Particularly, miR-200c, negatively regulated by Nkx2-1, reduces the expression of downstream targets Nfib and Myb. Because of the high conservation between mouse and human homologs of Nkx2-1, Nfib, Myb, and miR-200c it is likely that these links apply to human cells. The individual functions of NKX2-1 [5-7,49], miR-200c [30,50,51], MYB [39,52], and NFIB [42] in lung development and/or lung cancer have been widely documented. Thus, our future analyses are aimed at characterizing these novel regulatory links between NKX2-1:miR-200c: NFIB, or MYB in propagating fluctuations in the levels of NKX2-1 in human lung tumors.

## Additional files

Additional file 1:
**Additional Methods.** Detailed ChIP, and miRNA and gene expression array experiments. Additional file 2: Table S1. mmu-miR-200c and mmu-miR-1195 predicted target genes identified in TargetScanMouse 6.2. Additional file 3: Table S2. Gene expression changes induced by down-regulation of Nkx2-1 in MLE15 cells. Additional file 4: Table S3. Gene ontology analysis of predicted miR-200c targets down-regulated in Nkx2-1 knock-down cells.

## References

[1] Boggaram V (2009). Thyroid transcription factor-1 (TTF-1/Nkx2.1/TITF1) gene regulation in the lung. Clin Sci (Lond).

[2] Kimura S, Hara Y, Pineau T, Fernandez-Salguero P, Fox CH, Ward JM (1996). The T/ebp null mouse: thyroid-specific enhancer-binding protein is essential for the organogenesis of the thyroid, lung, ventral forebrain, and pituitary. Genes Dev.

[3] Kimura S, Ward JM, Minoo P (1999). Thyroid-specific enhancer-binding protein/thyroid transcription factor 1 is not required for the initial specification of the thyroid and lung primordia. Biochimie.

[4] Galambos C, Levy H, Cannon CL, Vargas SO, Reid LM, Cleveland R (2010). Pulmonary pathology in thyroid transcription factor-1 deficiency syndrome. Am J Respir Crit Care Med.

[5] Kwei KA, Kim YH, Girard L, Kao J, Pacyna-Gengelbach M, Salari K (2008). Genomic profiling identifies TITF1 as a lineage-specific oncogene amplified in lung cancer. Oncogene.

[6] Mu D (2013). The complexity of thyroid transcription factor 1 with both pro- and anti-oncogenic activities. J Biol Chem.

[7] Perner S, Wagner PL, Soltermann A, LaFargue C, Tischler V, Weir BA (2009). TTF1 expression in non-small cell lung carcinoma: association with TTF1 gene amplification and improved survival. J Pathol.

[8] Winslow MM, Dayton TL, Verhaak RG, Kim-Kiselak C, Snyder EL, Feldser DM (2011). Suppression of lung adenocarcinoma progression by Nkx2-1. Nature.

[9] Kolla V, Gonzales LW, Gonzales J, Wang P, Angampalli S, Feinstein SI (2007). Thyroid transcription factor in differentiating type II cells: regulation, isoforms, and target genes. Am J Respir Cell Mol Biol.

[10] Maeda Y, Tsuchiya T, Hao H, Tompkins DH, Xu Y, Mucenski ML (2012). Kras(G12D) and Nkx2-1 haploinsufficiency induce mucinous adenocarcinoma of the lung. J Clin Invest.

[11] Snyder EL, Watanabe H, Magendantz M, Hoersch S, Chen TA, Wang DG (2013). Nkx2-1 represses a latent gastric differentiation program in lung adenocarcinoma. Mol Cell.

[12] Tagne JB, Gupta S, Gower AC, Shen SS, Varma S, Lakshminarayanan M (2012). Genome-wide analyses of Nkx2-1 binding to transcriptional target genes uncover novel regulatory patterns conserved in lung development and tumors. PLoS One.

[13] Watanabe H, Francis JM, Woo MS, Etemad B, Lin W, Fries DF (2013). Integrated cistromic and expression analysis of amplified NKX2-1 in lung adenocarcinoma identifies LMO3 as a functional transcriptional target. Genes Dev.

[14] Sessa R, Hata A (2013). Role of microRNAs in lung development and pulmonary diseases. Pulm Circ.

[15] Dong J, Jiang G, Asmann YW, Tomaszek S, Jen J, Kislinger T (2010). MicroRNA networks in mouse lung organogenesis. PLoS One.

[16] Pandit KV, Milosevic J, Kaminski N (2011). MicroRNAs in idiopathic pulmonary fibrosis. Transl Res.

[17] Mujahid S, Logvinenko T, Volpe MV (2013). Nielsen HC: miRNA regulated pathways in late stage murine lung development. BMC Dev Biol.

[18] Lu Y, Thomson JM, Wong HY, Hammond SM, Hogan BL (2007). Transgenic over-expression of the microRNA miR-17-92 cluster promotes proliferation and inhibits differentiation of lung epithelial progenitor cells. Dev Biol.

[19] Ventura A, Young AG, Winslow MM, Lintault L, Meissner A, Erkeland SJ (2008). Targeted deletion reveals essential and overlapping functions of the miR-17 through 92 family of miRNA clusters. Cell.

[20] Tian Y, Zhang Y, Hurd L, Hannenhalli S, Liu F, Lu MM (2011). Regulation of lung endoderm progenitor cell behavior by miR302/367. Development.

[21] Wikenheiser KA, Vorbroker DK, Rice WR, Clark JC, Bachurski CJ, Oie HK (1993). Production of immortalized distal respiratory epithelial cell lines from surfactant protein C/simian virus 40 large tumor antigen transgenic mice. Proc Natl Acad Sci U S A.

[22] Cao Y, Vo T, Millien G, Tagne JB, Kotton D, Mason RJ (2010). Epigenetic mechanisms modulate thyroid transcription factor 1-mediated transcription of the surfactant protein B gene. J Biol Chem.

[23] DeFelice M, Silberschmidt D, DiLauro R, Xu Y, Wert SE, Weaver TE (2003). TTF-1 phosphorylation is required for peripheral lung morphogenesis, perinatal survival, and tissue-specific gene expression. J Biol Chem.

[24] Benjamini Y, Hochberg Y (1995). Controlling the false discovery rate: a practical and powerful approach to multiple testing. J R Stat Soc Ser B Methodol.

[25] Varma S, Cao Y, Tagne JB, Lakshminarayanan M, Li J, Friedman TB (2012). The transcription factors grainyhead-like 2 and NK2-homeobox 1 form a regulatory loop that coordinates lung epithelial cell morphogenesis and differentiation. J Biol Chem.

[26] Irizarry RA, Hobbs B, Collin F, Beazer-Barclay YD, Antonellis KJ, Scherf U (2003). Exploration, normalization, and summaries of high density oligonucleotide array probe level data. Biostatistics.

[27] Friedman RC, Farh KK, Burge CB, Bartel DP (2009). Most mammalian mRNAs are conserved targets of microRNAs. Genome Res.

[28] Chang JT, Nevins JR (2006). GATHER: a systems approach to interpreting genomic signatures. Bioinformatics.

[29] Karolchik D, Hinrichs AS, Kent WJ (2012). The UCSC genome browser. Curr Protoc Bioinformatics.

[30] Tejero R, Navarro A, Campayo M, Vinolas N, Marrades RM, Cordeiro A (2014). miR-141 and miR-200c as markers of overall survival in early stage non-small cell lung cancer adenocarcinoma. PLoS One.

[31] Zhang B, Zhang Z, Xia S, Xing C, Ci X, Li X (2013). KLF5 activates microRNA 200 transcription to maintain epithelial characteristics and prevent induced epithelial-mesenchymal transition in epithelial cells. Mol Cell Biol.

[32] Marson A, Levine SS, Cole MF, Frampton GM, Brambrink T, Johnstone S (2008). Connecting microRNA genes to the core transcriptional regulatory circuitry of embryonic stem cells. Cell.

[33] Huang HN, Chen SY, Hwang SM, Yu CC, Su MW, Mai W (2014). miR-200c and GATA binding protein 4 regulate human embryonic stem cell renewal and differentiation. Stem Cell Res.

[34] Howe EN, Cochrane DR, Cittelly DM, Richer JK (2012). miR-200c targets a NF-kappaB up-regulated TrkB/NTF3 autocrine signaling loop to enhance anoikis sensitivity in triple negative breast cancer. PLoS One.

[35] Wellner U, Schubert J, Burk UC, Schmalhofer O, Zhu F, Sonntag A (2009). The EMT-activator ZEB1 promotes tumorigenicity by repressing stemness-inhibiting microRNAs. Nat Cell Biol.

[36] Griffiths-Jones S, Saini HK, van Dongen S (2008). miRBase: tools for microRNA genomics. Nucleic Acids Res.

[37] Wang Y, Weng T, Gou D, Chen Z, Chintagari NR, Liu L (2007). Identification of rat lung-specific microRNAs by micoRNA microarray: valuable discoveries for the facilitation of lung research. BMC Genomics.

[38] Grunder A, Ebel TT, Mallo M, Schwarzkopf G, Shimizu T, Sippel AE (2002). Nuclear factor I-B (Nfib) deficient mice have severe lung hypoplasia. Mech Dev.

[39] Griffin CA, Baylin SB (1985). Expression of the c-myb oncogene in human small cell lung carcinoma. Cancer Res.

[40] Tao T, Liu D, Liu C, Xu B, Chen S, Yin Y (2014). Autoregulatory feedback loop of EZH2/miR-200c/E2F3 as a driving force for prostate cancer development. Biochim Biophys Acta.

[41] Kopp F, Wagner E, Roidl A (2014). The proto-oncogene KRAS is targeted by miR-200c. Oncotarget.

[42] Dooley AL, Winslow MM, Chiang DY, Banerji S, Stransky N, Dayton TL (2011). Nuclear factor I/B is an oncogene in small cell lung cancer. Genes Dev.

[43] Cesi V, Casciati A, Sesti F, Tanno B, Calabretta B, Raschella G (2011). TGFbeta-induced c-Myb affects the expression of EMT-associated genes and promotes invasion of ER+ breast cancer cells. Cell Cycle.

[44] Siebzehnrubl FA, Silver DJ, Tugertimur B, Deleyrolle LP, Siebzehnrubl D, Sarkisian MR (2013). The ZEB1 pathway links glioblastoma initiation, invasion and chemoresistance. EMBO Mol Med.

[45] Yuan Z, Sun X, Liu H, Xie J (2011). MicroRNA genes derived from repetitive elements and expanded by segmental duplication events in mammalian genomes. PLoS One.

[46] Kesavan K, Lobel-Rice K, Sun W, Lapadat R, Webb S, Johnson GL (2004). MEKK2 regulates the coordinate activation of ERK5 and JNK in response to FGF-2 in fibroblasts. J Cell Physiol.

[47] Thum T, Erpenbeck VJ, Moeller J, Hohlfeld JM, Krug N, Borlak J (2006). Expression of xenobiotic metabolizing enzymes in different lung compartments of smokers and nonsmokers. Environ Health Perspect.

[48] Wang SL, He XY, Hong JY (2005). Human cytochrome p450 2s1: lack of activity in the metabolic activation of several cigarette smoke carcinogens and in the metabolism of nicotine. Drug Metab Dispos.

[49] Yamaguchi T, Hosono Y, Yanagisawa K, Takahashi T (2013). NKX2-1/TTF-1: an enigmatic oncogene that functions as a double-edged sword for cancer cell survival and progression. Cancer Cell.

[50] Ceppi P, Mudduluru G, Kumarswamy R, Rapa I, Scagliotti GV, Papotti M (2010). Loss of miR-200c expression induces an aggressive, invasive, and chemoresistant phenotype in non-small cell lung cancer. Mol Cancer Res.

[51] Feng X, Wang Z, Fillmore R, Xi Y (2014). MiR-200, a new star miRNA in human cancer. Cancer Lett.

[52] Yang ZH, Zheng R, Gao Y, Zhang Q, Zhang H (2014). Abnormal gene expression and gene fusion in lung adenocarcinoma with high-throughput RNA sequencing. Cancer Gene Ther.

