# Supplementary material for: Transcription factor and microRNA interactions in lung cells: an inhibitory link between NK2 homeobox 1, miR-200c and the developmental and oncogenic factors Nfib and Myb
Source: Respir Res. 2015 Feb 13;16(1):22. doi: 10.1186/s12931-015-0186-6 (PMC4335692; doi:10.1186/s12931-015-0186-6)
Supplement: Additional file 1: — Additional Methods. Detailed ChIP, and miRNA and gene expression array experiments. [file 12931_2015_186_MOESM1_ESM.docx]

**Supplementary Materials**

*miRNA Array experiments*

All procedures were performed at Boston University Microarray Resource Facility following the protocols described in FlashTag^TM^Biotin RNA Labeling Kit for Affymetrix miRNA Arrays protocol (Genisphere Inc., Hatfield, PA). Briefly, total RNA was isolated from three independent Nkx2-1-shRNA and control transduced MLE15 cell lines [[1](#_ENREF_1)] and enriched for low molecular weight RNA using QIAGEN’s miRNeasy kit (Qiagen, Valencia, CA). RNA was labeled following the FlashTag kit (Genisphere Inc., Hatfield, PA) protocol. Labeled RNA was hybridized to miRNA Galaxy arrays (Affymetrix, Santa Clara, CA) for 16 hours in GeneChip Hybridization oven 640 at 48^o^C with rotation (60 rpm). Hybridized samples were washed and stained using Affymetrix fluidics station 450. Microarrays were scanned using Affymetrix GeneArray Scanner 3000 7G Plus (Affymetrix, Santa Clara, CA). Affymetrix miRNA QC Tool software version 1.0.33.0 was used for background subtraction, detection p-value calculation and array normalization. A two-sample t-test was performed to identify differentially regulated miRNA expression in Nkx2-1 knockdown cells and a false discovery rate (FDR) correction [[2](#_ENREF_2), [3](#_ENREF_3)]. Genes with a *p* ≤ 0.05 and an FDR adjusted *p* value < 0.2 were considered to be differentially expressed.

*Gene Expression Array experiments*.

All procedures were performed at Boston University Microarray Resource Facility as described in GeneChip® Whole Transcript (WT) Sense Target Labeling Assay Manual (Affymetrix, Santa Clara, CA). Total RNA was isolated using QIAGEN’s RNeasy kit (Qiagen, Valencia, CA) RNA (300 ng) was reverse transcribed using Whole Transcript cDNA Synthesis kit (Affymetrix, Santa Clara, CA). The cDNA was used as a template for in vitro transcription using Whole Transcript cDNA Amplification Kit (Affymetrix, Santa Clara, CA). The antisense cRNA was purified using GeneChip Sample Cleanup Module (Affymetrix, Santa Clara, CA), and used as a template for reverse transcription (Whole Transcript cDNA Synthesis kit, Affymetrix, Santa Clara, CA) to produce single-stranded DNA in the sense orientation. The labeled fragmented DNA was hybridized to the Gene Arrays 1.0ST for 16-18 hours in GeneChip Hybridization oven 640 at 45°C with rotation (60 rpm). The hybridized samples were washed and stained using Affymetrix fluidics station 450. Microarrays were scanned using Affymetrix GeneArray Scanner 3000 7G Plus (Affymetrix, Santa Clara, CA). The resulting CEL files were summarized using Affymetrix Expression Console (current version 1.1). RMA (Robust Multi-Array Analysis) algorithm [[4](#_ENREF_4)] was used to generate gene-level data. A two-sample t-test was performed to identify differentially regulated mRNA expression in Nkx2-1 knockdown cells followed by a adjustment correction for multiple comparisons [2]. Genes with an adjusted p ≤ 0.0005 were considered significant. Both mRNA and microRNA expression data are deposited in the Gene Expression Omnibus (GEO) GSE47055.

*Chromatin immunoprecipitation assays*

About 1 x 10^6^ cells from the three independent Nkx2-1-shRNA and control transduced MLE15 cell lines [12] were fixed in 1% formaldehyde in 1× phosphate-buffered saline at room temperature for 10 min. After washing with 1× phosphate-buffered saline, cells were suspended in lysis buffer (1% SDS, 10 mm EDTA, 50 mm Tris-HCl, pH 8, and 1× protease inhibitor mixture Pierce (Thermo Scientific)) and sonicated by using a Branson 450 dismembrator to achieve a chromatin fragment size of 500–1000 bp. We immunoprecipitated chromatin fragments by incubating the samples (equal amount of DNA) at 4 °C overnight with 10 μl of Nkx2-1 antibody (07-601, Upstate (Millipore)) antibody or the corresponding IgG control (Santa Cruz Biotechnology) to determine nonspecific binding. Pre-absorbed protein A/G beads (Santa Cruz Biotechnology) were used to immunoprecipitate chromatin-antibody complexes. Equal volumes of immunoprecipitated DNA solution and 10% of the input DNA fragments were amplified by qPCR using a custom designed TaqMan (Applied Biosystems) assay within -1kb relative to the first nucleotide of the pre-miRNA sequence indicated in the UCSC Genome Browser version (miR-1195, mm10 chr17:70860551-70861600; miR-200c, mm10 chr6:124718366-124719390) and quantified in a StepOnePlus using TaqMan Master Mix (Applied Biosystems). Data were normalized to IgG control and expressed as percentage of the input.

**Table Legends**

**Table S1 –** mmu-miR-200c and mmu-mir-1195 predicted target genes identified in TargetScanMouse 6.2 (total context score ≤ -0.01).

**Table S2 -** Gene expression changes induced by down-regulation of Nkx2-1 in MLE15 cells. Adjusted p value < 0.0005.

**Table S3 -** Gene ontology analysis of predicted miR-200c targets that are down-regulated in Nkx2-1 knock-down cells**.** Bayes Factor >3.00, more than 5 genes per GO group.

**References in the Supplementary Methods**

1. Tagne JB, Gupta S, Gower AC, Shen SS, Varma S, Lakshminarayanan M, Cao Y, Spira A, Volkert TL, Ramirez MI: **Genome-wide analyses of Nkx2-1 binding to transcriptional target genes uncover novel regulatory patterns conserved in lung development and tumors**. *PLoS One* 2012, **7**(1):e29907.

2. Benjamini Y, Drai D, Elmer G, Kafkafi N, Golani I: **Controlling the false discovery rate in behavior genetics research**. *Behav Brain Res* 2001, **125**(1-2):279-284.

3. Benjamini Y, Hochberg Y: **Controlling the False Discovery Rate: A Practical and Powerful Approach to Multiple Testing**. *Journal of the Royal Statistical Society Series B (Methodological)* 1995, **57**(1):289-300.

4. Irizarry RA, Hobbs B, Collin F, Beazer-Barclay YD, Antonellis KJ, Scherf U, Speed TP: **Exploration, normalization, and summaries of high density oligonucleotide array probe level data**. *Biostatistics* 2003, **4**(2):249-264.
