# Supplementary material for: Transcription factor and microRNA interactions in lung cells: an inhibitory link between NK2 homeobox 1, miR-200c and the developmental and oncogenic factors Nfib and Myb
Source: Respir Res. 2015 Feb 13;16(1):22. doi: 10.1186/s12931-015-0186-6 (PMC4335692; doi:10.1186/s12931-015-0186-6)
Supplement: Additional file 2: Table S1. — mmu-miR-200c and mmu-miR-1195 predicted target genes identified in TargetScanMouse 6.2. [file 12931_2015_186_MOESM2_ESM.docx]

| *Table S1. mmu-miR-200c and mmu-mir-1195 predicted target genes identified in TargetScanMouse 6.2 (total context score ≤ -0.01).* | | | | |
| --- | --- | --- | --- | --- |
| miR-200c Target gene | Representative transcript | Conserved sites | Poorly conserved sites | Aggregate PCT |
|  |  | total | total |  |
| Zeb2 | NM_015753 | 6 | 1 | > 0.99 |
| Zeb1 | NM_011546 | 5 | 0 | > 0.99 |
| Qk | NM_001159517 | 3 | 1 | > 0.99 |
| Eif2c2 | NM_153178 | 2 | 0 | > 0.99 |
| C030046E11Rik | NM_001081319 | 2 | 1 | > 0.99 |
| Sec23a | NM_009147 | 3 | 0 | > 0.99 |
| Hnrnpd | NM_001077265 | 2 | 1 | > 0.99 |
| Msn | NM_010833 | 2 | 0 | 0.99 |
| Hipk3 | NM_001145824 | 3 | 0 | 0.98 |
| Dlc1 | NM_001194940 | 2 | 0 | 0.98 |
| Wdr82 | NM_029896 | 2 | 0 | 0.98 |
| Dpy19l1 | NM_172920 | 2 | 2 | 0.98 |
| Jhdm1d | NM_001033430 | 2 | 1 | 0.98 |
| Slc1a2 | NM_001077514 | 3 | 0 | 0.98 |
| Wipf1 | NM_153138 | 2 | 0 | 0.98 |
| Ppm1f | NM_176833 | 3 | 0 | 0.98 |
| Arih1 | NM_019927 | 2 | 0 | 0.98 |
| Ywhag | NM_018871 | 2 | 1 | 0.97 |
| Ranbp10 | NM_145824 | 2 | 0 | 0.97 |
| Nfia | NM_001122952 | 2 | 2 | 0.97 |
| Errfi1 | NM_133753 | 2 | 0 | 0.96 |
| Eps8 | NM_007945 | 2 | 0 | 0.96 |
| Fbxw7 | NM_001177773 | 2 | 0 | 0.95 |
| Pkd1 | NM_013630 | 2 | 1 | 0.95 |
| Reep1 | NM_178608 | 2 | 1 | 0.95 |
| Ptpn14 | NM_008976 | 2 | 1 | 0.95 |
| Synj1 | NM_001045515 | 2 | 1 | 0.95 |
| Ypel2 | NM_001005341 | 2 | 1 | 0.95 |
| Rap1b | NM_024457 | 2 | 0 | 0.95 |
| Slit2 | NM_178804 | 2 | 0 | 0.94 |
| Zfp532 | NM_207255 | 2 | 0 | 0.94 |
| Rasa2 | NM_053268 | 2 | 0 | 0.94 |
| Cecr2 | NM_001128151 | 2 | 0 | 0.94 |
| Tmem41b | NM_153525 | 1 | 1 | 0.94 |
| E130309F12Rik | NM_178756 | 2 | 0 | 0.93 |
| Myo9a | NM_173018 | 1 | 1 | 0.93 |
| Slc14a1 | NM_001171010 | 1 | 1 | 0.93 |
| 2700081O15Rik | NM_175381 | 2 | 0 | 0.93 |
| Tsga14 | NM_031998 | 2 | 0 | 0.93 |
| Rbfox2 | NM_001110827 | 2 | 0 | 0.93 |
| Atxn1 | NM_001199304 | 2 | 0 | 0.93 |
| Nova1 | NM_021361 | 2 | 1 | 0.92 |
| Tbc1d12 | NM_145952 | 1 | 1 | 0.92 |
| Bap1 | NM_027088 | 2 | 0 | 0.92 |
| Fignl2 | NM_001214911 | 1 | 1 | 0.92 |
| Evi5 | NM_007964 | 1 | 0 | 0.92 |
| Tsc22d2 | NM_001081229 | 1 | 1 | 0.92 |
| Adamts3 | NM_001081401 | 1 | 0 | 0.92 |
| Tln2 | NM_001081242 | 1 | 0 | 0.92 |
| Mprip | NM_012027 | 1 | 0 | 0.92 |
| Sh3pxd2a | NM_001164717 | 1 | 0 | 0.92 |
| Zcchc24 | NM_001101433 | 1 | 0 | 0.92 |
| 41706 | NM_027920 | 1 | 0 | 0.92 |
| Aff1 | NM_001080798 | 1 | 0 | 0.92 |
| Mtss1l | NM_198625 | 1 | 1 | 0.92 |
| Npc1 | NM_008720 | 1 | 0 | 0.92 |
| Marcks | NM_008538 | 1 | 0 | 0.92 |
| Cbx5 | NM_001076789 | 1 | 1 | 0.92 |
| Mmd | NM_026178 | 1 | 1 | 0.91 |
| Pkia | NM_008862 | 1 | 0 | 0.91 |
| Trim33 | NM_001079830 | 1 | 0 | 0.91 |
| Mapre1 | NM_007896 | 1 | 0 | 0.91 |
| Git2 | NM_001077359 | 1 | 0 | 0.91 |
| Inpp4a | NM_030266 | 1 | 0 | 0.91 |
| Vash2 | NM_144879 | 1 | 1 | 0.91 |
| Zfp217 | NM_001033299 | 1 | 0 | 0.91 |
| Ppp2r2c | NM_172994 | 1 | 0 | 0.91 |
| Tmeff2 | NM_019790 | 1 | 1 | 0.91 |
| Ubn2 | NM_177185 | 1 | 1 | 0.91 |
| Slc6a1 | NM_178703 | 1 | 0 | 0.91 |
| Pvrl4 | NM_001122680 | 1 | 0 | 0.91 |
| Clasp2 | NM_001081960 | 1 | 0 | 0.91 |
| Ptpn21 | NM_001146199 | 1 | 0 | 0.91 |
| Cops8 | NM_133805 | 1 | 0 | 0.91 |
| Rbfox3 | NM_001024931 | 1 | 0 | 0.91 |
| Pdik1l | NM_001163794 | 1 | 0 | 0.91 |
| Rtf1 | NM_030112 | 1 | 0 | 0.91 |
| Prr11 | NM_175563 | 1 | 0 | 0.91 |
| Ets1 | NM_001038642 | 2 | 1 | 0.91 |
| Fez2 | NM_199448 | 1 | 0 | 0.91 |
| Iqsec1 | NM_001134384 | 1 | 0 | 0.91 |
| Psip1 | NM_133948 | 1 | 0 | 0.91 |
| Sfxn1 | NM_027324 | 1 | 0 | 0.91 |
| Syde1 | NM_027875 | 1 | 0 | 0.91 |
| Nr2c2 | NM_011630 | 2 | 1 | 0.91 |
| Dennd5b | NM_177192 | 1 | 1 | 0.91 |
| Amfr | NM_011787 | 1 | 0 | 0.91 |
| Srsf1 | NM_001078167 | 1 | 4 | 0.9 |
| Vldlr | NM_001161420 | 1 | 1 | 0.9 |
| Gabpa | NM_008065 | 1 | 1 | 0.9 |
| Clic4 | NM_013885 | 1 | 0 | 0.9 |
| Rnd3 | NM_028810 | 2 | 0 | 0.9 |
| Spag9 | NM_001025428 | 2 | 0 | 0.9 |
| Ick | NM_001163780 | 1 | 0 | 0.9 |
| Greb1l | NM_001083628 | 1 | 0 | 0.9 |
| Lrp1 | NM_008512 | 1 | 0 | 0.9 |
| Six1 | NM_009189 | 1 | 0 | 0.9 |
| Srgap1 | NM_001081037 | 1 | 0 | 0.9 |
| Asap1 | NM_010026 | 2 | 0 | 0.9 |
| Xkr8 | NM_201368 | 1 | 0 | 0.9 |
| Slc6a11 | NM_172890 | 1 | 0 | 0.9 |
| Gpatch8 | NM_001159492 | 1 | 0 | 0.9 |
| Zfyve20 | NM_030081 | 1 | 1 | 0.9 |
| Gli3 | NM_008130 | 1 | 0 | 0.9 |
| E2f3 | NM_010093 | 2 | 0 | 0.9 |
| Fhod1 | NM_177699 | 1 | 1 | 0.9 |
| Prdm16 | NM_001177995 | 1 | 0 | 0.9 |
| Pard6b | NM_021409 | 1 | 0 | 0.89 |
| Med13 | NM_001080931 | 2 | 2 | 0.89 |
| Clasp1 | NM_001081276 | 1 | 0 | 0.89 |
| Eif4e2 | NM_023314 | 1 | 0 | 0.89 |
| Tubb5 | NM_011655 | 1 | 0 | 0.89 |
| Map3k13 | NM_172821 | 1 | 0 | 0.89 |
| Slc23a2 | NM_018824 | 1 | 3 | 0.89 |
| Ndst1 | NM_008306 | 1 | 0 | 0.89 |
| Phactr3 | NM_001007154 | 1 | 1 | 0.89 |
| Dnajb5 | NM_019874 | 1 | 0 | 0.89 |
| Depdc1b | NM_178683 | 1 | 0 | 0.89 |
| Srpk2 | NM_009274 | 1 | 2 | 0.89 |
| Hlf | NM_172563 | 1 | 1 | 0.89 |
| Rgl1 | NM_016846 | 1 | 0 | 0.89 |
| Ube2i | NM_001177609 | 1 | 0 | 0.89 |
| Anln | NM_028390 | 1 | 0 | 0.89 |
| Slc30a4 | NM_011774 | 2 | 1 | 0.89 |
| Nab1 | NM_008667 | 1 | 1 | 0.88 |
| Coro1c | NM_011779 | 1 | 0 | 0.88 |
| Rhot1 | NM_001163354 | 1 | 0 | 0.88 |
| Dach1 | NM_001038610 | 1 | 0 | 0.88 |
| Arhgef17 | NM_001081116 | 1 | 0 | 0.88 |
| Fbxo33 | NM_001033156 | 1 | 0 | 0.88 |
| Mga | NM_001164274 | 1 | 1 | 0.88 |
| Mtap2 | NM_001039934 | 1 | 2 | 0.88 |
| Efnb2 | NM_010111 | 1 | 0 | 0.88 |
| Ncoa2 | NM_001077695 | 2 | 0 | 0.88 |
| Ccnj | NM_172839 | 1 | 1 | 0.88 |
| Pppde1 | NM_024282 | 1 | 0 | 0.88 |
| Tead1 | NM_001166584 | 1 | 0 | 0.88 |
| Flt1 | NM_010228 | 1 | 1 | 0.88 |
| Mmgt1 | NM_146234 | 1 | 0 | 0.88 |
| Rap2c | NM_172413 | 1 | 1 | 0.88 |
| Dnajc1 | NM_007869 | 1 | 0 | 0.88 |
| Ank3 | NM_009670 | 1 | 1 | 0.88 |
| Glis2 | NM_031184 | 1 | 0 | 0.88 |
| Jun | NM_010591 | 1 | 0 | 0.88 |
| Nova2 | NM_001029877 | 2 | 1 | 0.88 |
| Cdk17 | NM_146239 | 1 | 0 | 0.88 |
| Foxk1 | NM_199068 | 1 | 0 | 0.87 |
| Phf21a | NM_001109690 | 1 | 0 | 0.87 |
| Crybg3 | NM_174848 | 1 | 3 | 0.87 |
| Mecp2 | NM_001081979 | 1 | 1 | 0.87 |
| Slc35a2 | NM_078484 | 1 | 1 | 0.87 |
| Dtna | NM_207650 | 1 | 0 | 0.87 |
| Tmem120b | NM_001039723 | 1 | 0 | 0.87 |
| Slc39a14 | NM_001135151 | 1 | 0 | 0.87 |
| Gata2 | NM_008090 | 1 | 0 | 0.87 |
| Slitrk1 | NM_199065 | 1 | 0 | 0.87 |
| Golga1 | NM_029793 | 1 | 1 | 0.87 |
| AI593442 | NM_178906 | 1 | 2 | 0.87 |
| Bcl11b | NM_001079883 | 2 | 0 | 0.87 |
| Hspa13 | NM_030201 | 1 | 1 | 0.87 |
| Uhrf1bp1 | NM_001080769 | 1 | 0 | 0.87 |
| Astn1 | NM_001205204 | 1 | 0 | 0.87 |
| 4931406P16Rik | NM_172741 | 1 | 0 | 0.87 |
| Ier5 | NM_010500 | 1 | 0 | 0.87 |
| Scd2 | NM_009128 | 1 | 1 | 0.86 |
| 2310014H01Rik | NM_001146710 | 1 | 0 | 0.86 |
| Scn3b | NM_001083917 | 1 | 0 | 0.86 |
| Glcci1 | NM_133236 | 1 | 0 | 0.86 |
| Egln1 | NM_053207 | 1 | 0 | 0.86 |
| Agap1 | NM_001037136 | 1 | 1 | 0.86 |
| Irs4 | NM_010572 | 1 | 1 | 0.86 |
| Pi4kb | NM_175356 | 1 | 0 | 0.86 |
| Akap2 | NM_001035532 | 2 | 0 | 0.86 |
| Eif5b | NM_198303 | 1 | 1 | 0.86 |
| Tmem170b | NM_001163572 | 2 | 0 | 0.86 |
| Thap1 | NM_199042 | 1 | 1 | 0.86 |
| Fam63b | NM_172772 | 1 | 0 | 0.86 |
| Gatsl2 | NM_030719 | 1 | 0 | 0.86 |
| Jazf1 | NM_001168277 | 1 | 0 | 0.86 |
| Pcsk2 | NM_008792 | 1 | 0 | 0.86 |
| Plcl1 | NM_001114663 | 1 | 1 | 0.86 |
| Sesn1 | NM_001013370 | 1 | 0 | 0.85 |
| Gnai3 | NM_010306 | 1 | 1 | 0.85 |
| Tnfrsf11b | NM_008764 | 1 | 0 | 0.85 |
| Dnajc5 | NM_016775 | 1 | 1 | 0.85 |
| Hspa9 | NM_010481 | 1 | 0 | 0.85 |
| Chd1 | NM_007690 | 1 | 0 | 0.85 |
| St3gal2 | NM_009179 | 1 | 1 | 0.85 |
| Palm2 | NM_172868 | 1 | 0 | 0.85 |
| Lcorl | NM_001163073 | 2 | 0 | 0.85 |
| Pcnx | NM_018814 | 1 | 0 | 0.85 |
| Trp73 | NM_001126330 | 1 | 0 | 0.85 |
| Eif2s1 | NM_026114 | 1 | 0 | 0.85 |
| Acp1 | NM_001110239 | 1 | 0 | 0.85 |
| Ppp4r2 | NM_182939 | 2 | 0 | 0.85 |
| Lfng | NM_008494 | 1 | 0 | 0.85 |
| Nog | NM_008711 | 1 | 0 | 0.85 |
| Asf1a | NM_025541 | 1 | 2 | 0.85 |
| Nptx1 | NM_008730 | 1 | 0 | 0.84 |
| Gpm6a | NM_153581 | 2 | 1 | 0.84 |
| Phldb1 | NM_153537 | 1 | 0 | 0.84 |
| Fign | NM_021716 | 1 | 0 | 0.84 |
| Usp6nl | NM_001080548 | 1 | 0 | 0.84 |
| Srf | NM_020493 | 2 | 0 | 0.84 |
| Pcmtd1 | NM_183028 | 1 | 2 | 0.84 |
| Casz1 | NM_001159344 | 1 | 0 | 0.84 |
| Tbc1d22b | NM_198647 | 1 | 0 | 0.84 |
| Ankrd28 | NM_001024604 | 1 | 0 | 0.84 |
| Rps6kb1 | NM_001114334 | 1 | 2 | 0.84 |
| Rab11fip2 | NM_001033172 | 1 | 2 | 0.84 |
| Zfpm2 | NM_011766 | 2 | 0 | 0.84 |
| Kank1 | NM_181404 | 1 | 1 | 0.83 |
| Nfib | NM_001113209 | 2 | 1 | 0.83 |
| Taok1 | NM_144825 | 1 | 1 | 0.83 |
| C230081A13Rik | NM_172924 | 1 | 1 | 0.83 |
| Serinc1 | NM_019760 | 1 | 0 | 0.83 |
| Nr5a2 | NM_001159769 | 1 | 1 | 0.83 |
| Ormdl3 | NM_025661 | 1 | 0 | 0.83 |
| 1810013L24Rik | NM_001081400 | 1 | 1 | 0.83 |
| Ckap4 | NM_175451 | 1 | 0 | 0.83 |
| Map4k4 | NM_008696 | 1 | 0 | 0.83 |
| Ncs1 | NM_019681 | 1 | 0 | 0.83 |
| Usp25 | NM_013918 | 1 | 0 | 0.83 |
| Zfp292 | NM_013889 | 2 | 1 | 0.83 |
| Csrnp3 | NM_153409 | 1 | 1 | 0.82 |
| Slc16a2 | NM_009197 | 1 | 0 | 0.82 |
| Kras | NM_021284 | 1 | 0 | 0.82 |
| Lass6 | NM_172856 | 1 | 1 | 0.82 |
| Dnmt3a | NM_007872 | 1 | 0 | 0.82 |
| Golim4 | NM_175193 | 1 | 0 | 0.82 |
| Vezf1 | NM_016686 | 1 | 1 | 0.82 |
| Ccnyl1 | NM_001097644 | 1 | 0 | 0.82 |
| Ranbp9 | NM_019930 | 1 | 0 | 0.82 |
| Rapgef2 | NM_001099624 | 1 | 0 | 0.82 |
| Negr1 | NM_001039094 | 2 | 1 | 0.82 |
| Smarcad1 | NM_007958 | 1 | 0 | 0.81 |
| Fscn1 | NM_007984 | 1 | 0 | 0.81 |
| Erc1 | NM_053204 | 1 | 1 | 0.81 |
| Pik3ca | NM_008839 | 2 | 0 | 0.81 |
| Aff3 | NM_010678 | 1 | 0 | 0.81 |
| Ipo8 | NM_001081113 | 1 | 1 | 0.81 |
| Sik1 | NM_010831 | 1 | 0 | 0.81 |
| Fbxo42 | NM_172518 | 1 | 0 | 0.81 |
| Ptbp1 | NM_001077363 | 1 | 0 | 0.81 |
| Pikfyve | NM_011086 | 1 | 0 | 0.81 |
| Six3 | NM_011381 | 1 | 0 | 0.8 |
| Pip4k2b | NM_054051 | 1 | 0 | 0.8 |
| Pard3b | NM_001081050 | 1 | 0 | 0.8 |
| Lamc1 | NM_010683 | 1 | 0 | 0.8 |
| Cdr2 | NM_007672 | 1 | 0 | 0.8 |
| Smurf2 | NM_025481 | 1 | 0 | 0.8 |
| Syvn1 | NM_001164709 | 1 | 0 | 0.8 |
| Hmbox1 | NM_177338 | 1 | 0 | 0.8 |
| Gata4 | NM_008092 | 1 | 0 | 0.8 |
| Foxg1 | NM_001160112 | 1 | 1 | 0.8 |
| Sox1 | NM_009233 | 1 | 0 | 0.8 |
| Gigyf1 | NM_031408 | 1 | 0 | 0.79 |
| Dennd1b | NM_001166501 | 3 | 0 | 0.79 |
| Snph | NM_198214 | 1 | 0 | 0.79 |
| Igsf3 | NM_207205 | 1 | 0 | 0.79 |
| Nufip2 | NM_001024205 | 2 | 0 | 0.79 |
| Lhfp | NM_175386 | 2 | 0 | 0.79 |
| Lrp4 | NM_172668 | 1 | 0 | 0.79 |
| Papd5 | NM_001164497 | 2 | 0 | 0.78 |
| Nudt4 | NM_027722 | 1 | 0 | 0.78 |
| Plxna4 | NM_175750 | 1 | 0 | 0.78 |
| Vat1l | NM_173016 | 1 | 1 | 0.78 |
| Ppp1r9a | NM_181595 | 2 | 1 | 0.78 |
| Osbpl11 | NM_176840 | 1 | 0 | 0.78 |
| Gm949 | NM_001033446 | 1 | 0 | 0.78 |
| Plekhm3 | NM_001039493 | 1 | 0 | 0.77 |
| Mknk1 | NM_021461 | 1 | 0 | 0.77 |
| St6galnac5 | NM_012028 | 1 | 1 | 0.77 |
| Lmtk2 | NM_001081109 | 1 | 0 | 0.77 |
| Abat | NM_001170978 | 1 | 0 | 0.77 |
| Eri1 | NM_026067 | 1 | 0 | 0.77 |
| BC030336 | NM_001164580 | 1 | 2 | 0.77 |
| Arl5a | NM_182994 | 1 | 1 | 0.77 |
| Slc4a4 | NM_001136260 | 1 | 0 | 0.77 |
| Pten | NM_008960 | 1 | 1 | 0.76 |
| Frmd4a | NM_001177843 | 1 | 0 | 0.76 |
| Asxl3 | NM_001167777 | 1 | 1 | 0.76 |
| Ell2 | NM_138953 | 1 | 0 | 0.76 |
| Robo2 | NM_175549 | 1 | 0 | 0.76 |
| Kalrn | NM_177357 | 1 | 2 | 0.76 |
| Sema3f | NM_011349 | 1 | 0 | 0.76 |
| Arhgdia | NM_133796 | 1 | 0 | 0.76 |
| Myt1 | NM_001171615 | 1 | 0 | 0.76 |
| Dennd5a | NM_021494 | 1 | 0 | 0.76 |
| Ints8 | NM_001159595 | 1 | 0 | 0.75 |
| Scrt2 | NM_001160410 | 1 | 0 | 0.75 |
| Gdi2 | NM_008112 | 1 | 0 | 0.75 |
| Lpar1 | NM_010336 | 1 | 1 | 0.75 |
| Plxna2 | NM_008882 | 1 | 0 | 0.75 |
| Suz12 | NM_001163018 | 1 | 0 | 0.75 |
| Zfp711 | NM_177747 | 1 | 1 | 0.75 |
| Slc38a2 | NM_175121 | 1 | 1 | 0.74 |
| Amotl2 | NM_019764 | 1 | 0 | 0.74 |
| Nr3c1 | NM_008173 | 2 | 1 | 0.74 |
| Prkacb | NM_001164198 | 2 | 0 | 0.74 |
| Mtf2 | NM_013827 | 2 | 0 | 0.74 |
| Zfp131 | NM_028245 | 1 | 0 | 0.74 |
| Nup153 | NM_175749 | 2 | 0 | 0.74 |
| Nedd1 | NM_008682 | 1 | 0 | 0.73 |
| Fbxw11 | NM_134015 | 1 | 1 | 0.73 |
| Ube2w | NM_025773 | 1 | 0 | 0.73 |
| Smcr7l | NM_178719 | 1 | 2 | 0.73 |
| Mboat2 | NM_001083341 | 1 | 1 | 0.72 |
| Xiap | NM_009688 | 1 | 0 | 0.72 |
| Kctd10 | NM_001159941 | 1 | 0 | 0.72 |
| Foxf2 | NM_010225 | 1 | 0 | 0.72 |
| Slc38a4 | NM_027052 | 1 | 0 | 0.72 |
| BC037034 | NM_153161 | 1 | 0 | 0.71 |
| Hcn1 | NM_010408 | 1 | 1 | 0.71 |
| Pou6f1 | NM_010127 | 1 | 0 | 0.71 |
| Scd1 | NM_009127 | 1 | 1 | 0.71 |
| Snap25 | NM_011428 | 1 | 1 | 0.71 |
| Ctdspl2 | NM_212450 | 1 | 1 | 0.71 |
| Stard13 | NM_001163493 | 1 | 0 | 0.71 |
| Mmd2 | NM_175217 | 1 | 0 | 0.71 |
| Tmcc1 | NM_177412 | 1 | 1 | 0.71 |
| Chd2 | NM_001081345 | 1 | 1 | 0.7 |
| Scn8a | NM_001077499 | 1 | 0 | 0.7 |
| Fam190a | NM_001164316 | 1 | 0 | 0.7 |
| Nfasc | NM_001160316 | 2 | 0 | 0.7 |
| Nfya | NM_001110832 | 1 | 0 | 0.7 |
| Mex3d | NM_198615 | 1 | 0 | 0.7 |
| Prkcb | NM_008855 | 1 | 0 | 0.69 |
| Mmgt2 | NM_175002 | 1 | 0 | 0.69 |
| Rab37 | NM_001163753 | 1 | 0 | 0.69 |
| Fam120c | NM_198105 | 1 | 0 | 0.69 |
| Tmx4 | NM_029148 | 1 | 0 | 0.69 |
| Spast | NM_001162870 | 1 | 1 | 0.69 |
| Btf3l4 | NM_027453 | 1 | 0 | 0.69 |
| Hs2st1 | NM_011828 | 1 | 1 | 0.69 |
| Acaca | NM_133360 | 1 | 0 | 0.68 |
| 6430573F11Rik | NM_176952 | 1 | 0 | 0.68 |
| Esrrg | NM_011935 | 1 | 2 | 0.68 |
| Fnbp4 | NM_018828 | 1 | 0 | 0.68 |
| Frs2 | NM_177798 | 1 | 0 | 0.67 |
| Dcun1d4 | NM_001190733 | 1 | 0 | 0.67 |
| Apaf1 | NM_001042558 | 1 | 0 | 0.67 |
| Ntf3 | NM_001164034 | 2 | 0 | 0.67 |
| Dek | NM_025900 | 1 | 1 | 0.67 |
| Agfg1 | NM_010472 | 1 | 0 | 0.67 |
| Hnrnpu | NM_016805 | 1 | 0 | 0.67 |
| Ctnnd2 | NM_008729 | 1 | 0 | 0.67 |
| Syt1 | NM_009306 | 1 | 0 | 0.67 |
| Akt2 | NM_001110208 | 1 | 0 | 0.67 |
| Ddx3y | NM_012008 | 1 | 5 | 0.67 |
| Cpd | NM_007754 | 1 | 1 | 0.66 |
| Calu | NM_007594 | 1 | 0 | 0.66 |
| Eif5a2 | NM_177586 | 1 | 0 | 0.66 |
| Nuak1 | NM_001004363 | 1 | 0 | 0.66 |
| Ppp2ca | NM_019411 | 1 | 0 | 0.66 |
| Kif13a | NM_010617 | 1 | 0 | 0.66 |
| Slc10a7 | NM_029736 | 1 | 0 | 0.65 |
| Stx1a | NM_016801 | 1 | 0 | 0.65 |
| Spats2l | NM_001164566 | 1 | 0 | 0.65 |
| Arl5b | NM_029466 | 1 | 0 | 0.65 |
| Cdh11 | NM_009866 | 1 | 0 | 0.64 |
| Vash1 | NM_177354 | 1 | 0 | 0.64 |
| Stx12 | NM_133887 | 1 | 0 | 0.64 |
| Brsk2 | NM_029426 | 1 | 0 | 0.64 |
| Ppap2b | NM_080555 | 1 | 0 | 0.64 |
| Tcfap2a | NM_001122948 | 1 | 1 | 0.64 |
| Cnot4 | NM_001164412 | 1 | 0 | 0.64 |
| Frem2 | NM_172862 | 1 | 1 | 0.64 |
| Nbr1 | NM_008676 | 1 | 1 | 0.64 |
| Fam118b | NM_175411 | 1 | 0 | 0.63 |
| Fat3 | NM_001080814 | 1 | 0 | 0.63 |
| Adipor2 | NM_197985 | 1 | 0 | 0.63 |
| Arhgap19 | NM_001163495 | 1 | 2 | 0.63 |
| Ambra1 | NM_001080754 | 1 | 0 | 0.63 |
| Ikzf2 | NM_011770 | 1 | 0 | 0.63 |
| Cebpd | NM_007679 | 1 | 0 | 0.63 |
| Pcsk5 | NM_001163144 | 1 | 0 | 0.62 |
| Mex3c | NM_001039214 | 1 | 0 | 0.62 |
| Xkr4 | NM_001011874 | 1 | 0 | 0.62 |
| Fubp1 | NM_057172 | 1 | 2 | 0.62 |
| Tbk1 | NM_019786 | 1 | 0 | 0.62 |
| 6330409N04Rik | NM_025697 | 1 | 1 | 0.62 |
| Pds5b | NM_175310 | 1 | 1 | 0.62 |
| Cdk16 | NM_011049 | 1 | 0 | 0.62 |
| Fubp3 | NM_001033389 | 2 | 0 | 0.62 |
| Fam178a | NM_001081225 | 1 | 0 | 0.62 |
| Bach2 | NM_001109661 | 1 | 0 | 0.62 |
| Mll3 | NM_001081383 | 1 | 0 | 0.62 |
| BC024659 | NM_001135577 | 1 | 0 | 0.62 |
| Myb | NM_001198914 | 1 | 1 | 0.62 |
| Hmgb3 | NM_008253 | 1 | 0 | 0.62 |
| Cited2 | NM_010828 | 1 | 0 | 0.62 |
| Arhgap20 | NM_175535 | 1 | 1 | 0.61 |
| Usp31 | NM_001033173 | 1 | 0 | 0.61 |
| Klf12 | NM_010636 | 1 | 0 | 0.61 |
| Mdfic | NM_175088 | 1 | 1 | 0.61 |
| Nek9 | NM_145138 | 1 | 0 | 0.61 |
| Fam60a | NM_019643 | 1 | 0 | 0.61 |
| Ncoa7 | NM_001111267 | 1 | 0 | 0.61 |
| Rnf169 | NM_175388 | 1 | 0 | 0.61 |
| Mex3b | NM_175366 | 1 | 0 | 0.61 |
| Tob1 | NM_009427 | 1 | 0 | 0.61 |
| Baz2b | NM_001001182 | 1 | 0 | 0.61 |
| Pin1 | NM_023371 | 1 | 0 | 0.61 |
| Suv420h1 | NM_001167885 | 1 | 1 | 0.61 |
| Usp27x | NM_019461 | 1 | 0 | 0.61 |
| Mybl1 | NM_008651 | 1 | 0 | 0.6 |
| Slc6a6 | NM_009320 | 1 | 0 | 0.6 |
| Prkar2a | NM_008924 | 1 | 1 | 0.6 |
| Map3k1 | NM_011945 | 1 | 0 | 0.6 |
| Reln | NM_011261 | 1 | 1 | 0.6 |
| Ywhab | NM_018753 | 1 | 0 | 0.6 |
| Eif2b5 | NM_172265 | 1 | 0 | 0.59 |
| Sgip1 | NM_144906 | 1 | 0 | 0.59 |
| Rev1 | NM_019570 | 1 | 0 | 0.59 |
| Bcl2 | NM_009741 | 1 | 0 | 0.59 |
| 1110002B05Rik | NM_134054 | 1 | 1 | 0.59 |
| Sema6d | NM_172537 | 1 | 1 | 0.58 |
| Tmem164 | NM_001199360 | 1 | 1 | 0.58 |
| Lpin1 | NM_001130412 | 1 | 1 | 0.58 |
| Synj2bp | NM_025292 | 1 | 0 | 0.58 |
| Ppm1b | NM_011151 | 1 | 0 | 0.58 |
| Hs3st1 | NM_010474 | 1 | 0 | 0.58 |
| Cdyl | NM_001123386 | 1 | 0 | 0.58 |
| Ptpn13 | NM_011204 | 1 | 1 | 0.58 |
| Ppp1r9b | NM_172261 | 1 | 0 | 0.58 |
| Phf21b | NM_001081166 | 1 | 0 | 0.58 |
| Surf4 | NM_011512 | 1 | 0 | 0.58 |
| Efna1 | NM_001162425 | 1 | 0 | 0.58 |
| Arhgap6 | NM_009707 | 1 | 0 | 0.58 |
| Klhl29 | NM_001164493 | 1 | 0 | 0.58 |
| Csnk1g1 | NM_173185 | 1 | 0 | 0.57 |
| Fam76b | NM_176836 | 1 | 0 | 0.57 |
| Gpr146 | NM_001038703 | 1 | 1 | 0.57 |
| Aplp2 | NM_001102455 | 1 | 0 | 0.57 |
| Bmi1 | NM_007552 | 1 | 0 | 0.57 |
| Sox2 | NM_011443 | 1 | 0 | 0.57 |
| Prkar2b | NM_011158 | 1 | 0 | 0.57 |
| Mkl2 | NM_001122667 | 1 | 1 | 0.57 |
| Cntfr | NM_001136056 | 1 | 0 | 0.57 |
| Gem | NM_010276 | 1 | 0 | 0.57 |
| Ddx3x | NM_010028 | 1 | 0 | 0.56 |
| D19Wsu162e | NM_001177812 | 1 | 0 | 0.56 |
| Pan3 | NM_028291 | 1 | 0 | 0.56 |
| Mpp5 | NM_019579 | 1 | 0 | 0.56 |
| Kcnb2 | NM_001098528 | 1 | 0 | 0.56 |
| Tbp | NM_013684 | 1 | 0 | 0.56 |
| Wapal | NM_001004436 | 1 | 0 | 0.55 |
| Cep350 | NM_001039184 | 1 | 0 | 0.55 |
| Cdh20 | NM_011800 | 1 | 1 | 0.55 |
| Mapk7 | NM_011841 | 1 | 0 | 0.55 |
| Trip4 | NM_001170907 | 1 | 0 | 0.55 |
| Bag5 | NM_027404 | 1 | 1 | 0.55 |
| Prdm1 | NM_007548 | 1 | 0 | 0.55 |
| Taf4a | NM_001081092 | 1 | 0 | 0.54 |
| Elmod1 | NM_177769 | 1 | 1 | 0.54 |
| Aebp2 | NM_001005605 | 1 | 0 | 0.54 |
| Cbx4 | NM_007625 | 2 | 0 | 0.54 |
| Ube2d1 | NM_145420 | 1 | 0 | 0.54 |
| Arid4b | NM_194262 | 1 | 0 | 0.54 |
| Hnf1b | NM_009330 | 1 | 0 | 0.54 |
| Rnf5 | NM_019403 | 1 | 0 | 0.54 |
| Asph | NM_001177849 | 1 | 0 | 0.54 |
| Ip6k1 | NM_013785 | 1 | 0 | 0.54 |
| Tardbp | NM_145556 | 1 | 0 | 0.53 |
| Skor1 | NM_001163755 | 1 | 0 | 0.53 |
| Oxr1 | NM_001130163 | 1 | 0 | 0.53 |
| Rlf | NM_001081013 | 1 | 0 | 0.53 |
| Tcf4 | NM_001083967 | 1 | 1 | 0.53 |
| Pigh | NM_029988 | 1 | 1 | 0.53 |
| Atp11c | NM_001001798 | 1 | 0 | 0.53 |
| Jakmip3 | NM_028708 | 1 | 2 | 0.53 |
| Nup107 | NM_134010 | 1 | 0 | 0.53 |
| Mtfr1 | NM_026182 | 1 | 1 | 0.53 |
| Ndn | NM_010882 | 1 | 0 | 0.53 |
| Sulf1 | NM_001198565 | 1 | 0 | 0.53 |
| Slco5a1 | NM_172841 | 1 | 2 | 0.52 |
| Rnf2 | NM_011277 | 1 | 0 | 0.52 |
| Naa50 | NM_028108 | 1 | 3 | 0.52 |
| Tmem188 | NM_029074 | 2 | 0 | 0.52 |
| Kctd15 | NM_146188 | 1 | 0 | 0.52 |
| Rfx7 | NM_001033536 | 1 | 1 | 0.52 |
| Lats2 | NM_015771 | 1 | 2 | 0.52 |
| Mblac2 | NM_028372 | 1 | 2 | 0.52 |
| Trak2 | NM_172406 | 1 | 0 | 0.51 |
| Cblb | NM_001033238 | 1 | 0 | 0.51 |
| Armc2 | NM_001034858 | 1 | 0 | 0.51 |
| Slc35e2 | NM_177186 | 1 | 0 | 0.51 |
| Trim44 | NM_020267 | 1 | 0 | 0.5 |
| Prkar1a | NM_021880 | 1 | 0 | 0.5 |
| Rhoa | NM_016802 | 1 | 0 | 0.5 |
| Col4a3bp | NM_001164222 | 1 | 2 | 0.5 |
| Kcna2 | NM_008417 | 1 | 1 | 0.5 |
| Btrc | NM_001037758 | 1 | 0 | 0.5 |
| Sbf1 | NM_001081030 | 1 | 0 | 0.5 |
| Tbl1xr1 | NM_030732 | 1 | 0 | 0.5 |
| Ocln | NM_008756 | 1 | 0 | 0.5 |
| Pum2 | NM_001160219 | 1 | 1 | 0.49 |
| A830080D01Rik | NM_001033472 | 1 | 0 | 0.49 |
| Trio | NM_001081302 | 1 | 1 | 0.49 |
| Ap4e1 | NM_175550 | 1 | 1 | 0.49 |
| Rusc2 | NM_001037709 | 2 | 0 | 0.49 |
| Fndc3b | NM_173182 | 1 | 1 | 0.49 |
| Hic2 | NM_178922 | 1 | 0 | 0.49 |
| Mdm4 | NM_008575 | 1 | 2 | 0.48 |
| Avl9 | NM_030235 | 1 | 0 | 0.48 |
| Cnn3 | NM_028044 | 1 | 0 | 0.48 |
| Nanos1 | NM_178421 | 1 | 2 | 0.48 |
| Csnk1g3 | NM_152809 | 1 | 0 | 0.47 |
| Schip1 | NM_001113420 | 1 | 0 | 0.47 |
| Iqcj-schip1 | NM_001113419 | 1 | 0 | 0.47 |
| Cish | NM_009895 | 1 | 0 | 0.47 |
| Rimklb | NM_027664 | 1 | 1 | 0.47 |
| Gosr2 | NM_019650 | 1 | 0 | 0.47 |
| Shroom1 | NM_027917 | 1 | 0 | 0.47 |
| Klf4 | NM_010637 | 1 | 0 | 0.47 |
| Ppara | NM_001113418 | 1 | 0 | 0.47 |
| Sp1 | NM_013672 | 1 | 0 | 0.46 |
| Rab21 | NM_024454 | 1 | 0 | 0.46 |
| Atp2a2 | NM_001110140 | 1 | 0 | 0.46 |
| Dgka | NM_016811 | 1 | 0 | 0.46 |
| Neo1 | NM_001042752 | 1 | 0 | 0.46 |
| Bnip3l | NM_009761 | 1 | 1 | 0.46 |
| Ank1 | NM_001110783 | 1 | 0 | 0.46 |
| Appl1 | NM_145221 | 1 | 0 | 0.46 |
| Basp1 | NM_027395 | 1 | 0 | 0.45 |
| Lrrtm3 | NM_178678 | 1 | 0 | 0.45 |
| Arid2 | NM_175251 | 1 | 1 | 0.45 |
| Zmym4 | NM_001114399 | 1 | 0 | 0.45 |
| Reep3 | NM_001204915 | 2 | 0 | 0.45 |
| Sec61a2 | NM_021305 | 1 | 0 | 0.45 |
| B3gnt2 | NM_001169114 | 1 | 0 | 0.45 |
| Aak1 | NM_001040106 | 1 | 3 | 0.45 |
| Cdk12 | NM_001109626 | 1 | 0 | 0.45 |
| Smarcd1 | NM_031842 | 1 | 0 | 0.44 |
| Lrrc8a | NM_177725 | 1 | 0 | 0.44 |
| G6pc | NM_008061 | 1 | 0 | 0.44 |
| Fam126b | NM_172513 | 1 | 3 | 0.44 |
| Kdm4a | NM_001161823 | 1 | 0 | 0.44 |
| Ddx26b | NM_172779 | 1 | 0 | 0.44 |
| Matr3 | NM_010771 | 1 | 0 | 0.44 |
| Ankrd52 | NM_172790 | 1 | 0 | 0.44 |
| Phf6 | NM_027642 | 1 | 2 | 0.44 |
| Pcdh19 | NM_001105245 | 2 | 0 | 0.44 |
| Txlng | NM_178935 | 1 | 0 | 0.43 |
| Chrdl1 | NM_001114385 | 1 | 0 | 0.43 |
| Cntn4 | NM_001109749 | 1 | 0 | 0.43 |
| Tln1 | NM_011602 | 2 | 0 | 0.43 |
| Fam3c | NM_138587 | 1 | 0 | 0.43 |
| Ptpn12 | NM_011203 | 2 | 0 | 0.43 |
| Ppp6r3 | NM_001164159 | 1 | 1 | 0.43 |
| 1700034H14Rik | NM_025969 | 1 | 0 | 0.43 |
| Scn5a | NM_021544 | 2 | 0 | 0.42 |
| Lin7b | NM_011698 | 1 | 0 | 0.42 |
| Cfl2 | NM_007688 | 1 | 1 | 0.42 |
| Slc6a17 | NM_172271 | 1 | 0 | 0.42 |
| Mtus1 | NM_001005863 | 1 | 0 | 0.42 |
| Actr1a | NM_016860 | 1 | 1 | 0.42 |
| Sdc2 | NM_008304 | 1 | 0 | 0.41 |
| Rac1 | NM_009007 | 1 | 0 | 0.41 |
| Foxp1 | NM_001197321 | 2 | 0 | 0.41 |
| Gjc1 | NM_001159382 | 1 | 0 | 0.41 |
| Frmd4b | NM_145148 | 1 | 0 | 0.41 |
| Kcnq4 | NM_001081142 | 1 | 0 | 0.41 |
| Trim62 | NM_178110 | 1 | 0 | 0.4 |
| Rps6ka2 | NM_011299 | 1 | 0 | 0.4 |
| Blcap | NM_016916 | 1 | 2 | 0.4 |
| Ccny | NM_026484 | 1 | 0 | 0.4 |
| Fam168b | NM_001160235 | 1 | 2 | 0.4 |
| Tril | NM_025817 | 1 | 0 | 0.4 |
| Rprd1a | NM_144861 | 1 | 0 | 0.39 |
| Rab8b | NM_173413 | 1 | 0 | 0.39 |
| Hccs | NM_008222 | 1 | 0 | 0.39 |
| Map4k3 | NM_001081357 | 1 | 1 | 0.39 |
| Cacna1c | NM_001159533 | 1 | 0 | 0.39 |
| Reck | NM_016678 | 1 | 0 | 0.39 |
| Nrip3 | NM_020610 | 1 | 1 | 0.39 |
| Hecw2 | NM_001001883 | 1 | 1 | 0.39 |
| Mxd3 | NM_016662 | 1 | 0 | 0.39 |
| Pls3 | NM_001166453 | 1 | 0 | 0.38 |
| Zbtb33 | NM_001079513 | 1 | 0 | 0.38 |
| Soat1 | NM_009230 | 1 | 1 | 0.38 |
| Styx | NM_019637 | 1 | 2 | 0.38 |
| Rdh10 | NM_133832 | 1 | 0 | 0.38 |
| Tmem170 | NM_025781 | 1 | 0 | 0.38 |
| Mmp16 | NM_019724 | 1 | 0 | 0.37 |
| Pcdh8 | NM_001042726 | 1 | 0 | 0.37 |
| Fyn | NM_001122892 | 1 | 1 | 0.36 |
| Dot1l | NM_199322 | 1 | 0 | 0.36 |
| Sash1 | NM_175155 | 1 | 0 | 0.36 |
| Rabif | NM_145510 | 1 | 0 | 0.36 |
| Hs3st3a1 | NM_178870 | 1 | 0 | 0.36 |
| Apoo | NM_001199337 | 1 | 1 | 0.36 |
| Cdon | NM_021339 | 1 | 0 | 0.36 |
| Ppp1r10 | NM_001163818 | 1 | 0 | 0.36 |
| Prkab1 | NM_031869 | 1 | 0 | 0.36 |
| Thoc2 | NM_001033422 | 1 | 0 | 0.35 |
| Tex2 | NM_198292 | 1 | 0 | 0.35 |
| Dcaf7 | NM_027946 | 1 | 0 | 0.35 |
| Scn3a | NM_018732 | 1 | 0 | 0.35 |
| Crtap | NM_019922 | 1 | 1 | 0.34 |
| Erlin1 | NM_001164359 | 1 | 0 | 0.34 |
| Xkr6 | NM_173393 | 1 | 0 | 0.34 |
| Nrbf2 | NM_001036293 | 1 | 0 | 0.34 |
| Dact1 | NM_001190466 | 1 | 0 | 0.34 |
| Lhx9 | NM_001025565 | 1 | 1 | 0.34 |
| Edc3 | NM_153799 | 1 | 0 | 0.34 |
| Akt3 | NM_011785 | 1 | 0 | 0.34 |
| Ap3s1 | NM_009681 | 1 | 0 | 0.34 |
| Ulk2 | NM_013881 | 1 | 0 | 0.33 |
| Heatr5a | NM_177171 | 1 | 0 | 0.33 |
| Crk | NM_133656 | 1 | 0 | 0.33 |
| Kdm3b | NM_001081256 | 1 | 0 | 0.33 |
| Nrp2 | NM_001077403 | 1 | 0 | 0.33 |
| Cyp1b1 | NM_009994 | 1 | 1 | 0.33 |
| Cab39 | NM_133781 | 1 | 0 | 0.33 |
| Cdkn2aip | NM_172407 | 1 | 0 | 0.32 |
| Tial1 | NM_009383 | 1 | 0 | 0.32 |
| Tjp1 | NM_001163574 | 1 | 1 | 0.32 |
| Gpr158 | NM_001004761 | 1 | 0 | 0.32 |
| Xpr1 | NM_011273 | 1 | 0 | 0.32 |
| Lca5 | NM_027448 | 1 | 1 | 0.31 |
| Rnf38 | NM_001038993 | 1 | 0 | 0.31 |
| Vegfa | NM_001025250 | 1 | 0 | 0.31 |
| Tmod3 | NM_016963 | 1 | 1 | 0.31 |
| 1200011M11Rik | NM_024262 | 1 | 0 | 0.31 |
| Dcp2 | NM_027490 | 1 | 2 | 0.31 |
| Fmnl2 | NM_172409 | 1 | 0 | 0.31 |
| Zfp704 | NM_133218 | 1 | 0 | 0.3 |
| Adcy2 | NM_153534 | 1 | 0 | 0.3 |
| 3110082D06Rik | NM_028474 | 1 | 1 | 0.3 |
| Adamts9 | NM_175314 | 1 | 0 | 0.3 |
| Wdfy3 | NM_172882 | 1 | 0 | 0.29 |
| Tceb1 | NM_026456 | 1 | 1 | 0.29 |
| Actr3 | NM_001205385 | 1 | 0 | 0.29 |
| Hipk1 | NM_010432 | 1 | 0 | 0.28 |
| Plk2 | NM_152804 | 1 | 0 | 0.28 |
| Col4a3 | NM_007734 | 1 | 1 | 0.27 |
| Msl2 | NM_001100451 | 1 | 0 | 0.27 |
| Slc5a3 | NM_017391 | 1 | 3 | 0.27 |
| Phtf2 | NM_172992 | 1 | 0 | 0.26 |
| Igsf10 | NM_001162884 | 1 | 0 | 0.26 |
| Nedd4l | NM_001114386 | 1 | 0 | 0.26 |
| Jmy | NM_021310 | 1 | 0 | 0.26 |
| Syne1 | NM_153399 | 1 | 1 | 0.26 |
| Foxn2 | NM_180974 | 1 | 1 | 0.26 |
| Prkca | NM_011101 | 1 | 2 | 0.26 |
| Serpinc1 | NM_080844 | 1 | 0 | 0.26 |
| Ptpn11 | NM_001109992 | 1 | 1 | 0.26 |
| Zfp395 | NM_199029 | 1 | 0 | 0.26 |
| Klf13 | NM_021366 | 1 | 0 | 0.26 |
| Cask | NM_009806 | 1 | 0 | 0.26 |
| Cggbp1 | NM_178647 | 1 | 0 | 0.26 |
| Prtg | NM_175485 | 1 | 0 | 0.26 |
| Snx30 | NM_172468 | 1 | 0 | 0.26 |
| Pppde2 | NM_134095 | 1 | 0 | 0.26 |
| Caskin1 | NM_027937 | 1 | 0 | 0.26 |
| Snx13 | NM_001014973 | 1 | 1 | 0.26 |
| Setd7 | NM_080793 | 1 | 0 | 0.26 |
| Arih2 | NM_011790 | 1 | 1 | 0.26 |
| Lrig1 | NM_008377 | 1 | 0 | 0.26 |
| Mrps25 | NM_025578 | 1 | 0 | 0.26 |
| Gad2 | NM_008078 | 1 | 0 | 0.26 |
| Fbxl16 | NM_001164225 | 1 | 0 | 0.26 |
| Aff4 | NM_033565 | 1 | 0 | 0.26 |
| Senp5 | NM_177103 | 1 | 0 | 0.26 |
| Sephs1 | NM_175400 | 1 | 1 | 0.26 |
| Atp11a | NM_015804 | 1 | 0 | 0.26 |
| Arcn1 | NM_145985 | 1 | 0 | 0.26 |
| Secisbp2l | NM_177608 | 1 | 0 | 0.26 |
| Irs1 | NM_010570 | 1 | 0 | 0.26 |
| Tpcn1 | NM_145853 | 1 | 0 | 0.26 |
| Creb1 | NM_001037726 | 1 | 1 | 0.25 |
| Pde7b | NM_013875 | 1 | 0 | 0.25 |
| Osr1 | NM_011859 | 1 | 0 | 0.25 |
| Mex3a | NM_001029890 | 1 | 0 | 0.25 |
| Grem2 | NM_011825 | 1 | 0 | 0.25 |
| Fam107b | NM_025626 | 1 | 1 | 0.25 |
| Ddit4 | NM_029083 | 1 | 0 | 0.25 |
| Dmd | NM_007868 | 1 | 0 | 0.25 |
| Dcaf5 | NM_177267 | 1 | 0 | 0.25 |
| Nfic | NM_008688 | 1 | 0 | 0.25 |
| Pak3 | NM_001195046 | 1 | 0 | 0.25 |
| Arl2bp | NM_024191 | 1 | 0 | 0.25 |
| B4galt6 | NM_019737 | 1 | 2 | 0.25 |
| Dnajb9 | NM_013760 | 1 | 0 | 0.25 |
| Lpin2 | NM_001164885 | 1 | 1 | 0.25 |
| Eif3j | NM_144545 | 1 | 0 | 0.25 |
| Serf2 | NM_011354 | 1 | 0 | 0.25 |
| Mxd4 | NM_010753 | 1 | 0 | 0.25 |
| Gtf2e1 | NM_028812 | 1 | 0 | 0.25 |
| Mgat3 | NM_010795 | 1 | 0 | 0.25 |
| Ppp2r5e | NM_012024 | 1 | 0 | 0.25 |
| Celf2 | NM_001110228 | 1 | 0 | 0.25 |
| Lpl | NM_008509 | 1 | 0 | 0.25 |
| Fam23a | NM_001081310 | 1 | 0 | 0.25 |
| Zfhx4 | NM_030708 | 1 | 0 | 0.24 |
| Nrbp1 | NM_147201 | 1 | 0 | 0.24 |
| Gcom1 | NM_001033208 | 1 | 1 | 0.24 |
| Frmd6 | NM_028127 | 1 | 0 | 0.24 |
| Lemd3 | NM_001081193 | 1 | 0 | 0.24 |
| Trps1 | NM_032000 | 1 | 2 | 0.24 |
| Atxn1l | NM_001080930 | 1 | 0 | 0.24 |
| Wasf1 | NM_031877 | 1 | 1 | 0.24 |
| Chst2 | NM_018763 | 1 | 0 | 0.24 |
| Ino80d | NM_001081436 | 1 | 1 | 0.24 |
| Tada2b | NM_001170454 | 1 | 0 | 0.24 |
| Elavl4 | NM_001038698 | 1 | 0 | 0.24 |
| Bicd2 | NM_001039179 | 1 | 1 | 0.24 |
| Cebpa | NM_007678 | 1 | 0 | 0.24 |
| Gab1 | NM_021356 | 1 | 0 | 0.24 |
| 41704 | NM_172606 | 1 | 0 | 0.24 |
| Atl2 | NM_019717 | 1 | 0 | 0.24 |
| Sgcb | NM_011890 | 1 | 1 | 0.23 |
| Nck2 | NM_010879 | 1 | 0 | 0.23 |
| Klhl24 | NM_029436 | 1 | 0 | 0.23 |
| Rab13 | NM_026677 | 1 | 0 | 0.23 |
| Mobkl3 | NM_025283 | 1 | 0 | 0.23 |
| Etf1 | NM_144866 | 1 | 0 | 0.23 |
| Maf | NM_001025577 | 1 | 0 | 0.23 |
| Pfn2 | NM_019410 | 1 | 0 | 0.23 |
| Shc1 | NM_001113331 | 1 | 0 | 0.23 |
| Sgms2 | NM_028943 | 1 | 0 | 0.23 |
| Ubxn7 | NM_177633 | 1 | 0 | 0.23 |
| Eps8l2 | NM_133191 | 1 | 0 | 0.23 |
| Pde5a | NM_153422 | 1 | 1 | 0.23 |
| Cadm1 | NM_001025600 | 1 | 0 | 0.23 |
| Adrb2 | NM_007420 | 1 | 0 | 0.23 |
| Pvrl1 | NM_021424 | 1 | 0 | 0.23 |
| Cgnl1 | NM_026599 | 1 | 0 | 0.23 |
| Cops2 | NM_009939 | 1 | 0 | 0.22 |
| Zfand6 | NM_022985 | 1 | 1 | 0.22 |
| Uba6 | NM_172712 | 2 | 0 | 0.17 |
| Wnk1 | NM_001185020 | 1 | 1 | < 0.1 |
| Mgat2 | NM_146035 | 1 | 1 | < 0.1 |
| Lrp1b | NM_053011 | 1 | 0 | < 0.1 |
| Acvr2a | NM_007396 | 1 | 0 | < 0.1 |
| Tsc22d1 | NM_001177751 | 1 | 0 | < 0.1 |
| Zc3h4 | NM_198631 | 1 | 0 | < 0.1 |
| Immp2l | NM_053122 | 1 | 1 | < 0.1 |
| Olfr558 | NM_147093 | 1 | 0 | < 0.1 |
| Fam48a | NM_019995 | 1 | 0 | < 0.1 |
| Adamts8 | NM_013906 | 1 | 0 | < 0.1 |
| Frem1 | NM_001198811 | 1 | 0 | < 0.1 |
| Pim2 | NM_138606 | 1 | 0 | < 0.1 |
| Fli1 | NM_008026 | 1 | 0 | < 0.1 |
| Tbx5 | NM_011537 | 1 | 0 | < 0.1 |
| Lhx5 | NM_008499 | 1 | 0 | < 0.1 |
| Syncrip | NM_019796 | 1 | 1 | < 0.1 |
| Ankrd40 | NM_027799 | 1 | 0 | < 0.1 |
| Polk | NM_012048 | 1 | 1 | < 0.1 |
| Fam179b | NM_177805 | 1 | 0 | < 0.1 |
| Kcnd2 | NM_019697 | 1 | 0 | < 0.1 |
| Ngef | NM_001111314 | 1 | 0 | < 0.1 |
| Slc6a15 | NM_175328 | 1 | 0 | < 0.1 |
| Dusp1 | NM_013642 | 1 | 0 | < 0.1 |
| Edem3 | NM_001039644 | 1 | 0 | < 0.1 |
| Ppp4r1 | NM_001114131 | 1 | 0 | < 0.1 |
| Gucy1a3 | NM_021896 | 1 | 0 | < 0.1 |
| Spesp1 | NM_025721 | 1 | 0 | < 0.1 |
| Aim1 | NM_172393 | 1 | 0 | < 0.1 |
| Dock4 | NM_172803 | 1 | 0 | < 0.1 |
| Arhgef7 | NM_001113517 | 1 | 1 | < 0.1 |
| Ccdc47 | NM_026009 | 1 | 0 | < 0.1 |
| Fosb | NM_008036 | 1 | 0 | < 0.1 |
| Cul5 | NM_001161618 | 1 | 0 | < 0.1 |
| Kif21b | NM_001039472 | 1 | 0 | < 0.1 |
| Mycn | NM_008709 | 1 | 0 | < 0.1 |
| Tbc1d2b | NM_194334 | 1 | 0 | < 0.1 |
| Eri3 | NM_080469 | 1 | 0 | < 0.1 |
|  |  |  |  |  |
|  |  |  |  |  |
|  |  |  |  |  |
| miR-1195 target gene | Representative transcript | Conserved sites | Poorly conserved sites |  |
|  |  | total | total |  |
| Lgi1 | NM_020278 | 1 | 1 | -0.43 |
| Pbx3 | NM_016768 | 1 | 0 | -0.37 |
| Il5ra | NM_008370 | 1 | 1 | -0.26 |
| Ptrh2 | NM_001098810 | 1 | 2 | -0.21 |
| Nr1d1 | NM_145434 | 1 | 0 | -0.2 |
| Zfp868 | NM_001045553 | 1 | 0 | -0.17 |
| Sh3d19 | NM_001082414 | 1 | 2 | -0.17 |
| Hapln1 | NM_013500 | 1 | 0 | -0.16 |
| Ssbp2 | NM_024186 | 1 | 1 | -0.16 |
| Hoxa5 | NM_010453 | 1 | 0 | -0.16 |
| Fam168a | NM_178764 | 1 | 0 | -0.16 |
| Serp1 | NM_030685 | 1 | 0 | -0.15 |
| D030016E14Rik | NM_177240 | 1 | 0 | -0.15 |
| Meis2 | NM_001136072 | 1 | 1 | -0.14 |
| Tnks2 | NM_001163635 | 1 | 0 | -0.13 |
| Rfx3 | NM_001166414 | 1 | 0 | -0.12 |
| Tpm2 | NM_009416 | 1 | 0 | -0.12 |
| Esco1 | NM_001081222 | 1 | 0 | -0.12 |
| Kcne3 | NM_001190869 | 1 | 0 | -0.12 |
| Fam46c | NM_001142952 | 1 | 0 | -0.1 |
| Pcdh17 | NM_001013753 | 1 | 0 | -0.1 |
| Stk4 | NM_021420 | 1 | 0 | -0.1 |
| Stk38l | NM_172734 | 1 | 0 | -0.1 |
| Hnrnpa1 | NM_001039129 | 1 | 0 | -0.1 |
| Zzz3 | NM_001080755 | 1 | 0 | -0.09 |
| 8430427H17Rik | NM_001001986 | 1 | 1 | -0.07 |
| Spire1 | NM_176832 | 1 | 0 | -0.07 |
| Arid2 | NM_175251 | 1 | 0 | -0.07 |
| Nova2 | NM_001029877 | 1 | 0 | -0.06 |
| Hunk | NM_015755 | 1 | 0 | -0.06 |
| Phlpp2 | NM_001122594 | 1 | 1 | -0.05 |
| Pclo | NM_001110796 | 1 | 0 | -0.04 |
| Reps2 | NM_178256 | 1 | 0 | -0.02 |
| Pgm3 | NM_001163746 | 1 | 0 | -0.02 |
| Elovl6 | NM_130450 | 1 | 0 | > -0.01 |
| Etv3 | NM_001083318 | 1 | 0 | > -0.01 |
| Map3k2 | NM_011946 | 1 | 0 | > -0.01 |
| Sema5a | NM_009154 | 1 | 0 | > -0.01 |
| Nsd1 | NM_008739 | 1 | 0 | > -0.01 |
| Bmp3 | NM_173404 | 1 | 0 | N/A |
