# Supplementary material for: Transcription factor and microRNA interactions in lung cells: an inhibitory link between NK2 homeobox 1, miR-200c and the developmental and oncogenic factors Nfib and Myb
Source: Respir Res. 2015 Feb 13;16(1):22. doi: 10.1186/s12931-015-0186-6 (PMC4335692; doi:10.1186/s12931-015-0186-6)
Supplement: Additional file 3: Table S2. — Gene expression changes induced by down-regulation of Nkx2-1 in MLE15 cells. [file 12931_2015_186_MOESM3_ESM.doc]

| *Table S2. Gene expression changes induced by down-regulation of Nkx2-1 in MLE15 cells. Adjusted p value < 0.0005* | | | | |
| --- | --- | --- | --- | --- |
| p ≤ 0.0005 | | | | |
| **Gene.Symbol** | **logFC** | **adj.P.Val** | **mRNA.Accession** | **Chromosome** |
| Abcg2 | -5.52 | 1.1E-07 | NM_011920 | chr6 |
| Plunc | -5.23 | 1.1E-07 | NM_011126 | chr2 |
| Rorb | -5.17 | 1.1E-07 | NM_001043354 | chr19 |
| Ifitm3 | -3.96 | 1.4E-07 | NM_025378 | chr7 |
| Eid1 | -3.79 | 4.2E-07 | NM_025613 | chr2 |
| Sftpc | -3.65 | 3.8E-06 | NM_011359 | chr14 |
| Aldh1a7 | -3.44 | 7.4E-07 | NM_011921 | chr19 |
| Slc43a3 | -3.11 | 7.2E-07 | NM_021398 | chr2 |
| Osbpl6 | -3.04 | 9.2E-07 | NM_145525 | chr2 |
| 0610031J06Rik | -3.02 | 2.1E-06 | NM_020003 | chr3 |
| Sox2 | -2.94 | 2.1E-07 | NM_011443 | chr3 |
| Ranbp17 | -2.89 | 4.5E-07 | NM_023146 | chr11 |
| Lclat1 | -2.88 | 2.7E-06 | NM_001081071 | chr17 |
| Fras1 | -2.82 | 7.0E-07 | NM_175473 | chr5 |
| Gcg | -2.81 | 1.7E-06 | NM_008100 | chr2 |
| Arg2 | -2.79 | 1.3E-06 | NM_009705 | chr12 |
| --- | -2.76 | 1.1E-05 | NC_005089 | chrM |
| Ano6 | -2.72 | 1.4E-06 | NM_175344 | chr15 |
| Fam171b | -2.67 | 1.5E-06 | NM_175514 | chr2 |
| Gatm | -2.65 | 3.9E-06 | NM_025961 | chr2 |
| Cd36 | -2.58 | 4.7E-06 | NM_007643 | chr5 |
| Pard3 | -2.55 | 3.0E-07 | NM_033620 | chr8 |
| Rragb | -2.55 | 2.6E-05 | NM_001004154 | chrX |
| Casc1 | -2.51 | 1.3E-06 | NM_177222 | chr6 |
| Pros1 | -2.51 | 1.6E-06 | NM_011173 | chr16 |
| Ros1 | -2.45 | 1.1E-06 | NM_011282 | chr10 |
| Gkn2 | -2.44 | 7.5E-06 | NM_025467 | chr6 |
| Tmem176b | -2.41 | 8.9E-07 | NM_023056 | chr6 |
| Gbp6 | -2.39 | 9.4E-06 | NM_001083312 | chr3 |
| Myct1 | -2.35 | 7.1E-05 | NM_026793 | chr10 |
| Aldh1a1 | -2.32 | 9.3E-07 | NM_013467 | chr19 |
| Mctp2 | -2.31 | 4.1E-06 | NM_001024703 | chr7 |
| Akr1d1 | -2.24 | 7.7E-06 | NM_145364 | chr6 |
| Pde7b | -2.20 | 9.2E-07 | NM_013875 | chr10 |
| Rapgef5 | -2.17 | 3.3E-06 | NM_175930 | chr12 |
| Sntb1 | -2.10 | 1.4E-06 | NM_016667 | chr15 |
| Arhgap29 | -2.03 | 4.1E-05 | NM_172525 | chr3 |
| Fabp4 | -2.02 | 1.2E-06 | NM_024406 | chr3 |
| Fmnl2 | -2.02 | 2.3E-05 | NM_172409 | chr2 |
| Plxna4 | -2.02 | 6.5E-07 | NM_175750 | chr6 |
| 4933404M02Rik | -2.02 | 2.2E-06 | NM_025744 | chr2 |
| Tek | -2.00 | 1.3E-05 | NM_013690 | chr4 |
| Has2 | -2.00 | 6.7E-05 | NM_008216 | chr15 |
| Smarca2 | -1.99 | 9.3E-07 | NM_011416 | chr19 |
| Nrp1 | -1.99 | 2.3E-06 | NM_008737 | chr8 |
| --- | -1.94 | 3.9E-06 | NC_005089 | chrM |
| Bace2 | -1.93 | 1.4E-05 | NM_019517 | chr16 |
| Gpx8 | -1.93 | 1.6E-05 | NM_027127 | chr13 |
| Fyn | -1.92 | 6.6E-06 | NM_001122893 | chr10 |
| Lrp2 | -1.91 | 5.3E-06 | NM_001081088 | chr2 |
| 9230110C19Rik | -1.91 | 4.2E-06 | BC115525 | chr9 |
| Fam115a | -1.91 | 2.4E-05 | NM_029930 | chr6 |
| --- | -1.90 | 1.3E-05 | BC006770 | chr13 |
| Kng1 | -1.89 | 1.7E-05 | NM_001102411 | chr16 |
| --- | -1.88 | 2.0E-04 | ENSMUST00000082582 | chr10 |
| --- | -1.88 | 9.3E-06 | mmu-mir-344-1 | chr7 |
| Rgl1 | -1.84 | 7.0E-06 | NM_016846 | chr1 |
| Amigo2 | -1.83 | 5.2E-06 | NM_178114 | chr15 |
| --- | -1.83 | 7.0E-06 | mmu-mir-344-1 | chr7 |
| --- | -1.81 | 1.8E-06 | ENSMUST00000097478 | chr5 |
| --- | -1.81 | 1.8E-06 | ENSMUST00000097478 | chr5 |
| Wnk3 | -1.81 | 3.8E-05 | BC043119 | chrX |
| --- | -1.80 | 1.7E-06 | ENSMUST00000098286 | chr5 |
| --- | -1.80 | 1.7E-06 | ENSMUST00000097478 | chr5 |
| Vim | -1.80 | 3.8E-06 | NM_011701 | chr2 |
| EG620639 // 624421 // EG665031 | -1.79 | 1.7E-06 | ENSMUST00000115900 | chr5 |
| Thbs2 | -1.79 | 3.4E-05 | NM_011581 | chr17 |
| Aldh1l1 | -1.77 | 1.1E-06 | NM_027406 | chr6 |
| Kcnh1 | -1.76 | 1.4E-05 | NM_010600 | chr1 |
| Mtmr9 | -1.76 | 1.4E-05 | NM_177594 | chr14 |
| Aebp1 | -1.75 | 2.1E-05 | NM_009636 | chr11 |
| --- | -1.75 | 1.9E-06 | ENSMUST00000097478 | chr5 |
| Cubn | -1.75 | 4.2E-05 | NM_001081084 | chr2 |
| Trps1 | -1.75 | 6.9E-05 | NM_032000 | chr15 |
| --- | -1.75 | 1.8E-06 | ENSMUST00000098286 | chr5 |
| Wdr78 | -1.75 | 1.1E-05 | NM_146254 | chr4 |
| Kcnh5 | -1.74 | 1.7E-06 | NM_172805 | chr12 |
| --- | -1.74 | 1.8E-06 | ENSMUST00000098286 | chr5 |
| Galnt7 | -1.74 | 7.0E-06 | NM_144731 | chr8 |
| Kng2 | -1.73 | 9.3E-06 | NM_201375 | chr16 |
| 2900055J20Rik | -1.73 | 4.2E-05 | ENSMUST00000096572 | chr18 |
| Pltp | -1.73 | 3.8E-06 | NM_011125 | chr2 |
| Kctd16 | -1.73 | 9.4E-06 | AK220381 | chr18 |
| Vcan | -1.72 | 6.9E-06 | NM_001081249 | chr13 |
| Gbp3 | -1.70 | 1.3E-04 | NM_018734 | chr3 |
| Tmem100 | -1.69 | 6.2E-06 | NM_026433 | chr11 |
| Acat2 | -1.68 | 3.0E-06 | NM_009338 | chr17 |
| Sesn1 | -1.68 | 5.0E-06 | NM_001013370 | chr10 |
| Aox1 | -1.67 | 1.6E-05 | NM_009676 | chr1 |
| Il13ra1 | -1.66 | 6.8E-06 | NM_133990 | chrX |
| Sdpr | -1.65 | 1.7E-05 | NM_138741 | chr1 |
| Rdh11 | -1.64 | 4.7E-05 | NM_021557 | chr12 |
| Mx1 | -1.64 | 3.1E-05 | NM_010846 | chr16 |
| Dub1a | -1.64 | 4.5E-06 | NM_201409 | chr7 |
| Hhex | -1.63 | 1.9E-06 | NM_008245 | chr19 |
| --- | -1.62 | 2.7E-06 | ENSMUST00000097478 | chr5 |
| Tmem176a | -1.62 | 7.5E-06 | NM_025326 | chr6 |
| Hecw1 | -1.61 | 7.1E-05 | NM_001081348 | chr13 |
| Cacna1c | -1.61 | 3.7E-05 | NM_009781 | chr6 |
| Dpp4 | -1.61 | 1.9E-04 | NM_010074 | chr2 |
| 2310067E19Rik | -1.60 | 3.1E-05 | BC066147 | chr4 |
| Cxcr4 | -1.59 | 6.1E-06 | NM_009911 | chr1 |
| Itga1 | -1.59 | 1.5E-04 | NM_001033228 | chr13 |
| Galnt5 | -1.58 | 3.9E-05 | NM_172855 | chr2 |
| Lss | -1.56 | 2.1E-05 | NM_146006 | chr10 |
| Rcn1 | -1.56 | 4.1E-06 | NM_009037 | chr2 |
| A2bp1 | -1.55 | 9.5E-05 | NM_021477 | chr16 |
|  | -1.54 | 3.2E-06 |  |  |
| --- | -1.54 | 3.2E-06 | ENSMUST00000098286 | chr5 |
| --- | -1.54 | 3.2E-06 | ENSMUST00000098286 | chr5 |
| --- | -1.54 | 3.2E-06 | ENSMUST00000098286 | chr5 |
| Armcx1 | -1.53 | 1.7E-04 | NM_030066 | chrX |
| Mns1 | -1.53 | 8.7E-06 | NM_008613 | chr9 |
| Trps1 | -1.53 | 6.5E-06 | NM_032000 | chr15 |
|  | -1.52 | 7.2E-06 |  |  |
| EG622744 // EG666209 | -1.51 | 3.8E-06 | ENSMUST00000101011 | chr5 |
| Atp13a5 | -1.51 | 1.3E-05 | NM_175650 | chr16 |
| Mertk | -1.51 | 3.7E-05 | NM_008587 | chr2 |
| Igfbp5 | -1.51 | 1.9E-05 | NM_010518 | chr1 |
| --- | -1.50 | 1.4E-05 | ENSMUST00000098286 | chr5 |
| Mylip | -1.49 | 1.4E-05 | NM_153789 | chr13 |
| Arg1 | -1.49 | 9.1E-06 | NM_007482 | chr10 |
| Creg1 | -1.48 | 1.7E-05 | NM_011804 | chr1 |
| Idi1 | -1.48 | 1.7E-05 | NM_145360 | chr2 |
| Tle3 | -1.45 | 4.4E-06 | NM_001083927 | chr9 |
| Atp13a4 | -1.45 | 5.2E-05 | NM_172613 | chr16 |
| Idi1 | -1.45 | 3.5E-05 | NM_177960 | chr13 |
| Scn2a1 | -1.45 | 9.3E-05 | NM_001099298 | chr2 |
|  | -1.44 | 5.3E-06 |  |  |
| Cap2 | -1.43 | 8.4E-05 | NM_026056 | chr13 |
| Dtna | -1.43 | 2.0E-05 | NM_207650 | chr18 |
| Pik3r3 | -1.43 | 1.2E-05 | NM_181585 | chr4 |
| Rnf130 | -1.43 | 1.6E-04 | NM_021540 | chr11 |
| Hist1h2bc | -1.42 | 5.4E-05 | NM_023422 | chr13 |
| Fam122b | -1.42 | 4.2E-05 | NM_030167 | chrX |
| Hectd2 | -1.42 | 1.6E-05 | NM_172637 | chr19 |
| Fzd6 | -1.42 | 3.6E-04 | NM_008056 | chr15 |
| Rragd | -1.41 | 9.4E-06 | NM_027491 | chr4 |
| Sh3rf2 | -1.41 | 2.1E-05 | NM_172966 | chr18 |
| 1110032A04Rik | -1.40 | 1.4E-04 | NM_133675 | chr3 |
| Cul7 | -1.40 | 5.9E-06 | NM_025611 | chr17 |
| Chst4 | -1.40 | 3.9E-06 | NM_011998 | chr8 |
| Arl5a | -1.39 | 3.9E-06 | NM_182994 | chr2 |
| Mfsd7c | -1.38 | 1.4E-05 | NM_145447 | chr12 |
| Fancm | -1.37 | 2.8E-05 | NM_178912 | chr12 |
| Cd109 | -1.36 | 7.1E-05 | NM_153098 | chr9 |
| Pyy | -1.36 | 1.5E-04 | NM_145435 | chr11 |
| Gba | -1.35 | 3.7E-05 | NM_008094 | chr3 |
| Zfp334 | -1.34 | 1.3E-05 | NM_178411 | chr2 |
| --- | -1.34 | 3.2E-04 | ENSMUST00000082857 | chr9 |
| Kcnj15 | -1.34 | 1.5E-04 | NM_019664 | chr16 |
| Tspan13 | -1.34 | 6.2E-06 | NM_025359 | chr12 |
| Gpx2 | -1.34 | 1.9E-05 | NM_030677 | chr12 |
| Rbmx | -1.34 | 5.6E-05 | NM_011252 | chrX |
| Dclk2 | -1.33 | 7.0E-05 | NM_027539 | chr3 |
| Ifitm1 | -1.33 | 8.2E-06 | NM_026820 | chr8 |
| Ret | -1.32 | 9.5E-05 | NM_001080780 | chr6 |
| Mthfd1 | -1.32 | 4.1E-06 | NM_138745 | chr12 |
| Csf1 | -1.32 | 7.5E-06 | NM_007778 | chr3 |
| Echdc1 | -1.32 | 8.6E-05 | NM_025855 | chr10 |
| Zhx3 | -1.31 | 1.7E-05 | NM_177263 | chr2 |
| 1700001P01Rik | -1.31 | 3.0E-05 | NM_028156 | chr11 |
| --- | -1.31 | 7.6E-06 | --- | chr7 |
| Pvrl3 | -1.30 | 3.1E-05 | NM_021496 | chr16 |
| Tspan1 | -1.30 | 5.4E-05 | NM_133681 | chr4 |
| Aloxe3 | -1.30 | 6.0E-05 | NM_011786 | chr11 |
| Hsph1 | -1.29 | 6.1E-06 | NM_013559 | chr5 |
| Prelp | -1.29 | 1.0E-05 | NM_054077 | chr1 |
| Man2b1 | -1.29 | 4.5E-05 | NM_010764 | chr8 |
| Ank3 | -1.29 | 7.3E-05 | NM_146005 | chr10 |
| 2310007H09Rik | -1.28 | 3.6E-04 | NM_029609 | chr7 |
| Nsdhl | -1.28 | 3.0E-05 | NM_010941 | chrX |
| Dub2a | -1.28 | 3.1E-05 | NM_001001559 | chr7 |
| Cstf2t | -1.28 | 8.0E-05 | NM_031249 | chr19 |
| Fam102b | -1.28 | 7.4E-06 | ENSMUST00000046924 | chr3 |
| 1200002N14Rik | -1.28 | 9.3E-06 | NM_027878 | chr10 |
| --- | -1.28 | 2.2E-05 | ENSMUST00000111396 | chr7 |
| Six1 | -1.27 | 2.2E-04 | NM_009189 | chr12 |
| 2010107G12Rik | -1.27 | 9.8E-05 | BC075651 | chr6 |
| Gpr126 | -1.26 | 2.2E-05 | NM_001002268 | chr10 |
| Trrap | -1.26 | 3.0E-05 | NM_001081362 | chr5 |
| --- | -1.25 | 2.0E-05 | ENSMUST00000111396 | chr7_random |
| Thsd1 | -1.25 | 6.5E-05 | NM_019576 | chr8 |
| Bend6 | -1.25 | 4.4E-05 | BC057378 | chr1 |
| 5730471H19Rik | -1.24 | 1.7E-05 | AK133873 | chr2 |
| Slain1 | -1.24 | 1.6E-05 | NM_198014 | chr14 |
| Tcfcp2l1 | -1.24 | 7.4E-06 | NM_023755 | chr1 |
| 2810030E01Rik | -1.24 | 5.2E-05 | NM_028317 | chr2 |
| Mlh3 | -1.23 | 3.1E-05 | NM_175337 | chr12 |
| Rnf144b | -1.23 | 1.8E-05 | NM_146042 | chr13 |
| Ogdhl | -1.23 | 1.3E-04 | NM_001081130 | chr14 |
| Dhcr7 | -1.23 | 4.6E-05 | NM_007856 | chr7 |
| Nptx1 | -1.23 | 2.9E-05 | NM_008730 | chr11 |
| Trrap | -1.23 | 1.5E-05 | NM_001081362 | chr5 |
| Enpp3 | -1.23 | 1.4E-04 | NM_134005 | chr10 |
| Ifitm1 | -1.23 | 1.2E-04 | NM_026820 | chr7 |
| --- | -1.22 | 4.6E-05 | ENSMUST00000111396 | chr7 |
| Bpgm | -1.22 | 2.8E-05 | NM_007563 | chr6 |
| Ces5 | -1.22 | 1.7E-04 | NM_172759 | chr8 |
| Idi2 | -1.22 | 4.2E-05 | NM_177197 | chr13 |
| Hmgcs1 | -1.22 | 9.5E-05 | NM_145942 | chr13_random |
| --- | -1.21 | 3.7E-04 | ENSMUST00000099180 | chr13 |
| Eya4 | -1.21 | 3.4E-05 | NM_010167 | chr10 |
| Ddt | -1.20 | 5.5E-05 | NM_010027 | chr10 |
| Nfib | -1.19 | 3.0E-05 | NM_001113209 | chr4 |
| Nexn | -1.19 | 3.2E-04 | NM_199465 | chr3 |
| Eml5 | -1.19 | 2.8E-04 | NM_001081191 | chr12 |
| Hdc | -1.18 | 4.3E-05 | NM_008230 | chr2 |
| Fuca2 | -1.18 | 5.5E-05 | NM_025799 | chr10 |
| Slc25a38 | -1.18 | 1.4E-04 | NM_144793 | chr9 |
| Slc7a7 | -1.18 | 1.8E-05 | NM_011405 | chr14 |
| Clp1 | -1.18 | 5.4E-05 | NM_133840 | chr2 |
| Sprr2b | -1.16 | 1.3E-04 | NM_011469 | chr3 |
| Lipg | -1.16 | 3.5E-04 | NM_010720 | chr18 |
| Foxred2 | -1.15 | 2.3E-05 | NM_001017983 | chr15 |
| Slc7a4 | -1.15 | 3.3E-04 | NM_144852 | chr16 |
| Dna2 | -1.15 | 2.1E-05 | NM_177372 | chr10 |
| Gkap1 | -1.15 | 4.3E-05 | NM_019832 | chr13 |
| Galk2 | -1.14 | 1.6E-05 | NM_175154 | chr2 |
| Hist1h2ac | -1.14 | 3.8E-04 | NM_178189 | chr13 |
| Cyp51 | -1.14 | 2.2E-04 | NM_020010 | chr5 |
| Zfp503 | -1.14 | 9.4E-06 | NM_145459 | chr14 |
| Rab27a | -1.14 | 1.3E-05 | NM_023635 | chr9 |
| Gstp1 | -1.13 | 2.8E-04 | NM_013541 | chr19 |
| Dzip1 | -1.13 | 4.2E-05 | NM_025943 | chr14 |
| Cpne2 | -1.13 | 2.8E-05 | NM_153507 | chr8 |
| Ivl | -1.13 | 1.2E-04 | NM_008412 | chr3 |
| Rasd2 | -1.13 | 5.6E-05 | NM_029182 | chr8 |
| Tspan12 | -1.13 | 8.9E-05 | NM_173007 | chr6 |
| 2210023G05Rik | -1.13 | 5.2E-05 | BC027185 | chr8 |
| Gnai1 | -1.12 | 8.0E-05 | NM_010305 | chr5 |
| Ddx26b | -1.12 | 6.2E-05 | NM_172779 | chrX |
| P2ry5 | -1.12 | 6.0E-05 | NM_175116 | chr14 |
| Myb | -1.12 | 4.6E-05 | NM_010848 | chr10 |
| Tlcd1 | -1.11 | 2.5E-05 | NM_026708 | chr11 |
| Mcam | -1.11 | 2.6E-05 | NM_023061 | chr9 |
| Atp5g3 | -1.11 | 1.4E-04 | NM_175015 | chr2 |
| Kalrn | -1.11 | 7.0E-05 | BC157950 | chr16 |
| Adcy2 | -1.11 | 2.6E-05 | NM_153534 | chr13 |
| Idi2 | -1.11 | 1.5E-04 | AY263178 | chr13 |
| Ankrd6 | -1.10 | 1.7E-05 | NM_001012450 | chr4 |
| Ddi2 | -1.10 | 1.3E-05 | NM_001017966 | chr4 |
| Mvd | -1.10 | 4.0E-04 | NM_138656 | chr8 |
| Fbln5 | -1.10 | 7.8E-05 | NM_011812 | chr12 |
| Snord16a | -1.10 | 9.5E-05 | AF357363 | chr9 |
| Pop1 | -1.10 | 2.3E-05 | NM_152894 | chr15 |
| Mycn | -1.09 | 3.5E-04 | NM_008709 | chr12 |
| Sgk1 | -1.09 | 1.5E-04 | NM_011361 | chr10 |
| Ttc21b | -1.09 | 8.0E-05 | NM_001047604 | chr2 |
| C230052I12Rik | -1.09 | 2.2E-05 | NM_178643 | chr7 |
| Wdr6 | -1.09 | 6.3E-05 | NM_031392 | chr9 |
| Lpcat1 | -1.09 | 4.3E-05 | NM_145376 | chr13 |
| Trpv2 | -1.09 | 4.6E-05 | NM_011706 | chr11 |
| Dusp19 | -1.09 | 1.9E-04 | NM_024438 | chr2 |
| Abcb6 | -1.08 | 4.1E-05 | NM_023732 | chr1 |
| Piwil4 | -1.08 | 4.4E-05 | NM_177905 | chr9 |
| Mylk | -1.08 | 3.3E-05 | NM_139300 | chr16 |
| Sqle | -1.08 | 5.7E-05 | NM_009270 | chr15 |
| Mfsd2 | -1.08 | 1.1E-04 | NM_029662 | chr4 |
| Mapre2 | -1.08 | 4.9E-05 | NM_153058 | chr18 |
| Znf512b | -1.08 | 2.9E-05 | BC056460 | chr2 |
| ENSMUSG00000066331 | -1.07 | 4.4E-05 | ENSMUST00000084967 | chr12 |
| Calr | -1.07 | 4.6E-05 | NM_007591 | chr8 |
| Nudt22 | -1.07 | 9.5E-05 | NM_026675 | chr19 |
| Acss2 | -1.07 | 1.6E-04 | NM_019811 | chr2 |
| Slc41a2 | -1.07 | 2.8E-04 | NM_177388 | chr10 |
| Ntf3 | -1.06 | 2.8E-05 | NM_008742 | chr6 |
| Ivd | -1.06 | 3.7E-05 | NM_019826 | chr2 |
| Dub1 | -1.06 | 1.6E-04 | NM_007887 | chr7 |
| Wif1 | -1.06 | 4.5E-05 | NM_011915 | chr10 |
| Mccc2 | -1.06 | 8.9E-05 | NM_030026 | chr13 |
| Nfix | -1.06 | 3.1E-05 | NM_001081981 | chr8 |
| Dpp7 | -1.06 | 4.0E-04 | NM_031843 | chr2 |
| 9230020A06Rik | -1.05 | 1.1E-04 | ENSMUST00000100923 | chr11 |
| Arfgap2 | -1.05 | 3.0E-05 | NM_023854 | chr2 |
| Zfp462 | -1.05 | 5.3E-05 | NM_172867 | chr4 |
| --- | -1.05 | 7.4E-05 | ENSMUST00000121585 | chr7 |
| Gtf3c6 | -1.05 | 1.4E-04 | NM_026113 | chr10 |
| Ifi30 | -1.04 | 1.7E-04 | NM_023065 | chr8 |
| Kbtbd4 | -1.04 | 2.3E-04 | NM_025991 | chr2 |
| Hexim1 | -1.04 | 4.7E-05 | NM_138753 | chr11 |
| Trf | -1.04 | 9.5E-05 | NM_133977 | chr9 |
| Pfn2 | -1.04 | 1.4E-04 | NM_019410 | chr3 |
| Gnptab | -1.04 | 2.5E-05 | NM_001004164 | chr10 |
| Tmem56 | -1.03 | 2.5E-04 | NM_178936 | chr3 |
| Cox7a1 | -1.03 | 2.2E-04 | NM_009944 | chr7 |
| BC061212 | -1.03 | 3.1E-05 | BC061212 | chr5 |
| BC061212 | -1.03 | 3.1E-05 | BC061212 | chr5 |
| Copz2 | -1.03 | 4.3E-05 | NM_019877 | chr11 |
| --- | -1.03 | 1.8E-04 | --- | chr2 |
| Etv1 | -1.03 | 2.4E-04 | BC005645 | chr13 |
| BC061212 | -1.02 | 1.2E-04 | BC061212 | chr5 |
| BC061212 | -1.02 | 1.2E-04 | BC061212 | chr5 |
| Pde5a | -1.02 | 9.9E-05 | NM_153422 | chr3 |
| --- | -1.02 | 4.5E-05 | NM_025998.1 | --- |
| D2Wsu81e | -1.02 | 5.7E-05 | BC078441 | chr2 |
| BC061212 | -1.01 | 2.5E-05 | BC061212 | chr5 |
| Nfib | -1.01 | 6.3E-05 | NM_001113209 | chr4 |
| 4930534B04Rik | -1.01 | 4.3E-05 | NM_181815 | chr12 |
| Atp6v0a4 | -1.01 | 1.1E-04 | NM_080467 | chr6 |
| Ylpm1 | -1.01 | 1.9E-04 | NM_178363 | chr12 |
| Atox1 | -1.00 | 1.1E-04 | NM_009720 | chr11 |
| BC061212 | -1.00 | 4.3E-05 | BC061212 | chr5 |
| A430089I19Rik | -1.00 | 4.3E-05 | NM_177913 | chr5 |
| A430089I19Rik | -1.00 | 4.3E-05 | NM_177913 | chr5 |
| 3110003A17Rik | -1.00 | 1.0E-04 | NM_028440 | chr10 |
| Mcts2 | -0.99 | 9.0E-05 | NM_025543 | chr2 |
| Cdca7 | -0.99 | 9.0E-05 | NM_025866 | chr2 |
| 0610007P14Rik | -0.99 | 1.4E-04 | BC004591 | chr12 |
| Myo5c | -0.99 | 2.3E-04 | NM_001081322 | chr9 |
| Ccbl2 | -0.98 | 8.4E-05 | NM_173763 | chr3 |
| Fig4 | -0.98 | 2.0E-04 | NM_133999 | chr10 |
| ENSMUSG00000074057 | -0.98 | 6.5E-05 | ENSMUST00000098356 | chr7 |
| Erh | -0.98 | 2.5E-04 | NM_007951 | chr12 |
| Rnf24 | -0.98 | 1.2E-04 | NM_178607 | chr2 |
| Dlx4 | -0.98 | 1.9E-04 | NM_007867 | chr11 |
| Asl | -0.97 | 2.2E-04 | NM_133768 | chr5 |
| Egln3 | -0.97 | 6.4E-05 | NM_028133 | chr12 |
| Ahsa1 | -0.97 | 4.4E-05 | NM_146036 | chr12 |
| Bcl2a1a | -0.97 | 9.0E-05 | NM_009742 | chr9 |
| BC061212 | -0.97 | 3.7E-05 | BC061212 | chr5 |
| BC061212 | -0.97 | 7.9E-05 | BC061212 | chr5 |
| Dhcr24 | -0.96 | 1.3E-04 | NM_053272 | chr4 |
| Mast1 | -0.96 | 9.1E-05 | NM_019945 | chr8 |
| Fzd4 | -0.96 | 5.4E-05 | NM_008055 | chr7 |
| Rcbtb2 | -0.96 | 1.0E-04 | NM_134083 | chr14 |
| Spnb1 | -0.96 | 3.4E-05 | NM_013675 | chr12 |
| Bcl2a1b | -0.95 | 3.4E-04 | NM_007534 | chr9 |
| Obfc2b | -0.95 | 5.1E-05 | NM_027257 | chr10 |
| Tnfrsf18 | -0.95 | 2.9E-04 | NM_009400 | chr4 |
| Pex3 | -0.95 | 4.6E-05 | NM_019961 | chr10 |
| Katnal2 | -0.95 | 1.6E-04 | NM_027721 | chr18 |
| Map3k12 | -0.95 | 8.1E-05 | NM_009582 | chr15 |
| Hspb1 | -0.95 | 1.9E-04 | NM_013560 | chr13 |
| Smpdl3b | -0.95 | 3.2E-04 | NM_133888 | chr4 |
| Rbm38 | -0.95 | 3.2E-04 | NM_019547 | chr2 |
| Six4 | -0.95 | 1.5E-04 | NM_011382 | chr12 |
| Tbc1d8 | -0.94 | 1.9E-04 | NM_018775 | chr1 |
| Chsy3 | -0.94 | 1.1E-04 | NM_001081328 | chr18 |
| Tspan33 | -0.94 | 1.8E-04 | NM_146173 | chr6 |
| Fbn2 | -0.94 | 1.0E-04 | NM_010181 | chr18 |
| Brsk1 | -0.94 | 3.0E-04 | NM_001003920 | chr7 |
| Hspb1 | -0.93 | 1.7E-04 | NM_013560 | chr5 |
| Aacs | -0.93 | 1.1E-04 | NM_030210 | chr5 |
| Rbm4b | -0.93 | 8.6E-05 | NM_025717 | chr19 |
| Cntfr | -0.93 | 1.5E-04 | NM_001136056 | chr4 |
| Atp6v0e2 | -0.93 | 3.3E-05 | NM_133764 | chr6 |
| Armcx2 | -0.93 | 2.2E-04 | NM_026139 | chrX |
| Scn9a | -0.93 | 9.5E-05 | NM_018852 | chr2 |
| Ola1 | -0.93 | 1.0E-04 | NM_025942 | chr2 |
| Ttll5 | -0.93 | 9.5E-05 | NM_001081423 | chr12 |
| Rps6ka6 | -0.93 | 2.8E-04 | NM_025949 | chrX |
| Cars2 | -0.93 | 2.6E-04 | NM_024248 | chr8 |
| Atp6v1d | -0.93 | 1.0E-04 | NM_023721 | chr12 |
| Gclm | -0.93 | 6.2E-05 | NM_008129 | chr3 |
| Fam54a | -0.93 | 1.5E-04 | NM_027930 | chr10 |
| A430089I19Rik | -0.93 | 4.6E-05 | NM_177913 | chr5 |
| Scnn1g | -0.93 | 3.2E-04 | NM_011326 | chr7 |
| Renbp | -0.93 | 1.1E-04 | NM_023132 | chrX |
| Tfrc | -0.91 | 1.3E-04 | NM_011638 | chr16 |
| Mcm3ap | -0.91 | 6.5E-05 | NM_019434 | chr10 |
| Gm941 | -0.91 | 3.1E-04 | ENSMUST00000068175 | chr17 |
| Tuba1a | -0.91 | 2.2E-04 | NM_011653 | chr15 |
| Hspa8 | -0.91 | 1.5E-04 | M13967 | chr9 |
| Rbm43 | -0.91 | 3.2E-04 | NM_030243 | chr2 |
| Slc36a1 | -0.91 | 2.2E-04 | NM_153139 | chr11 |
| Ars2 | -0.91 | 9.2E-05 | NM_031405 | chr5 |
| Arid5b | -0.90 | 2.4E-04 | NM_023598 | chr10 |
| Pcyox1l | -0.90 | 2.0E-04 | NM_172832 | chr18 |
| Rufy2 | -0.90 | 3.9E-05 | NM_027425 | chr10 |
| Dmkn | -0.90 | 3.0E-04 | NM_172899 | chr7 |
| Hspa8 | -0.90 | 1.6E-04 | M13967 | chr9 |
| Pdxk | -0.90 | 3.5E-04 | NM_172134 | chr10 |
| B130055M24Rik | -0.90 | 8.6E-05 | AK041042 | chr7 |
| Hnrnph1 | -0.90 | 5.8E-05 | NM_021510 | chr11 |
| Cxcl10 | -0.89 | 2.4E-04 | NM_021274 | chr5 |
| Nsd1 | -0.89 | 1.8E-04 | NM_008739 | chr13 |
| Bspry | -0.89 | 1.8E-04 | NM_138653 | chr4 |
| Gdf11 | -0.89 | 1.3E-04 | NM_010272 | chr10 |
| A530082C11Rik | -0.89 | 6.3E-05 | NM_177186 | chr4 |
| Ttyh3 | -0.89 | 6.1E-05 | NM_175274 | chr5 |
| Tspan7 | -0.89 | 1.5E-04 | NM_019634 | chrX |
| Ccnk | -0.89 | 3.0E-04 | NM_009832 | chr12 |
| Mtrf1l | -0.89 | 7.8E-05 | NM_175374 | chr10 |
| Nav3 | -0.89 | 8.4E-05 | NM_001081035 | chr10 |
| Wasf1 | -0.89 | 3.4E-04 | NM_031877 | chr10 |
| Pigh | -0.88 | 3.9E-04 | NM_029988 | chr12 |
| Spry1 | -0.88 | 1.9E-04 | NM_011896 | chr3 |
| B230120H23Rik | -0.88 | 6.5E-05 | NM_023057 | chr2 |
| Lifr | -0.88 | 3.0E-04 | NM_013584 | chr15 |
| Bcl2a1d | -0.88 | 1.8E-04 | NM_007536 | chr9 |
| OTTMUSG00000011262 | -0.88 | 9.9E-05 | NM_001085528 | chr4 |
| Abhd3 | -0.88 | 1.6E-04 | NM_134130 | chr18 |
| Prdx2 | -0.88 | 1.4E-04 | NM_011563 | chr1 |
| EG633640 | -0.87 | 3.4E-04 | ENSMUST00000109199 | chr13 |
| Traf3ip1 | -0.87 | 1.8E-04 | NM_028718 | chr1 |
| Ptgr2 | -0.87 | 2.3E-04 | NM_029880 | chr12 |
| --- | -0.87 | 1.5E-04 | BC094563 | chr7 |
| Gnao1 | -0.87 | 5.0E-05 | NM_010308 | chr8 |
| Vps53 | -0.87 | 8.8E-05 | NM_026664 | chr11 |
| Mpp3 | -0.87 | 1.4E-04 | NM_007863 | chr11 |
| Jarid2 | -0.86 | 1.7E-04 | NM_021878 | chr13 |
| --- | -0.86 | 8.9E-05 | ENSMUST00000083903 | chr2 |
| Ccar1 | -0.86 | 1.7E-04 | NM_026201 | chr10 |
| Jam2 | -0.86 | 1.3E-04 | NM_023844 | chr16 |
| Gyltl1b | -0.85 | 1.7E-04 | NM_172670 | chr2 |
| Adam9 | -0.85 | 6.2E-05 | NM_007404 | chr8 |
| Def8 | -0.85 | 1.2E-04 | NM_054046 | chr8 |
| Nr4a3 | -0.84 | 1.9E-04 | NM_015743 | chr4 |
| --- | -0.84 | 2.4E-04 | AF357386 | chr12 |
| Spag1 | -0.84 | 4.0E-04 | NM_012031 | chr15 |
| Abcc5 | -0.84 | 1.6E-04 | NM_013790 | chr16 |
| Gata4 | -0.84 | 3.3E-04 | NM_008092 | chr14 |
| Maged1 | -0.84 | 2.5E-04 | NM_019791 | chrX |
| Tubb1 | -0.84 | 3.6E-04 | NM_001080971 | chr2 |
| Dhtkd1 | -0.84 | 2.0E-04 | NM_001081131 | chr2 |
| Prex1 | -0.83 | 1.0E-04 | NM_177782 | chr2 |
| OTTMUSG00000011262 | -0.83 | 1.4E-04 | NM_001085528 | chr4 |
| Mrpl49 | -0.83 | 2.6E-04 | NM_026246 | chr19 |
| Muc1 | -0.83 | 4.0E-04 | NM_013605 | chr3 |
| Zbtb45 | -0.83 | 1.7E-04 | NM_001024699 | chr7 |
| Set | -0.82 | 3.2E-04 | AK031561 | chr2 |
| Polr1e | -0.82 | 8.7E-05 | NM_022811 | chr4 |
| Slc26a9 | -0.82 | 1.6E-04 | NM_177243 | chr1 |
| Apip | -0.82 | 1.1E-04 | NM_019735 | chr2 |
| Phf6 | -0.81 | 2.5E-04 | NM_027642 | chrX |
| Homer2 | -0.81 | 3.4E-04 | NM_011983 | chr7 |
| --- | -0.81 | 3.7E-04 | NC_005089 | chrM |
| Mbnl3 | -0.81 | 9.4E-05 | NM_134163 | chrX |
| Akap5 | -0.81 | 3.1E-04 | NM_001101471 | chr12 |
| Hmgcr | -0.81 | 1.5E-04 | NM_008255 | chr13 |
| Pard3b | -0.80 | 1.8E-04 | NM_001081050 | chr1 |
| Sfrs17b | -0.80 | 1.5E-04 | NM_001081956 | chrX |
| Cabin1 | -0.80 | 1.9E-04 | NM_172549 | chr10 |
| 3110003A17Rik | -0.80 | 1.6E-04 | NM_028440 | chr10 |
| Dpy19l3 | -0.80 | 3.2E-04 | NM_178704 | chr7 |
| Dnajc12 | -0.80 | 1.4E-04 | NM_013888 | chr10 |
| Pip4k2b | -0.80 | 3.4E-04 | NM_054051 | chr11 |
| Atp6v1a | -0.80 | 9.5E-05 | NM_007508 | chr16 |
| Krtap17-1 | -0.80 | 9.9E-05 | NM_001099774 | chr11 |
| Zscan4c | -0.80 | 2.6E-04 | NM_001013765 | chr7 |
| Actr3b | -0.79 | 4.0E-04 | NM_001004365 | chr5 |
| Emb | -0.79 | 1.6E-04 | NM_010330 | chr13 |
| Jmjd1c | -0.79 | 3.5E-04 | BC068318 | chr10 |
| 2310044G17Rik | -0.79 | 2.9E-04 | BC026384 | chr12 |
| Slc9a6 | -0.79 | 2.0E-04 | NM_172780 | chrX |
| OTTMUSG00000011262 | -0.78 | 1.3E-04 | NM_001085528 | chr4 |
| OTTMUSG00000011262 | -0.78 | 1.3E-04 | NM_001085528 | chr4 |
| OTTMUSG00000011262 | -0.78 | 1.3E-04 | NM_001085528 | chr4 |
| OTTMUSG00000011262 | -0.78 | 1.3E-04 | NM_001085528 | chr4 |
| OTTMUSG00000011262 | -0.78 | 1.3E-04 | NM_001085528 | chr4 |
| OTTMUSG00000011262 | -0.78 | 1.3E-04 | NM_001085528 | chr4 |
| Il17rd | -0.78 | 3.5E-04 | NM_134437 | chr14 |
| Hapln1 | -0.78 | 3.6E-04 | NM_013500 | chr13 |
| Fam113a | -0.78 | 1.3E-04 | NM_178762 | chr2 |
| Pgd | -0.78 | 3.6E-04 | NM_001081274 | chr4 |
| Insig1 | -0.77 | 3.4E-04 | NM_153526 | chr5 |
| Rnasek | -0.77 | 2.1E-04 | NM_173742 | chr11 |
| --- | -0.77 | 2.3E-04 | ENSMUST00000083426 | chr7 |
| Slc38a6 | -0.77 | 1.6E-04 | BC157917 | chr12 |
| Sema5a | -0.77 | 1.9E-04 | NM_009154 | chr15 |
| 100040305 | -0.77 | 2.5E-04 | XM_001474563 | chr12 |
| Ssx2ip | -0.77 | 3.5E-04 | NM_138744 | chr3 |
| Slc39a9 | -0.76 | 1.6E-04 | BC158056 | chr12 |
| Zcchc17 | -0.76 | 3.5E-04 | NM_153160 | chr4 |
| Ints2 | -0.76 | 3.5E-04 | NM_027421 | chr11 |
| Moap1 | -0.76 | 1.3E-04 | NM_022323 | chr12 |
| Ubfd1 | -0.76 | 1.3E-04 | NM_138589 | chr7 |
| Caprin1 | -0.76 | 2.0E-04 | NM_016739 | chr2 |
| Fads2 | -0.76 | 2.0E-04 | NM_019699 | chr19 |
| Ostm1 | -0.76 | 2.2E-04 | NM_172416 | chr10 |
| Mdm2 | -0.76 | 1.4E-04 | NM_010786 | chr10 |
| Rtn2 | -0.76 | 1.9E-04 | NM_013648 | chr7 |
| Ptprn | -0.75 | 1.6E-04 | NM_008985 | chr1 |
| Tgfb3 | -0.75 | 1.3E-04 | NM_009368 | chr12 |
| Prpf40a | -0.75 | 2.9E-04 | NM_018785 | chr2 |
| Hecw2 | -0.75 | 1.1E-04 | NM_001001883 | chr1 |
| OTTMUSG00000011262 | -0.75 | 2.2E-04 | NM_001085528 | chr4 |
| Lamb1-1 | -0.74 | 3.4E-04 | NM_008482 | chr12 |
| Lrp8 | -0.74 | 1.2E-04 | NM_053073 | chr4 |
| Dnmt3b | -0.74 | 3.8E-04 | NM_001003961 | chr2 |
| Ccno | -0.74 | 2.4E-04 | NM_001081062 | chr13 |
| Tbc1d30 | -0.74 | 2.4E-04 | AK173079 | chr10 |
| Ints1 | -0.74 | 2.9E-04 | NM_026748 | chr5 |
| Ston2 | -0.74 | 2.7E-04 | NM_175367 | chr12 |
| Dhx37 | -0.74 | 3.3E-04 | NM_203319 | chr5 |
| Max | -0.74 | 2.9E-04 | NM_008558 | chr12 |
| Rgs19 | -0.74 | 2.1E-04 | NM_026446 | chr2 |
| Atp5g1 | -0.74 | 2.2E-04 | NM_007506 | chr11 |
| Atf2 | -0.74 | 3.0E-04 | NM_001025093 | chr2 |
| Cs | -0.74 | 2.0E-04 | NM_026444 | chr10 |
| Prtg | -0.73 | 3.6E-04 | NM_175485 | chr9 |
| Igsf9b | -0.73 | 2.5E-04 | NM_001129787 | chr9 |
| --- | -0.73 | 2.9E-04 | ENSMUST00000097987 | chr4 |
| --- | -0.73 | 3.2E-04 | ENSMUST00000097988 | chr4 |
| Parp1 | -0.72 | 2.2E-04 | NM_007415 | chr1 |
| Bola2 | -0.72 | 1.7E-04 | NM_175103 | chr7 |
| Slc18a1 | -0.72 | 2.7E-04 | NM_153054 | chr8 |
| Atg2b | -0.72 | 3.7E-04 | NM_029654 | chr12 |
| Sbk1 | -0.72 | 3.5E-04 | NM_145587 | chr7 |
| Aldh4a1 | -0.72 | 2.1E-04 | NM_175438 | chr4 |
| Zfp410 | -0.72 | 3.2E-04 | NM_144833 | chr12 |
| Ctsa | -0.72 | 3.2E-04 | NM_008906 | chr2 |
| Ddx24 | -0.72 | 2.1E-04 | NM_020494 | chr12 |
| Htatsf1 | -0.72 | 3.2E-04 | NM_028242 | chrX |
| 3110062M04Rik | -0.71 | 2.5E-04 | NM_199145 | chr6 |
| 2610110G12Rik | -0.71 | 1.7E-04 | BC028847 | chr17 |
| --- | -0.71 | 2.5E-04 | ENSMUST00000083913 | chr3 |
| Slc25a17 | -0.71 | 2.7E-04 | NM_011399 | chr15 |
| Cdc25c | -0.71 | 2.8E-04 | NM_009860 | chr18 |
| Atp5g1 | -0.71 | 2.6E-04 | NM_007506 | chr11 |
| Clcn6 | -0.71 | 2.1E-04 | NM_011929 | chr4 |
| Sepx1 | -0.70 | 3.2E-04 | NM_013759 | chr17 |
| Dbn1 | -0.70 | 1.7E-04 | NM_019813 | chr13 |
| Limk1 | -0.70 | 3.3E-04 | NM_010717 | chr5 |
| Psmd4 | -0.70 | 2.6E-04 | NM_008951 | chr3 |
| Trp53inp1 | -0.70 | 2.1E-04 | NM_021897 | chr4 |
| Nkx2-1 | -0.69 | 2.5E-04 | NM_009385 | chr12 |
| Ccdc45 | -0.69 | 1.8E-04 | NM_177088 | chr11 |
| Spred1 | -0.69 | 2.6E-04 | NM_033524 | chr2 |
| Nup93 | -0.69 | 2.9E-04 | NM_172410 | chr8 |
| Dhx9 | -0.69 | 2.2E-04 | NM_007842 | chr1 |
| Kitl | -0.69 | 3.3E-04 | NM_013598 | chr10 |
| 2310035C23Rik | -0.69 | 2.9E-04 | NM_173187 | chr1 |
| Stxbp4 | -0.69 | 3.4E-04 | NM_011505 | chr11 |
| Med19 | -0.68 | 3.0E-04 | NM_025885 | chr2 |
| --- | -0.68 | 3.5E-04 | ENSMUST00000097985 | chr4 |
| Tspyl2 | -0.68 | 3.2E-04 | NM_029836 | chrX |
| Stam2 | -0.68 | 2.4E-04 | NM_019667 | chr2 |
| Ank2 | -0.68 | 3.4E-04 | NM_178655 | chr3 |
| Cmtm7 | -0.68 | 3.4E-04 | NM_133978 | chr9 |
| Brd7 | -0.68 | 3.4E-04 | NM_012047 | chr8 |
| Apaf1 | -0.68 | 2.7E-04 | NM_001042558 | chr10 |
| Ube4a | -0.67 | 3.7E-04 | NM_145400 | chr9 |
| Lyrm5 | -0.67 | 2.7E-04 | NM_133688 | chr6 |
| Gtse1 | -0.67 | 2.9E-04 | NM_013882 | chr15 |
| Cdc40 | -0.67 | 4.0E-04 | NM_027879 | chr10 |
| Ppih | -0.67 | 3.3E-04 | NM_001110130 | chr11 |
| Dnaja1 // Dnaja1 | -0.66 | 2.5E-04 | BC158024 | chr5 |
| Ppih | -0.66 | 3.0E-04 | NM_028677 | chr17 |
| Trafd1 | -0.66 | 3.7E-04 | NM_172275 | chr5 |
| Pigt | -0.66 | 2.8E-04 | NM_133779 | chr2 |
| Bcas2 | -0.66 | 3.0E-04 | NM_026602 | chr3 |
| Odf2 | -0.66 | 3.0E-04 | NM_001113213 | chr2 |
| Pde8a | -0.65 | 2.8E-04 | NM_008803 | chr7 |
| Ncbp2 | -0.65 | 3.1E-04 | NM_026554 | chr16 |
| Atp6v0a2 | -0.65 | 3.4E-04 | NM_011596 | chr5 |
| Cklf | -0.65 | 3.4E-04 | NM_001037841 | chr8 |
| Spag5 | -0.65 | 3.9E-04 | NM_017407 | chr11 |
| Ppih | -0.65 | 3.5E-04 | NM_028677 | chr5 |
| Pof1b | -0.65 | 2.9E-04 | NM_181579 | chrX |
| Tob2 | -0.65 | 3.6E-04 | NM_020507 | chr15 |
| AU014645 | -0.64 | 3.6E-04 | NM_001033201 | chr4 |
| Zfyve26 | -0.63 | 3.6E-04 | NM_001008550 | chr12 |
| --- | -0.63 | 3.9E-04 | AK158967 | chr11 |
| 6330439K17Rik | -0.63 | 3.9E-04 | NM_172859 | chr2 |
| Slc4a11 | -0.60 | 4.0E-04 | NM_001081162 | chr2 |
| Tsc22d3 | 0.63 | 3.3E-04 | NM_001077364 | chrX |
| Ppp1cb | 0.64 | 3.6E-04 | NM_172707 | chr5 |
| 5430407P10Rik | 0.64 | 2.9E-04 | BC025867 | chr2 |
| Ccdc50 | 0.64 | 4.0E-04 | NM_026202 | chr16 |
| Aldh3a1 | 0.65 | 3.6E-04 | NM_007436 | chr11 |
| --- | 0.65 | 2.8E-04 | ENSMUST00000085626 | chr12 |
| Iqwd1 | 0.65 | 3.8E-04 | BC084732 | chr1 |
| Tle4 | 0.66 | 3.2E-04 | NM_011600 | chr19 |
| Asb3 | 0.66 | 3.4E-04 | NM_023906 | chr11 |
| Ndel1 | 0.67 | 2.7E-04 | NM_023668 | chr11 |
| Fbxl4 | 0.67 | 4.0E-04 | NM_172988 | chr4 |
| Fam83h | 0.68 | 3.8E-04 | BC117947 | chr15 |
| --- | 0.68 | 3.2E-04 | mmu-mir-27a // mmu-mir-27a | chr8 |
| Tbc1d2 | 0.68 | 2.5E-04 | NM_198664 | chr4 |
| Itfg1 | 0.68 | 3.8E-04 | NM_028007 | chr8 |
| St3gal4 | 0.68 | 3.5E-04 | NM_009178 | chr9 |
| Ltbp4 | 0.69 | 4.0E-04 | NM_175641 | chr7 |
| Phf17 | 0.69 | 2.9E-04 | NM_172303 | chr3 |
| 38967 | 0.69 | 2.3E-04 | NM_033144 | chr11 |
| Cars | 0.69 | 2.3E-04 | NM_013742 | chr7 |
| Mras | 0.69 | 3.3E-04 | NM_008624 | chr9 |
| Coq10b | 0.70 | 3.9E-04 | NM_001039710 | chr1 |
| Cd99l2 | 0.70 | 2.2E-04 | NM_138309 | chrX |
| Xpot | 0.70 | 3.0E-04 | NM_001081056 | chr10 |
| Il4ra | 0.70 | 3.6E-04 | NM_001008700 | chr7 |
| Gata1 | 0.70 | 2.3E-04 | NM_008089 | chrX |
| --- | 0.70 | 2.0E-04 | ENSMUST00000076828 | chr1 |
| Ddit3 | 0.70 | 3.7E-04 | NM_007837 | chr10 |
| Smad3 | 0.71 | 2.9E-04 | NM_016769 | chr9 |
| Mtm1 | 0.71 | 2.7E-04 | NM_019926 | chrX |
| Tgfa | 0.71 | 2.0E-04 | NM_031199 | chr6 |
| Flrt3 | 0.71 | 1.9E-04 | NM_178382 | chr2 |
| Itgb1 | 0.72 | 2.0E-04 | NM_010578 | chr8 |
| Adam10 | 0.72 | 2.9E-04 | NM_007399 | chr9 |
| Mpzl2 | 0.72 | 2.9E-04 | NM_007962 | chr9 |
| Snx33 | 0.72 | 2.3E-04 | NM_175483 | chr9 |
| EG434459 | 0.72 | 3.7E-04 | NM_001013816 | chr14 |
| Ube3c | 0.72 | 2.4E-04 | NM_133907 | chr5 |
| BC004728 | 0.72 | 3.9E-04 | NM_174992 | chr15 |
| Myo7a | 0.72 | 3.9E-04 | NM_008663 | chr7 |
| H2-M3 | 0.72 | 2.3E-04 | NM_013819 | chr17 |
| Aldoa | 0.72 | 3.4E-04 | NM_007438 | chr7 |
| Otub2 | 0.72 | 3.2E-04 | NM_026580 | chr12 |
| Arhgap22 | 0.72 | 1.9E-04 | NM_153800 | chr14 |
| Map3k3 | 0.72 | 2.7E-04 | NM_011947 | chr11 |
| Naaa | 0.73 | 1.5E-04 | NM_025972 | chr5 |
| Lrrc8a | 0.73 | 2.3E-04 | NM_177725 | chr2 |
| Atpgd1 | 0.73 | 2.2E-04 | BC023699 | chr19 |
| Efnb2 | 0.74 | 2.0E-04 | NM_010111 | chr8 |
| Zdhhc2 | 0.74 | 1.5E-04 | NM_178395 | chr8 |
| Katnal1 | 0.74 | 2.9E-04 | NM_153572 | chr5 |
| Bnip3l | 0.74 | 4.0E-04 | NM_009761 | chr14 |
| Vgll3 | 0.74 | 3.3E-04 | AK007165 | chr16 |
| Rtkn | 0.74 | 2.3E-04 | NM_133641 | chr6 |
| Pgm1 | 0.74 | 1.9E-04 | NM_025700 | chr5 |
| Fbxw4 | 0.74 | 2.5E-04 | NM_013907 | chr19 |
| Stard6 | 0.74 | 2.0E-04 | NM_029019 | chr18 |
| Phf10 | 0.75 | 1.2E-04 | NM_024250 | chr17 |
| Npnt | 0.75 | 1.6E-04 | NM_033525 | chr3 |
| Myd116 | 0.75 | 2.5E-04 | NM_008654 | chr7 |
| Txnrd3 | 0.75 | 2.9E-04 | NM_153162 | chr6 |
| Rin1 | 0.75 | 1.2E-04 | NM_145495 | chr19 |
| 4933434I06Rik | 0.76 | 1.3E-04 | NM_027728 | chr2 |
| Rnf11 | 0.76 | 1.9E-04 | NM_013876 | chr4 |
| Map4k3 | 0.76 | 2.0E-04 | NM_001081357 | chr17 |
| Tagln2 | 0.76 | 3.0E-04 | NM_178598 | chr1 |
| Rarg | 0.76 | 3.4E-04 | NM_011244 | chr15 |
| 0610010F05Rik | 0.76 | 3.9E-04 | NM_027860 | chr11 |
| Garnl1 | 0.76 | 2.5E-04 | NM_001112714 | chr12 |
| Lmna | 0.76 | 1.8E-04 | NM_001002011 | chr3 |
| Prmt7 | 0.77 | 3.2E-04 | NM_145404 | chr8 |
| Atp10a | 0.77 | 1.6E-04 | NM_009728 | chr7 |
| Vmn2r96 | 0.77 | 2.7E-04 | NM_001104547 | chr17 |
| Lamb2 | 0.77 | 1.0E-04 | NM_008483 | chr9 |
| Nrbp1 | 0.78 | 1.6E-04 | NM_147201 | chr5 |
| Myo1c | 0.78 | 1.0E-04 | NM_008659 | chr11 |
| Atg9b | 0.78 | 1.6E-04 | NM_001002897 | chr5 |
| Whsc1 | 0.78 | 1.8E-04 | NM_001081102 | chr5 |
| Igsf9 | 0.78 | 1.3E-04 | NM_033608 | chr1 |
| Ephb3 | 0.78 | 3.4E-04 | NM_010143 | chr16 |
| Arhgap9 | 0.78 | 1.0E-04 | NM_146011 | chr10 |
| Nop14 | 0.78 | 1.6E-04 | NM_029278 | chr5 |
| Ermp1 | 0.78 | 1.9E-04 | NM_001081213 | chr19 |
| Mitf | 0.79 | 1.1E-04 | NM_001113198 | chr6 |
| Tmem168 | 0.79 | 3.1E-04 | NM_028990 | chr6 |
| Kctd15 | 0.79 | 1.7E-04 | NM_146188 | chr7 |
| Gcom1 | 0.79 | 3.6E-04 | NM_001033208 | chr9 |
| Asah2 | 0.79 | 1.6E-04 | NM_018830 | chr19 |
| Kirrel | 0.79 | 1.6E-04 | NM_130867 | chr3 |
| Foxc1 | 0.79 | 1.4E-04 | NM_008592 | chr13 |
| Zfp422 | 0.79 | 1.7E-04 | BC018339 | chr17 |
| BC023744 | 0.79 | 2.2E-04 | NM_001033311 | chr5 |
| D1Ertd622e | 0.79 | 1.2E-04 | BC023951 | chr1 |
| Nfya | 0.79 | 3.2E-04 | NM_001110832 | chr17 |
| Rell1 | 0.79 | 1.4E-04 | NM_145923 | chr5 |
| Tapt1 | 0.79 | 1.3E-04 | NM_173764 | chr5 |
| Klk10 | 0.79 | 1.2E-04 | NM_133712 | chr7 |
| Coq2 | 0.80 | 1.6E-04 | NM_027978 | chr5 |
| LOC675799 | 0.80 | 1.6E-04 | XR_032694 | chr7 |
| Gramd3 | 0.80 | 2.0E-04 | NM_026240 | chr18 |
| Itpr3 | 0.80 | 8.6E-05 | NM_080553 | chr17 |
| Flt1 | 0.80 | 2.3E-04 | NM_010228 | chr5 |
| Ppm2c | 0.80 | 1.9E-04 | NM_001098230 | chr4 |
| Gcnt2 | 0.80 | 3.5E-04 | NM_023887 | chr13 |
| Skil | 0.80 | 9.5E-05 | NM_011386 | chr3 |
| Ttc7 | 0.80 | 1.1E-04 | NM_028639 | chr17 |
| Chst1 | 0.80 | 3.0E-04 | NM_023850 | chr2 |
| Ltbp3 | 0.80 | 1.6E-04 | NM_008520 | chr19 |
| Unc13d | 0.80 | 2.9E-04 | NM_001009573 | chr11 |
| Kcnab1 | 0.81 | 1.8E-04 | NM_010597 | chr3 |
| Sema7a | 0.81 | 1.3E-04 | NM_011352 | chr9 |
| Pgm5 | 0.81 | 1.9E-04 | NM_175013 | chr19 |
| Zic5 | 0.81 | 1.4E-04 | AK006747 | chr11 |
| Cd55 | 0.81 | 2.5E-04 | NM_010016 | chr1 |
| Stard10 | 0.81 | 2.1E-04 | NM_019990 | chr7 |
| Cdh2 | 0.81 | 1.7E-04 | NM_007664 | chr18 |
| Lars | 0.81 | 1.1E-04 | NM_134137 | chr18 |
| Cebpg | 0.81 | 2.1E-04 | NM_009884 | chr7 |
| Phgdh | 0.81 | 1.8E-04 | NM_016966 | chr7 |
| H60b | 0.82 | 3.7E-04 | AB284505 | chr10 |
| Snx9 | 0.82 | 1.6E-04 | NM_025664 | chr17 |
| 1700019H03Rik | 0.82 | 1.3E-04 | NM_183161 | chr2 |
| Cxcl16 | 0.82 | 1.4E-04 | NM_023158 | chr11 |
| Zfyve9 | 0.82 | 1.9E-04 | NM_183300 | chr4 |
| Lpar1 | 0.82 | 2.2E-04 | NM_010336 | chr4 |
| Ptpn12 | 0.82 | 1.0E-04 | NM_011203 | chr5 |
| Stard6 | 0.82 | 1.8E-04 | BC087893 | chr10 |
| Atf6 | 0.82 | 1.3E-04 | NM_001081304 | chr1 |
| Prss23 | 0.83 | 1.1E-04 | NM_029614 | chr7 |
| Cobll1 | 0.83 | 3.7E-04 | NM_177025 | chr2 |
| Ndufa9 | 0.83 | 2.2E-04 | NM_025358 | chr6 |
| Cyb5r3 | 0.83 | 3.7E-04 | NM_029787 | chr15 |
| Pi4k2b | 0.83 | 1.6E-04 | NM_028744 | chr5 |
| Dusp7 | 0.83 | 2.3E-04 | NM_153459 | chr9 |
| Atp13a3 | 0.83 | 1.2E-04 | NM_001128096 | chr16 |
| ENSMUSG00000074917 | 0.83 | 1.6E-04 | ENSMUST00000099550 | chr13 |
| D0H4S114 | 0.84 | 2.0E-04 | NM_053078 | chr18 |
| Ccdc68 | 0.84 | 1.8E-04 | NM_201362 | chr18 |
| Nfil3 | 0.84 | 1.8E-04 | NM_017373 | chr13 |
| Ank | 0.84 | 1.4E-04 | NM_020332 | chr15 |
| Pask | 0.84 | 2.1E-04 | NM_080850 | chr1 |
| D5Ertd579e | 0.84 | 2.0E-04 | NM_001081232 | chr5 |
| Rad18 | 0.84 | 8.4E-05 | NM_021385 | chr6 |
| Elmo3 | 0.84 | 1.4E-04 | NM_172760 | chr8 |
| Sema4c | 0.84 | 1.8E-04 | NM_001126047 | chr1 |
| Leprotl1 | 0.84 | 7.9E-05 | NM_026609 | chr8 |
| Nedd9 | 0.85 | 1.6E-04 | NM_001111324 | chr13 |
| Dbp | 0.85 | 2.8E-04 | NM_016974 | chr7 |
| Cdc25b | 0.85 | 1.3E-04 | NM_023117 | chr2 |
| Map3k2 | 0.85 | 1.1E-04 | NM_011946 | chr18 |
| Atp1b1 | 0.85 | 1.0E-04 | NM_009721 | chr1 |
| Insr | 0.85 | 1.2E-04 | NM_010568 | chr8 |
| Eef2k | 0.85 | 2.0E-04 | NM_007908 | chr7 |
| Timp2 | 0.85 | 9.1E-05 | NM_011594 | chr11 |
| Kif5c | 0.85 | 1.5E-04 | NM_008449 | chr2 |
| L1cam | 0.85 | 2.8E-04 | NM_008478 | chrX |
| Tnfrsf1a | 0.85 | 2.5E-04 | NM_011609 | chr6 |
| Ppm1j | 0.85 | 1.5E-04 | NM_027982 | chr3 |
| Sema4b | 0.85 | 6.3E-05 | NM_013659 | chr7 |
| Plekha6 | 0.85 | 3.0E-04 | NM_182930 | chr1 |
| Nudt4 | 0.85 | 7.9E-05 | NM_027722 | chr10 |
| Hr | 0.85 | 1.1E-04 | NM_021877 | chr14 |
| Rnf13 | 0.86 | 3.9E-04 | NM_001113413 | chr3 |
| Klf10 | 0.86 | 1.5E-04 | NM_013692 | chr15 |
| Mmp14 | 0.86 | 3.2E-04 | NM_008608 | chr14 |
| Atf3 | 0.86 | 6.4E-05 | NM_007498 | chr1 |
| Adss | 0.86 | 2.1E-04 | NM_007422 | chr1 |
| Pcyt1b | 0.86 | 3.3E-04 | NM_211138 | chrX |
| Il15 | 0.86 | 3.1E-04 | NM_008357 | chr8 |
| Mupcdh | 0.86 | 3.3E-04 | NM_001114322 | chr7 |
| Tmem43 | 0.86 | 1.5E-04 | NM_028766 | chr6 |
| Itgb4 | 0.86 | 9.8E-05 | NM_001005608 | chr11 |
| Rnf19a | 0.87 | 5.2E-05 | NM_013923 | chr15 |
| Fbxo31 | 0.87 | 1.6E-04 | NM_133765 | chr8 |
| Unc84b | 0.87 | 3.6E-04 | NM_194342 | chr15 |
| Sned1 | 0.87 | 1.9E-04 | NM_172463 | chr1 |
| Sbds | 0.87 | 2.7E-04 | NM_023248 | chr5 |
| Srgap1 | 0.87 | 1.6E-04 | NM_001081037 | chr10 |
| B3gnt2 | 0.87 | 6.1E-05 | NM_016888 | chr11 |
| Reep6 | 0.87 | 8.1E-05 | NM_139292 | chr10 |
| Lims2 | 0.87 | 6.5E-05 | NM_144862 | chr18 |
| Ulk4 | 0.87 | 3.7E-04 | BC109365 | chr9 |
| Nfe2l1 | 0.87 | 6.3E-05 | NM_008686 | chr11 |
| Cadm4 | 0.87 | 9.5E-05 | NM_153112 | chr7 |
| Sfxn3 | 0.87 | 1.6E-04 | NM_053197 | chr19 |
| Gm129 | 0.88 | 8.5E-05 | BC132471 | chr3 |
| Rdh10 | 0.88 | 3.2E-04 | NM_133832 | chr1 |
| Sorcs2 | 0.88 | 6.2E-05 | NM_030889 | chr5 |
| Sh3tc1 | 0.88 | 9.6E-05 | NM_194344 | chr5 |
| AI316807 | 0.88 | 1.1E-04 | BC048089 | chr8 |
| Iffo2 | 0.88 | 2.1E-04 | NM_183148 | chr4 |
| Bre | 0.88 | 1.3E-04 | NM_181279 | chr5 |
| Elk3 | 0.88 | 2.6E-04 | NM_013508 | chr10 |
| Sumf1 | 0.88 | 2.5E-04 | NM_145937 | chr6 |
| Sgcb | 0.89 | 1.1E-04 | NM_011890 | chr5 |
| Speer1-ps1 | 0.89 | 1.7E-04 | NR_001586 | chr5 |
| Itga3 | 0.89 | 4.5E-05 | NM_013565 | chr11 |
| Anxa5 | 0.89 | 1.1E-04 | NM_009673 | chr3 |
| Large | 0.89 | 3.7E-04 | NM_010687 | chr8 |
| Scarb2 | 0.89 | 1.7E-04 | NM_007644 | chr5 |
| 1300018J18Rik | 0.89 | 9.6E-05 | NM_027905 | chr15 |
| --- | 0.89 | 4.0E-04 | ENSMUST00000100696 | chr14 |
| AI607873 | 0.89 | 1.1E-04 | BC150711 | chr1 |
| Itga2 | 0.90 | 7.8E-05 | NM_008396 | chr13 |
| Tes | 0.90 | 8.4E-05 | NM_207176 | chr6 |
| Litaf | 0.90 | 3.0E-04 | NM_019980 | chr16 |
| Cmas | 0.90 | 5.2E-05 | NM_009908 | chr6 |
| Arpc2 | 0.90 | 2.5E-04 | NM_029711 | chr1 |
| Farp1 | 0.90 | 3.1E-04 | NM_134082 | chr14 |
| F2rl1 | 0.90 | 1.9E-04 | NM_007974 | chr13 |
| 2700089E24Rik | 0.90 | 6.7E-05 | BC053418 | chr6 |
| Zfp238 | 0.91 | 4.2E-05 | NM_001012330 | chr1 |
| Fam152a | 0.91 | 5.4E-05 | NM_024282 | chr1 |
| Sytl2 | 0.91 | 2.2E-04 | NM_001040085 | chr7 |
| Pnpla8 | 0.91 | 1.8E-04 | NM_026164 | chr12 |
| Anxa2 | 0.91 | 7.5E-05 | NM_007585 | chr9 |
| Akap2 | 0.91 | 2.7E-04 | NM_001035533 | chr4 |
| Rdm1 | 0.91 | 4.0E-04 | NM_025654 | chr11 |
| Rhoc | 0.91 | 2.7E-04 | NM_007484 | chr3 |
| Klf7 | 0.91 | 3.4E-04 | NM_033563 | chr1 |
| Cyp4f40 | 0.92 | 6.7E-05 | NM_001101588 | chr17 |
| Cpeb2 | 0.92 | 4.5E-05 | NM_175937 | chr5 |
| Psme2 | 0.92 | 2.8E-04 | NM_011190 | chr11 |
| Anxa11 | 0.92 | 7.3E-05 | NM_013469 | chr14 |
| Ulk2 | 0.92 | 9.5E-05 | NM_013881 | chr11 |
| Ppp3cc | 0.92 | 1.2E-04 | NM_008915 | chr14 |
| Lonp1 | 0.92 | 8.7E-05 | NM_028782 | chr17 |
| Areg | 0.92 | 2.7E-04 | NM_009704 | chr5 |
| EG623849 | 0.93 | 1.3E-04 | NR_003596 | chr5 |
| ENSMUSG00000074294 | 0.93 | 1.3E-04 | ENSMUST00000098707 | chr8 |
| Adcy9 | 0.93 | 1.1E-04 | NM_009624 | chr16 |
| Smpd3 | 0.93 | 1.8E-04 | NM_021491 | chr8 |
| Gpr19 | 0.93 | 8.6E-05 | NM_008157 | chr6 |
| --- | 0.93 | 2.2E-04 | ENSMUST00000120217 | chr2 |
| Tnfsf9 | 0.93 | 1.1E-04 | NM_009404 | chr17 |
| Dppa2 | 0.94 | 2.9E-04 | NM_028615 | chr16 |
| AI847670 | 0.94 | 3.4E-04 | NM_177869 | chr5 |
| Antxr2 | 0.94 | 5.1E-05 | NM_133738 | chr5 |
| Soat2 | 0.94 | 3.0E-04 | NM_146064 | chr15 |
| Kcnu1 | 0.94 | 3.5E-05 | NM_008432 | chr8 |
| 1110003E01Rik | 0.94 | 1.5E-04 | BC009097 | chr5 |
| Dapk2 | 0.94 | 1.6E-04 | NM_010019 | chr9 |
| Angptl6 | 0.94 | 2.2E-04 | NM_145154 | chr9 |
| Ly6g6c | 0.94 | 1.4E-04 | NM_023463 | chr17 |
| Plec1 | 0.94 | 8.4E-05 | NM_011117 | chr15 |
| Gprc5c | 0.94 | 2.8E-04 | NM_001110337 | chr11 |
| Tfdp2 | 0.94 | 1.6E-04 | NM_178667 | chr9 |
| Eps8 | 0.95 | 6.5E-05 | NM_007945 | chr6 |
| Maml2 | 0.95 | 2.7E-04 | NM_001013813 | chr9 |
| Myc | 0.95 | 8.6E-05 | NM_010849 | chr15 |
| Hipk2 | 0.95 | 2.0E-04 | NM_010433 | chr6 |
| Sdc1 | 0.95 | 8.2E-05 | NM_011519 | chr12 |
| Fam162a | 0.96 | 3.2E-04 | NM_027342 | chr16 |
| Mthfd2 | 0.96 | 3.1E-04 | NM_008638 | chr6 |
| 2010300C02Rik | 0.96 | 1.8E-04 | BC072639 | chr1 |
| Narf | 0.96 | 3.4E-04 | NM_026272 | chr11 |
| Manea | 0.96 | 1.6E-04 | NM_172865 | chr4 |
| --- | 0.96 | 3.1E-04 | BC099691 | chr16 |
| Palm2 | 0.97 | 2.2E-04 | NM_172868 | chr4 |
| Atp2b1 | 0.97 | 7.1E-05 | NM_026482 | chr10 |
| Sil1 | 0.97 | 2.8E-05 | NM_030749 | chr18 |
| Tiparp | 0.97 | 1.5E-04 | NM_178892 | chr3 |
| Ghitm | 0.97 | 3.4E-05 | NM_078478 | chr14 |
| Mxd1 | 0.97 | 8.4E-05 | NM_010751 | chr6 |
| Dysf | 0.97 | 5.8E-05 | NM_021469 | chr6 |
| Gpr137b-ps | 0.97 | 7.6E-05 | NR_003568 | chr13 |
| Arl14 | 0.97 | 1.5E-04 | BC104368 | chr3 |
| Prkar2b | 0.97 | 2.9E-04 | NM_011158 | chr12 |
| Trib2 | 0.98 | 1.3E-04 | NM_144551 | chr12 |
| Stard3nl | 0.98 | 1.8E-04 | NM_024270 | chr13 |
| A230107C01Rik | 0.98 | 2.0E-04 | BC022688 | chr7 |
| Als2cl | 0.98 | 9.1E-05 | NM_146228 | chr9 |
| Myo1d | 0.98 | 3.7E-04 | NM_177390 | chr11 |
| Jhdm1d | 0.98 | 3.4E-04 | NM_001033430 | chr6 |
| Dennd2d | 0.98 | 1.3E-04 | NM_001093754 | chr3 |
| Eif4ebp1 | 0.98 | 4.0E-04 | NM_007918 | chr8 |
| Galnt11 | 0.99 | 4.7E-05 | NM_144908 | chr5 |
| Cept1 | 0.99 | 2.9E-04 | NM_133869 | chr3 |
| Ano1 | 0.99 | 3.6E-04 | NM_178642 | chr7 |
| Egln1 | 0.99 | 1.8E-04 | NM_053207 | chr8 |
| Insig2 | 0.99 | 2.7E-04 | NM_133748 | chr1 |
| Fut2 | 1.00 | 4.9E-05 | NM_018876 | chr7 |
| Zadh2 | 1.00 | 3.1E-05 | NM_146090 | chr18 |
| Ano9 | 1.00 | 1.2E-04 | NM_178381 | chr7 |
| A930001N09Rik | 1.00 | 2.6E-05 | BC113191 | chr17 |
| Mgat4a | 1.00 | 5.3E-05 | NM_173870 | chr1 |
| Fndc3a | 1.01 | 1.4E-04 | NM_207636 | chr14 |
| Plxna2 | 1.01 | 1.3E-04 | NM_008882 | chr1 |
| Sertad2 | 1.01 | 2.8E-05 | NM_021372 | chr11 |
| Tgif1 | 1.02 | 3.0E-05 | NM_009372 | chr17 |
| Sap30 | 1.02 | 3.8E-05 | NM_021788 | chr8 |
| 1110067D22Rik | 1.02 | 1.9E-05 | NM_173752 | chr11 |
| Lama3 | 1.02 | 3.6E-05 | X84014 | chr18 |
| EG433024 | 1.02 | 6.1E-05 | AK135986 | chr16 |
| Bok | 1.02 | 7.4E-05 | NM_016778 | chr1 |
| Dppa2 // Dppa2 | 1.02 | 4.3E-05 | BC137768 | chr19 |
| Tns3 | 1.02 | 3.7E-05 | NM_001083587 | chr11 |
| Met | 1.02 | 1.6E-04 | NM_008591 | chr6 |
| Cdsn | 1.02 | 3.4E-04 | NM_001008424 | chr17 |
| EG623849 | 1.02 | 3.8E-05 | NR_003596 | chr5 |
| Capn2 | 1.02 | 1.4E-04 | NM_009794 | chr1 |
| Pgap1 | 1.02 | 3.6E-04 | ENSMUST00000097739 | chr1 |
| Plscr1 | 1.03 | 2.5E-04 | NM_011636 | chr9 |
| Immp2l | 1.03 | 3.3E-04 | NM_053122 | chr12 |
| Fbxl20 | 1.03 | 4.5E-05 | NM_028149 | chr11 |
| EG623849 | 1.03 | 4.6E-05 | NR_003596 | chr5 |
| Il33 | 1.03 | 1.4E-04 | NM_133775 | chr19 |
| Arfgef1 | 1.03 | 1.4E-04 | NM_001102430 | chr1 |
| Cldn1 | 1.03 | 3.0E-04 | NM_016674 | chr16 |
| Slc7a2 | 1.04 | 4.4E-05 | NM_007514 | chr8 |
| Smox | 1.04 | 4.4E-05 | NM_145533 | chr2 |
| Sepp1 | 1.04 | 7.3E-05 | NM_009155 | chr15 |
| Trim47 | 1.04 | 7.4E-05 | NM_172570 | chr11 |
| Metrnl | 1.04 | 1.8E-04 | NM_144797 | chr11 |
| Dpy19l1 | 1.04 | 2.7E-05 | NM_172920 | chr9 |
| Klhl5 | 1.04 | 3.0E-05 | NM_175174 | chr5 |
| Ngef | 1.04 | 3.1E-05 | NM_001111314 | chr1 |
| Heg1 | 1.04 | 4.4E-05 | NM_175256 | chr16 |
| --- | 1.05 | 2.7E-04 | ENSMUST00000093877 | chr13 |
| Stim2 | 1.05 | 8.0E-05 | NM_001081103 | chr5 |
| Mmp15 | 1.05 | 2.3E-04 | NM_008609 | chr8 |
| Fermt1 | 1.05 | 3.7E-05 | NM_198029 | chr2 |
| Flnb | 1.05 | 4.5E-05 | NM_134080 | chr14 |
| 2310046K01Rik | 1.05 | 2.8E-05 | BC016127 | chr2 |
| Anxa6 | 1.05 | 1.4E-04 | NM_013472 | chr11 |
| Padi4 | 1.05 | 7.3E-05 | NM_011061 | chr4 |
| Dsg2 | 1.05 | 4.4E-05 | NM_007883 | chr18 |
| Stambpl1 | 1.05 | 2.6E-05 | NM_029682 | chr19 |
| Ptk2b | 1.06 | 3.1E-05 | NM_172498 | chr14 |
| Cyp2j6 | 1.06 | 2.8E-04 | NM_010008 | chr4 |
| Il18r1 | 1.06 | 5.5E-05 | NM_008365 | chr1 |
| Slc35f5 | 1.06 | 2.9E-05 | NM_028787 | chr1 |
| Rgs3 | 1.07 | 9.5E-05 | BC033449 | chrX |
| C630004H02Rik | 1.07 | 2.9E-05 | BC024617 | chr11 |
| Cobl | 1.07 | 4.0E-05 | NM_172496 | chr11 |
| Pyroxd1 | 1.07 | 1.5E-04 | NM_183165 | chr6 |
| Slc7a11 | 1.07 | 2.6E-05 | NM_011990 | chr3 |
| Tmcc3 | 1.07 | 3.7E-05 | NM_172051 | chr10 |
| Kif21b | 1.07 | 4.7E-05 | NM_001039472 | chr1 |
| Abtb2 | 1.07 | 1.1E-04 | NM_178890 | chr2 |
| Mxi1 | 1.07 | 3.6E-05 | NM_010847 | chr19 |
| Palm2-akap2 | 1.07 | 1.8E-04 | AF064781 | chr4 |
| Limk2 | 1.07 | 3.7E-05 | NM_010718 | chr11 |
| Macrod1 | 1.08 | 2.2E-04 | NM_134147 | chr19 |
| Crebl2 | 1.08 | 6.5E-05 | NM_177687 | chr6 |
| Sgms2 | 1.08 | 1.5E-04 | NM_028943 | chr3 |
| C130026I21Rik | 1.08 | 9.5E-05 | NM_175219 | chr1 |
| Krt20 | 1.08 | 2.4E-04 | NM_023256 | chr11 |
| B4galt6 | 1.08 | 5.0E-05 | NM_019737 | chr18 |
| Insr | 1.08 | 1.2E-04 | NM_010568 | chr8 |
| Pck2 | 1.08 | 1.4E-04 | NM_028994 | chr14 |
| Pparg | 1.08 | 5.0E-05 | NM_001127330 | chr6 |
| OTTMUSG00000007521 | 1.08 | 4.4E-05 | ENSMUST00000074198 | chr11 |
| Pacrg | 1.09 | 5.7E-05 | NM_027032 | chr17 |
| Tmtc2 | 1.09 | 2.5E-04 | NM_177368 | chr10 |
| A530032D15Rik | 1.09 | 1.6E-04 | BC094285 | chr1 |
| Ikbke | 1.09 | 6.8E-05 | NM_019777 | chr1 |
| Trpm4 | 1.09 | 5.6E-05 | NM_175130 | chr7 |
| Naaladl2 | 1.09 | 5.0E-05 | ENSMUST00000099184 | chr3 |
| Gpr85 | 1.09 | 2.9E-04 | NM_145066 | chr6 |
| Pdk1 | 1.10 | 3.5E-04 | NM_172665 | chr2 |
| Dqx1 | 1.10 | 1.9E-05 | NM_033606 | chr6 |
| Nfkbia | 1.10 | 9.5E-05 | NM_010907 | chr12 |
| Id2 | 1.10 | 4.2E-05 | NM_010496 | chr12 |
| Nebl | 1.11 | 1.4E-04 | NM_028757 | chr2 |
| Tgm1 | 1.11 | 2.6E-05 | NM_019984 | chr14 |
| Fos | 1.11 | 9.1E-05 | NM_010234 | chr12 |
| Plxnd1 | 1.11 | 1.4E-04 | NM_026376 | chr6 |
| Vasn | 1.11 | 2.4E-04 | NM_139307 | chr16 |
| Lgals3 | 1.11 | 4.0E-05 | NM_010705 | chr14 |
| Glul | 1.11 | 2.9E-05 | BC015086 | chr11 |
| Slc4a4 | 1.12 | 1.5E-04 | NM_018760 | chr5 |
| Sft2d2 | 1.12 | 7.0E-05 | NM_145512 | chr1 |
| Steap3 | 1.12 | 3.1E-05 | NM_001085409 | chr1 |
| 4930523C07Rik | 1.12 | 2.2E-05 | ENSMUST00000060298 | chr1 |
| Slc1a4 | 1.12 | 8.4E-05 | NM_018861 | chr11 |
| Grit | 1.12 | 1.8E-04 | NM_177379 | chr9 |
| Hbp1 | 1.13 | 4.3E-05 | NM_153198 | chr12 |
| Kcnq4 | 1.13 | 9.5E-05 | NM_001081142 | chr4 |
| Ypel3 | 1.13 | 2.9E-04 | NM_026875 | chr7 |
| Mlph | 1.13 | 2.6E-05 | NM_053015 | chr1 |
| ENSMUSG00000072602 | 1.13 | 5.6E-05 | NM_001098269 | chr14 |
| Mfsd6 | 1.13 | 1.1E-04 | NM_133829 | chr1 |
| EG623849 | 1.13 | 1.9E-05 | NR_003596 | chr5 |
| Spire1 | 1.14 | 8.3E-05 | NM_194355 | chr18 |
| Jhdm1d | 1.14 | 8.2E-05 | NM_001033430 | chr6 |
| Adap1 | 1.14 | 8.4E-05 | NM_172723 | chr5 |
| Edn1 | 1.15 | 2.7E-04 | NM_010104 | chr13 |
| Tns1 | 1.15 | 2.6E-05 | ENSMUST00000050681 | chr1 |
| Akr1c14 | 1.15 | 3.5E-04 | NM_134072 | chr13 |
| Zc3h6 | 1.15 | 3.0E-04 | NM_178404 | chr2 |
| Fggy | 1.15 | 2.5E-04 | NM_001113412 | chr4 |
| Eea1 | 1.15 | 4.0E-05 | NM_001001932 | chr10 |
| Birc1f | 1.15 | 6.9E-05 | NM_010871 | chr13 |
| Dusp16 | 1.16 | 5.9E-05 | NM_130447 | chr6 |
| Furin | 1.16 | 1.5E-05 | NM_011046 | chr7 |
| Hpcal4 | 1.16 | 3.1E-05 | NM_174998 | chr4 |
| Fbxl5 | 1.16 | 1.0E-05 | NM_178729 | chr5 |
| Serpinb6a | 1.17 | 3.9E-05 | NM_009254 | chr13 |
| Fn1 | 1.17 | 9.9E-05 | NM_010233 | chr1 |
| Tns1 | 1.18 | 7.7E-05 | BC055076 | chr1 |
| BC016579 | 1.18 | 2.2E-04 | BC016579 | chr16 |
| Selenbp2 | 1.18 | 4.2E-05 | NM_019414 | chr3 |
| Rras2 | 1.18 | 2.1E-04 | NM_025846 | chr7 |
| --- | 1.19 | 3.5E-04 | ENSMUST00000099164 | chr3 |
| Fggy | 1.19 | 1.9E-04 | NM_029347 | chr4 |
| Glce | 1.19 | 5.7E-05 | NM_033320 | chr9 |
| Mtap2 | 1.19 | 4.2E-05 | NM_001039934 | chr1 |
| Slc45a3 | 1.19 | 4.3E-05 | NM_145977 | chr1 |
| Krt19 | 1.19 | 2.9E-04 | NM_008471 | chr11 |
| St3gal1 | 1.19 | 9.5E-05 | NM_009177 | chr15 |
| Nr1d1 | 1.20 | 1.0E-04 | NM_145434 | chr11 |
| OTTMUSG00000011097 // OTTMUSG00000011097 // OTTMUSG00000011097 | 1.20 | 2.7E-05 | NM_001085522 | chr4 |
| Slc40a1 | 1.20 | 6.0E-05 | NM_016917 | chr1 |
| Camk1 | 1.20 | 1.9E-05 | NM_133926 | chr6 |
| Dmbt1 | 1.20 | 5.7E-05 | NM_007769 | chr7 |
| --- | 1.20 | 1.2E-04 | NM_001080941.1 | --- |
| Wnt7b | 1.20 | 3.3E-05 | NM_009528 | chr15 |
| Gcnt1 | 1.20 | 1.2E-05 | NM_173442 | chr19 |
| 9530008L14Rik | 1.21 | 6.3E-05 | BC027755 | chr13 |
| OTTMUSG00000015933 | 1.21 | 2.9E-05 | ENSMUST00000099133 | chr2 |
| Cdkn2b | 1.21 | 3.7E-05 | NM_007670 | chr4 |
| Cd300lb | 1.21 | 3.4E-04 | NM_199221 | chr11 |
| Rex2 | 1.22 | 1.6E-05 | NM_009051 | chr4 |
| Timp1 | 1.22 | 2.4E-04 | NM_001044384 | chrX |
| Acot2 | 1.22 | 4.1E-05 | NM_134188 | chr12 |
| Iah1 | 1.22 | 2.6E-05 | NM_026347 | chr12 |
| Prss22 | 1.22 | 1.7E-05 | NM_133731 | chr17 |
| Cyp39a1 | 1.23 | 1.0E-04 | NM_018887 | chr17 |
| Plaur | 1.23 | 4.9E-05 | NM_011113 | chr7 |
| Lgi3 | 1.23 | 2.3E-05 | NM_145219 | chr14 |
| Lad1 | 1.23 | 6.2E-06 | NM_133664 | chr1 |
| Dsp | 1.24 | 1.3E-04 | NM_023842 | chr13 |
| Gcnt3 | 1.24 | 6.0E-05 | NM_028087 | chr9 |
| Cyb5r1 | 1.24 | 1.3E-05 | NM_028057 | chr1 |
| Fbp2 | 1.24 | 8.4E-05 | NM_007994 | chr13 |
| Pim1 | 1.24 | 1.8E-05 | NM_008842 | chr17 |
| B4galnt3 | 1.24 | 6.9E-06 | NM_198884 | chr6 |
| Glul | 1.24 | 2.5E-05 | NM_008131 | chr1 |
| Cc2d2a | 1.24 | 5.4E-05 | NM_172274 | chr5 |
| Nckap1l | 1.24 | 2.2E-04 | NM_153505 | chr15 |
| Mtus1 | 1.25 | 1.8E-04 | NM_001005863 | chr8 |
| 6820445E23Rik | 1.25 | 6.1E-06 | AK135532 | chr11 |
| Shf | 1.25 | 2.0E-04 | NM_001013829 | chr2 |
| 4930503E14Rik | 1.25 | 2.3E-04 | NM_029131 | chr14 |
| 4930503E14Rik // 4930503E14Rik | 1.25 | 1.9E-04 | NM_029131 | chr14 |
| 4930503E14Rik // 4930503E14Rik | 1.25 | 1.6E-04 | NM_029131 | chr14 |
| Hagh | 1.25 | 8.7E-06 | NM_024284 | chr17 |
| 100039528 | 1.25 | 1.0E-04 | XM_001478816 | chr3 |
| Oplah | 1.25 | 1.0E-04 | NM_153122 | chr15 |
| Gjc1 | 1.25 | 4.5E-05 | NM_008122 | chr11 |
| Bco2 | 1.25 | 1.4E-05 | NM_133217 | chr9 |
| Arhgdib | 1.25 | 4.3E-05 | NM_007486 | chr6 |
| 8430408G22Rik | 1.25 | 3.9E-05 | BC058515 | chr6 |
| Klf6 | 1.26 | 6.1E-06 | NM_011803 | chr13 |
| ENSMUSG00000072602 | 1.26 | 3.4E-05 | NM_001098269 | chr14 |
| ENSMUSG00000072602 | 1.26 | 3.4E-05 | NM_001098269 | chr14 |
| Rgnef | 1.26 | 5.1E-05 | NM_012026 | chr13 |
| Mmp28 | 1.26 | 1.0E-05 | NM_080453 | chr11 |
| Bnip3 | 1.27 | 9.5E-05 | NM_009760 | chr7 |
| Sp140 | 1.27 | 1.6E-04 | NM_001013817 | chr8_random |
| Vegfa | 1.27 | 5.5E-06 | NM_001025250 | chr17 |
| Grasp | 1.27 | 2.6E-05 | NM_019518 | chr15 |
| Pwwp2b | 1.27 | 6.1E-06 | NM_001098636 | chr7 |
| Vldlr | 1.27 | 3.9E-05 | NM_013703 | chr19 |
| Plag1 | 1.27 | 3.9E-05 | NM_019969 | chr4 |
| Fam114a1 | 1.27 | 2.1E-05 | NM_026667 | chr5 |
| Slc20a1 | 1.27 | 5.3E-06 | NM_015747 | chr2 |
| Gpr39 | 1.27 | 2.2E-04 | BC085285 | chr1 |
| Lrrc26 | 1.27 | 5.4E-05 | NM_146117 | chr2 |
| Rhbdd1 | 1.27 | 6.0E-05 | NM_029777 | chr1 |
| AK220484 | 1.27 | 1.4E-04 | NM_001083628 | chr18 |
| Pip5k1b | 1.27 | 7.6E-05 | NM_008846 | chr19 |
| Wnt10a | 1.28 | 9.0E-05 | NM_009518 | chr1 |
| Rnf157 | 1.28 | 5.2E-05 | BC053070 | chr11 |
| Rhpn2 | 1.28 | 7.0E-06 | NM_027897 | chr7 |
| Endod1 | 1.28 | 8.8E-05 | NM_028013 | chr9 |
| Cdk6 | 1.28 | 1.9E-05 | NM_009873 | chr5 |
| Arhgef3 | 1.28 | 3.1E-05 | NM_027871 | chr14 |
| Krt78 | 1.29 | 3.4E-05 | NM_212487 | chr15 |
| Osbpl3 | 1.29 | 5.5E-06 | NM_027881 | chr6 |
| Kcne3 | 1.29 | 2.2E-04 | NM_020574 | chr7 |
| Slc6a9 | 1.29 | 6.8E-06 | NM_008135 | chr4 |
| Atp2a3 | 1.29 | 3.8E-05 | NM_016745 | chr11 |
| EG623849 | 1.29 | 1.4E-05 | NR_003596 | chr5 |
| 1300014I06Rik | 1.30 | 3.0E-05 | NM_025831 | chr13 |
| Cd81 | 1.30 | 4.7E-05 | NM_133655 | chr7 |
| Fgf2 | 1.30 | 8.6E-05 | NM_008006 | chr3 |
| Crim1 | 1.30 | 1.8E-05 | NM_015800 | chr17 |
| AW146242 | 1.30 | 1.4E-05 | BC024822 | chr6 |
| Pla2g7 | 1.30 | 1.7E-05 | NM_013737 | chr17 |
| Ppfibp1 | 1.30 | 7.3E-06 | NM_026221 | chr6 |
| Prl2c3 | 1.31 | 3.6E-04 | NM_011118 | chr13 |
| Kcne2 | 1.32 | 2.0E-05 | NM_134110 | chr16 |
| Ly6a | 1.32 | 9.7E-06 | NM_010738 | chr15 |
| Ptgds2 | 1.32 | 7.9E-05 | NM_019455 | chr6 |
| Ahr | 1.32 | 1.1E-05 | NM_013464 | chr12 |
| Gpr137b | 1.32 | 3.6E-04 | NM_031999 | chr13 |
| Slc2a1 | 1.32 | 1.8E-04 | NM_011400 | chr4 |
| Cryab | 1.32 | 1.7E-05 | NM_009964 | chr9 |
| Kctd11 | 1.32 | 2.3E-05 | NM_153143 | chr11 |
| Gys2 | 1.32 | 1.1E-04 | NM_145572 | chr6 |
| OTTMUSG00000010670 | 1.33 | 1.7E-05 | ENSMUST00000105723 | chr4 |
| St6galnac2 | 1.33 | 3.1E-05 | NM_009180 | chr11 |
| Ptpn13 | 1.34 | 7.4E-05 | NM_011204 | chr5 |
| Epn3 | 1.34 | 3.0E-05 | NM_027984 | chr11 |
| Rnf39 | 1.34 | 2.1E-05 | NM_001099632 | chr17 |
| 2810408A11Rik | 1.34 | 5.4E-05 | BC069874 | chr11 |
| Bmp2 | 1.34 | 1.8E-04 | NM_007553 | chr2 |
| Centb1 | 1.34 | 2.5E-05 | NM_153788 | chr11 |
| Sp140 | 1.35 | 4.5E-05 | NM_001013817 | chr1 |
| Rab27b | 1.35 | 7.1E-05 | NM_001082553 | chr18 |
| BC025446 | 1.35 | 6.2E-05 | BC025446 | chr15 |
| Inhba | 1.35 | 1.7E-05 | NM_008380 | chr13 |
| Me3 | 1.35 | 7.3E-06 | NM_181407 | chr7 |
| Sardh | 1.35 | 5.4E-05 | NM_138665 | chr2 |
| Oas3 | 1.36 | 8.8E-06 | NM_145226 | chr5 |
| Stom | 1.36 | 4.7E-06 | NM_013515 | chr2 |
| Smtn | 1.37 | 7.0E-06 | NM_013870 | chr11 |
| 3110043O21Rik | 1.38 | 1.6E-04 | BC076612 | chr4 |
| F3 | 1.38 | 4.1E-06 | NM_010171 | chr3 |
| Dock8 | 1.38 | 1.0E-05 | NM_028785 | chr19 |
| G930009F23Rik | 1.39 | 1.3E-04 | AK145170 | chr15 |
| C130092O11Rik | 1.39 | 5.1E-05 | BC107398 | chr6 |
| Selenbp1 | 1.39 | 4.7E-05 | NM_009150 | chr3 |
| Acox2 | 1.39 | 2.0E-04 | NM_053115 | chr14 |
| Igsf5 | 1.39 | 9.2E-06 | NM_028078 | chr16 |
| Pycr1 | 1.40 | 2.8E-05 | NM_144795 | chr11 |
| Hpse | 1.40 | 8.4E-05 | NM_152803 | chr5 |
| Sh2d4a | 1.40 | 1.8E-05 | NM_028182 | chr8 |
| Myl7 | 1.41 | 2.3E-05 | NM_022879 | chr11 |
| Emp1 | 1.41 | 1.4E-05 | NM_010128 | chr6 |
| Dhrs9 | 1.42 | 1.9E-04 | NM_175512 | chr2 |
| --- | 1.43 | 4.1E-06 | ENSMUST00000049821 | chr3 |
| Rhof | 1.43 | 5.7E-05 | NM_175092 | chr5 |
| Marcks | 1.43 | 2.8E-05 | NM_008538 | chr10 |
| Chac1 | 1.43 | 2.8E-05 | NM_026929 | chr2 |
| Cldn8 | 1.43 | 4.3E-05 | NM_018778 | chr16 |
| Bmf | 1.43 | 9.0E-05 | NM_138313 | chr2 |
| Bcl2l11 | 1.43 | 5.5E-06 | NM_207680 | chr2 |
| Zc3h6 | 1.44 | 7.0E-05 | NM_178404 | chr2 |
| AW112010 | 1.44 | 2.6E-05 | EF660528 | chr19 |
| --- | 1.44 | 3.1E-04 | ENSMUST00000100997 | chr5 |
| Gsdmd | 1.45 | 9.1E-06 | NM_026960 | chr15 |
| Heph | 1.45 | 9.7E-06 | NM_010417 | chrX |
| Fam84a | 1.46 | 1.1E-04 | NM_029007 | chr12 |
| Rgs16 | 1.46 | 1.2E-05 | NM_011267 | chr1 |
| Slamf9 | 1.46 | 2.7E-06 | NM_029612 | chr1 |
| OTTMUSG00000015351 | 1.46 | 3.1E-05 | ENSMUST00000099395 | chr2 |
| Vill | 1.46 | 3.4E-06 | NM_011700 | chr9 |
| Lilrb4 | 1.47 | 1.1E-05 | NM_013532 | chr10 |
| Casp12 | 1.47 | 1.1E-04 | NM_009808 | chr9 |
| Mpzl3 | 1.48 | 7.5E-06 | NM_176993 | chr9 |
| Gabarapl1 | 1.48 | 1.6E-04 | NM_020590 | chr6 |
| 2310045A20Rik | 1.48 | 5.3E-06 | NM_172710 | chr5 |
| Pold4 | 1.48 | 1.4E-05 | NM_027196 | chr19 |
| Il23a | 1.49 | 1.2E-05 | NM_031252 | chr10 |
| Gulo | 1.49 | 1.4E-05 | NM_178747 | chr14 |
| 9030420J04Rik | 1.49 | 2.8E-06 | BC137891 | chr9 |
| Bhlhe40 | 1.49 | 2.3E-05 | NM_011498 | chr6 |
| --- | 1.49 | 2.3E-04 | mmu-mir-568 // mmu-mir-568 | chr16 |
| Lbp | 1.50 | 1.0E-05 | NM_008489 | chr2 |
| Clic5 | 1.50 | 3.1E-06 | NM_172621 | chr17 |
| Tnfrsf22 | 1.50 | 2.7E-06 | NM_023680 | chr7 |
| Lman1l | 1.51 | 1.4E-05 | NM_199222 | chr9 |
| Capn5 | 1.51 | 3.5E-06 | NM_007602 | chr7 |
| EG623849 // EG623849 | 1.51 | 2.4E-04 | NR_003596 | chr5 |
| Cav2 | 1.52 | 4.1E-05 | NM_016900 | chr6 |
| Rnasel | 1.52 | 4.3E-05 | NM_011882 | chr1 |
| Tnfaip8 | 1.52 | 3.9E-05 | NM_134131 | chr18 |
| Fhl1 | 1.52 | 1.5E-05 | NM_001077361 | chrX |
| AU021092 | 1.53 | 4.1E-05 | NM_001033220 | chr16 |
| Adcy3 | 1.53 | 1.4E-05 | NM_138305 | chr12 |
| Rab11fip2 | 1.53 | 4.3E-05 | NM_001033172 | chr19 |
| Npal1 | 1.53 | 8.6E-05 | NM_001081205 | chr5 |
| Ccng2 | 1.53 | 1.7E-05 | NM_007635 | chr5 |
| Pcsk6 | 1.53 | 1.9E-06 | BC037450 | chr7 |
| Cd74 | 1.54 | 2.3E-04 | NM_001042605 | chr18 |
| Nrp2 | 1.54 | 1.4E-05 | NM_001077403 | chr1 |
| Xpnpep2 | 1.54 | 9.2E-06 | NM_133213 | chrX |
| Xlr3c | 1.55 | 1.7E-05 | NM_011727 | chrX |
| Mal | 1.55 | 6.1E-06 | NM_010762 | chr2 |
| Pcolce | 1.56 | 3.2E-06 | NM_008788 | chr5 |
| Gsto2 | 1.57 | 1.4E-05 | NM_026619 | chr19 |
| Gpt2 | 1.57 | 1.4E-04 | NM_173866 | chr8 |
| Sqrdl | 1.57 | 2.9E-05 | NM_021507 | chr2 |
| Nt5e | 1.57 | 3.1E-05 | NM_011851 | chr9 |
| Oit1 | 1.58 | 4.2E-06 | NM_146050 | chr14 |
| Pcdh1 | 1.58 | 1.4E-04 | NM_029357 | chr18 |
| Prdm1 | 1.58 | 1.6E-05 | NM_007548 | chr10 |
| Lgals4 | 1.58 | 5.7E-05 | NM_010706 | chr7 |
| P4ha2 | 1.59 | 1.8E-05 | NM_001136076 | chr11 |
| Tmem71 | 1.59 | 2.1E-05 | NM_172514 | chr15 |
| Pion | 1.59 | 8.9E-06 | NM_175437 | chr5 |
| Dyrk4 | 1.59 | 7.0E-06 | NM_207210 | chr6 |
| Cyb5r2 | 1.59 | 1.7E-05 | NM_177216 | chr7 |
| Xlr3b | 1.60 | 2.0E-05 | NM_001081643 | chrX |
| Aim2 | 1.60 | 2.2E-05 | NM_001013779 | chr1 |
| Slc22a4 | 1.61 | 1.2E-05 | NM_019687 | chr11 |
| Slc6a13 | 1.61 | 5.3E-06 | NM_144512 | chr6 |
| Bcar3 | 1.62 | 2.8E-06 | NM_013867 | chr3 |
| Fgfr3 | 1.62 | 2.3E-06 | NM_008010 | chr5 |
| Gtpbp2 | 1.63 | 1.6E-05 | NM_019581 | chr17 |
| Camk1d | 1.63 | 5.3E-06 | NM_177343 | chr2 |
| Sema3c | 1.64 | 7.8E-06 | NM_013657 | chr5 |
| Lgr6 | 1.64 | 3.9E-06 | NM_001033409 | chr1 |
| Ets2 | 1.64 | 7.2E-06 | NM_011809 | chr16 |
| Phldb2 | 1.64 | 7.5E-06 | NM_153412 | chr16 |
| Krt14 | 1.64 | 1.0E-05 | NM_016958 | chr11 |
| Tnfrsf26 | 1.65 | 5.5E-06 | NM_175649 | chr7 |
| Bst1 | 1.65 | 6.4E-05 | NM_009763 | chr5 |
| --- | 1.65 | 4.2E-06 | EU675311 | chr16 |
| --- | 1.66 | 2.3E-05 | ENSMUST00000100687 | chr14 |
| Fgf21 | 1.67 | 8.7E-05 | NM_020013 | chr7 |
| Hbegf | 1.67 | 1.7E-06 | NM_010415 | chr18 |
| Sigirr | 1.67 | 2.2E-06 | NM_023059 | chr7 |
| Klhl24 | 1.67 | 2.8E-06 | NM_029436 | chr16 |
| Serpine1 | 1.68 | 3.1E-05 | NM_008871 | chr5 |
| Gm73 | 1.68 | 3.1E-06 | AK157788 | chr13 |
| Abcb1a | 1.68 | 4.1E-06 | NM_011076 | chr5 |
| Serpinb7 | 1.69 | 7.0E-05 | NM_027548 | chr1 |
| Il18 | 1.69 | 7.4E-05 | NM_008360 | chr9 |
| Sh3tc2 | 1.69 | 3.9E-06 | NM_172628 | chr18 |
| --- | 1.70 | 8.4E-05 | AK044970 | chr17 |
| --- | 1.70 | 8.4E-05 | AK044970 | chr5 |
| --- | 1.70 | 8.4E-05 | AK044970 | chr6 |
| C3 | 1.70 | 6.9E-06 | NM_009778 | chr17 |
| Usp27x | 1.70 | 3.9E-06 | AF229643 | chrX |
| Crlf1 | 1.71 | 9.6E-06 | NM_018827 | chr8 |
| Nrip2 | 1.71 | 3.1E-06 | NM_021717 | chr6 |
| Klrc1 | 1.72 | 3.1E-05 | NM_001136068 | chr6 |
| Lamb3 | 1.74 | 7.3E-06 | NM_008484 | chr1 |
| Cldn4 | 1.74 | 2.6E-05 | NM_009903 | chr5 |
| Pfkp | 1.74 | 1.4E-05 | NM_019703 | chr13 |
| Cdc42ep5 | 1.74 | 4.3E-05 | NM_021454 | chr7 |
| ENSMUSG00000072602 | 1.75 | 4.2E-05 | NM_001098269 | chr14 |
| Stbd1 | 1.75 | 2.4E-06 | NM_175096 | chr5 |
| Ankrd22 | 1.76 | 1.1E-05 | NM_024204 | chr19 |
| Nbl1 | 1.76 | 6.6E-06 | NM_008675 | chr4 |
| D8Ertd82e | 1.77 | 3.3E-06 | NM_172911 | chr8 |
| Mrgprb2 | 1.78 | 1.2E-05 | NM_175531 | chr7 |
| Pdgfb | 1.79 | 2.9E-06 | NM_011057 | chr15 |
| Scel | 1.79 | 1.7E-06 | NM_022886 | chr14 |
| Garnl4 | 1.79 | 3.9E-06 | NM_001015046 | chr11 |
| Cxcl17 | 1.79 | 1.1E-05 | NM_153576 | chr7 |
| Cdh13 | 1.80 | 6.7E-06 | NM_019707 | chr8 |
| Abcb1b | 1.80 | 2.1E-05 | NM_011075 | chr5 |
| Heg1 | 1.81 | 5.7E-05 | NM_175256 | chr16 |
| Stra6 | 1.81 | 2.3E-06 | NM_009291 | chr9 |
| 9030420J04Rik | 1.81 | 1.3E-06 | BC137891 | chr9 |
| Pglyrp1 | 1.82 | 1.2E-06 | NM_009402 | chr7 |
| Hk2 | 1.82 | 2.8E-06 | NM_013820 | chr6 |
| Zfp345 | 1.83 | 4.2E-05 | NM_001034900 | chr2 |
| Zbtb20 | 1.83 | 3.1E-06 | NM_019778 | chr16 |
| Xlr3a | 1.83 | 2.8E-05 | NM_001110784 | chrX |
| Rab11fip5 | 1.84 | 1.4E-06 | NM_001003955 | chr6 |
| Ddr2 | 1.84 | 6.6E-06 | NM_022563 | chr1 |
| Eda2r | 1.85 | 2.0E-04 | NM_175540 | chrX |
| 9930111J21Rik | 1.85 | 2.2E-06 | NM_173434 | chr11 |
| Nrn1 | 1.85 | 1.1E-05 | NM_153529 | chr13 |
| Fxyd5 | 1.85 | 2.0E-06 | NM_008761 | chr7 |
| Plce1 | 1.85 | 1.8E-06 | NM_019588 | chr19 |
| 9930111J21Rik | 1.85 | 2.5E-06 | NM_173434 | chr11 |
| Ltbp1 | 1.85 | 5.5E-06 | NM_019919 | chr17 |
| Nlrp10 | 1.86 | 1.2E-05 | NM_175532 | chr7 |
| Prkcbp1 | 1.86 | 1.7E-05 | NM_027230 | chr2 |
| Trib3 | 1.86 | 5.9E-06 | NM_175093 | chr2 |
| Ass1 | 1.87 | 2.4E-06 | NM_007494 | chr10 |
| Gpr141 | 1.87 | 3.9E-06 | NM_181754 | chr13 |
| Kcnn4 | 1.88 | 2.3E-05 | NM_008433 | chr7 |
| Ass1 | 1.88 | 2.1E-06 | NM_007494 | chr2 |
| Sprr1a | 1.88 | 3.0E-05 | NM_009264 | chr3 |
| B4galnt2 | 1.89 | 1.3E-05 | NM_008081 | chr11 |
| Eno2 | 1.90 | 7.4E-06 | NM_013509 | chr6 |
| Gpd1 | 1.90 | 6.9E-06 | NM_010271 | chr15 |
| Slc14a1 | 1.90 | 6.8E-06 | NM_028122 | chr18 |
| Atp10b | 1.90 | 4.5E-06 | NM_176999 | chr11 |
| Naip2 | 1.91 | 1.1E-06 | NM_010872 | chr13 |
| Angpt1 | 1.91 | 4.5E-05 | NM_009640 | chr15 |
| Naip5 | 1.92 | 1.6E-04 | NM_010870 | chr13 |
| 1600029D21Rik | 1.92 | 5.9E-06 | NM_029639 | chr9 |
| Trim29 | 1.93 | 2.8E-06 | NM_023655 | chr9 |
| Pstpip2 | 1.93 | 1.7E-06 | NM_013831 | chr18 |
| Ero1l | 1.94 | 2.8E-06 | NM_015774 | chr14 |
| Tgm2 | 1.94 | 1.9E-06 | NM_009373 | chr2 |
| F5 | 1.95 | 1.6E-05 | NM_007976 | chr1 |
| Spns2 | 1.95 | 1.1E-06 | BC025823 | chr11 |
| Mrgprb3 | 1.95 | 9.5E-05 | NM_207537 | chr7 |
| Tmem171 | 1.96 | 4.5E-06 | NM_001025606 | chr13 |
| Ccl20 | 1.96 | 7.3E-06 | NM_016960 | chr1 |
| Trim30 | 1.96 | 1.3E-04 | NM_009099 | chr7 |
| Ttc9 | 1.96 | 8.6E-07 | NM_001033149 | chr12 |
| Gch1 | 1.97 | 2.9E-04 | NM_008102 | chr14 |
| Zbtb20 | 1.99 | 3.4E-05 | ENSMUST00000033479 | chr16 |
| Ly6g | 1.99 | 4.5E-06 | ENSMUST00000023246 | chr15 |
| Pkp1 | 2.00 | 4.1E-06 | NM_019645 | chr1 |
| Olfr1372-ps1 | 2.01 | 2.6E-06 | BC055827 | chr11 |
| Il18rap | 2.02 | 1.2E-06 | NM_010553 | chr1 |
| Gja1 | 2.02 | 1.6E-04 | NM_010288 | chr10 |
| Mettl7b | 2.03 | 1.8E-06 | NM_027853 | chr10 |
| Tmem117 | 2.05 | 5.8E-06 | NM_178789 | chr15 |
| OTTMUSG00000010657 | 2.05 | 2.2E-04 | NM_001083918 | chr4 |
| Unc5b | 2.06 | 3.3E-06 | NM_029770 | chr10 |
| Pmepa1 | 2.06 | 8.9E-07 | NM_022995 | chr2 |
| F2rl2 | 2.06 | 7.3E-06 | NM_010170 | chr13 |
| 1700029I01Rik | 2.06 | 1.1E-06 | NM_027285 | chr4 |
| --- | 2.07 | 7.9E-05 | ENSMUST00000100697 | chr14 |
| Syt8 | 2.09 | 2.2E-06 | NM_018802 | chr7 |
| Ceacam1 | 2.09 | 4.5E-05 | NM_001039185 | chr7 |
| Paqr5 | 2.09 | 8.7E-06 | NM_028748 | chr9 |
| Ptgs1 | 2.10 | 9.7E-06 | NM_008969 | chr2 |
| Cav1 | 2.11 | 8.5E-06 | NM_007616 | chr6 |
| S100a14 | 2.11 | 2.3E-06 | NM_025393 | chr3 |
| 672682 | 2.11 | 1.2E-06 | AB242596 | chr17 |
| Ankrd37 | 2.12 | 1.1E-06 | NM_001039562 | chr8 |
| Muc5b | 2.13 | 3.9E-06 | NM_028801 | chr7 |
| Msln | 2.13 | 1.3E-05 | NM_018857 | chr17 |
| Il34 | 2.13 | 6.2E-06 | NM_029646 | chr8 |
| Ktelc1 | 2.15 | 5.5E-07 | NM_172380 | chr16 |
| --- | 2.16 | 2.0E-06 | NM_010400.1 | --- |
| Depdc6 | 2.16 | 2.0E-05 | NM_001037937 | chr15 |
| Steap1 | 2.17 | 1.2E-06 | NM_027399 | chr5 |
| Bcas1 | 2.18 | 1.8E-06 | NM_029815 | chr2 |
| Tmem45a | 2.21 | 2.6E-05 | NM_019631 | chr16 |
| Tnip3 | 2.22 | 8.7E-06 | NM_001001495 | chr6 |
| OTTMUSG00000011097 | 2.23 | 3.2E-06 | NM_001085522 | chr4 |
| Cd82 | 2.23 | 9.2E-07 | NM_007656 | chr2 |
| Capn6 | 2.23 | 6.1E-06 | NM_007603 | chrX |
| Htra1 | 2.26 | 6.5E-07 | NM_019564 | chr7 |
| Krt79 | 2.26 | 1.2E-06 | NM_146063 | chr15 |
| Pmp22 | 2.27 | 1.3E-05 | NM_008885 | chr11 |
| Arap2 | 2.27 | 1.6E-06 | NM_178407 | chr5 |
| --- | 2.27 | 3.2E-06 | ENSMUST00000100698 | chr14 |
| Odf3l1 | 2.29 | 1.7E-06 | NM_198673 | chr9 |
| 2310043J07Rik | 2.29 | 6.9E-06 | BC115564 | chr5 |
| Papss2 | 2.30 | 7.0E-06 | NM_011864 | chr19 |
| Il24 | 2.31 | 7.0E-06 | NM_053095 | chr1 |
| Serpinb6b | 2.31 | 1.3E-05 | NM_011454 | chr13 |
| Serpinb8 | 2.31 | 4.6E-06 | NM_011459 | chr1 |
| A930038C07Rik | 2.32 | 9.2E-07 | BC047154 | chr6 |
| Casp1 | 2.32 | 3.0E-07 | NM_009807 | chr9 |
| Angptl2 | 2.33 | 1.8E-06 | NM_011923 | chr2 |
| Ecm1 | 2.34 | 1.3E-06 | NM_007899 | chr3 |
| 2310002L13Rik | 2.34 | 9.7E-06 | ENSMUST00000025390 | chr18 |
| Tnnt2 | 2.35 | 5.3E-07 | NM_001130174 | chr1 |
| Gramd1b | 2.35 | 8.5E-07 | NM_172768 | chr9 |
| Tns4 | 2.35 | 3.3E-06 | NM_172564 | chr11 |
| Ifi204 | 2.37 | 3.9E-06 | NM_008329 | chr1 |
| Agxt2l1 | 2.38 | 6.6E-06 | NM_027907 | chr3 |
| --- | 2.38 | 9.2E-07 | NM_008818.2 | --- |
| Gp49a | 2.39 | 1.7E-06 | NM_008147 | chr10 |
| 2610528A11Rik | 2.44 | 3.0E-06 | AK012157 | chr14 |
| Cpe | 2.45 | 9.7E-07 | NM_013494 | chr8 |
| Steap2 | 2.45 | 3.0E-07 | NM_001103157 | chr5 |
| Cyp2s1 | 2.47 | 1.1E-06 | NM_028775 | chr7 |
| Car5b | 2.47 | 7.4E-07 | NM_181315 | chrX |
| 5430435G22Rik | 2.50 | 5.5E-06 | NM_145509 | chr1 |
| Ifi202b | 2.51 | 1.9E-06 | NM_008327 | chr1 |
| Anxa8 | 2.51 | 9.4E-07 | NM_013473 | chr14 |
| Spnb2 | 2.55 | 1.2E-06 | NM_175836 | chr11 |
| Jag1 | 2.57 | 4.8E-07 | NM_013822 | chr2 |
| Il1rn | 2.57 | 1.7E-06 | NM_031167 | chr2 |
| Gpa33 | 2.60 | 3.9E-07 | NM_021610 | chr1 |
| Upk1b | 2.60 | 1.7E-06 | NM_178924 | chr16 |
| Sema3e | 2.60 | 9.2E-07 | NM_011348 | chr5 |
| Acsbg1 | 2.60 | 9.2E-07 | NM_053178 | chr9 |
| Ifi203 | 2.60 | 4.3E-05 | NM_001045481 | chr1 |
| Sh2d1b1 | 2.64 | 1.4E-06 | NM_012009 | chr1 |
| 2610305D13Rik | 2.65 | 2.7E-06 | NM_145078 | chr4 |
| Ly6c1 | 2.65 | 4.5E-07 | NM_010741 | chr15 |
| Pgcp | 2.65 | 1.1E-06 | NM_018755 | chr15 |
| Nupr1 | 2.66 | 5.2E-06 | NM_019738 | chr7 |
| Ly6c1 | 2.69 | 3.7E-07 | NM_010741 | chr15 |
| Wnt7a | 2.71 | 1.1E-06 | NM_009527 | chr6 |
| Mmp13 | 2.79 | 1.1E-06 | NM_008607 | chr9 |
| EG639116 | 2.80 | 1.7E-06 | XM_001475880 | chr7 |
| Mme | 2.84 | 6.2E-07 | NM_008604 | chr3 |
| 9230104L09Rik | 2.84 | 2.7E-06 | NM_029960 | chr2 |
| Txnip | 2.85 | 3.2E-06 | NM_001009935 | chr3 |
| Trpv6 | 2.85 | 1.5E-06 | NM_022413 | chr6 |
| Serpinb2 | 2.87 | 7.0E-06 | NM_011111 | chr1 |
| Qpct | 2.87 | 1.4E-06 | NM_027455 | chr17 |
| Thbs1 | 2.91 | 3.7E-07 | NM_011580 | chr2 |
| Serpinb6c | 2.94 | 2.1E-07 | NM_148942 | chr13 |
| Adamtsl3 | 2.97 | 5.5E-06 | AK220376 | chr7 |
| Gadd45a | 3.00 | 7.8E-07 | NM_007836 | chr6 |
| Ndrg1 | 3.18 | 1.1E-07 | NM_008681 | chr15 |
| Spp1 | 3.19 | 3.5E-07 | NM_009263 | chr5 |
| Tgfbi | 3.26 | 2.1E-07 | NM_009369 | chr13 |
| Serpinb5 | 3.38 | 3.8E-07 | NM_009257 | chr1 |
| Ctse | 3.53 | 3.0E-07 | NM_007799 | chr1 |
| Serpinb11 | 3.57 | 2.8E-06 | NM_025867 | chr1 |
| Car6 | 3.91 | 2.1E-07 | NM_009802 | chr4 |
| Capg | 4.08 | 2.1E-07 | NM_007599 | chr6 |
| Dsg3 | 4.38 | 1.2E-07 | NM_030596 | chr18 |
|  |  |  |  |  |
|  |  |  |  |  |
