# Supplementary material for: Transcription factor and microRNA interactions in lung cells: an inhibitory link between NK2 homeobox 1, miR-200c and the developmental and oncogenic factors Nfib and Myb
Source: Respir Res. 2015 Feb 13;16(1):22. doi: 10.1186/s12931-015-0186-6 (PMC4335692; doi:10.1186/s12931-015-0186-6)
Supplement: Additional file 4: Table S3. — Gene ontology analysis of predicted miR-200c targets down-regulated in Nkx2-1 knock-down cells. [file 12931_2015_186_MOESM4_ESM.docx]

| *Table S3. Gene ontology analysis of predicted miR-200c targets down-regulated in Nkx2-1 knock-down cells.* | | | |
| --- | --- | --- | --- |
| *Bayes Factor >3.00, > 5 genes per GO group* | | | |
| # | Annotation | Bayes factor | |
| 1 | GO:0007275 [2]: development | | 5.36 |
| 2 | GO:0050789 [2]: regulation of biological process | | 4.46 |
| 3 | GO:0009653 [3]: morphogenesis | | 4.34 |
| 5 | GO:0006355 [7]: regulation of transcription, DNA-dependent | | 4.1 |
| 7 | GO:0006139 [5]: nucleobase, nucleoside, nucleotide and nucleic acid metabolism | | 4 |
| 8 | GO:0045449 [6]: regulation of transcription | | 3.98 |
| 9 | GO:0006351 [7]: transcription, DNA-dependent | | 3.98 |
| 10 | GO:0019219 [5]: regulation of nucleobase, nucleoside, nucleotide and nucleic acid metab | | 3.96 |
| 11 | GO:0006350 [6]: transcription | | 3.76 |
| 12 | GO:0050791 [3]: regulation of physiological process | | 3.68 |
| 16 | GO:0019222 [4]: regulation of metabolism | | 3.44 |
| 17 | GO:0009887 [4]: organogenesis | | 3.34 |
| 18 | GO:0048513 [3]: organ development | | 3.31 |
